# Epigenetic Regulation by BAF Complexes Limits Neural Stem Cell Proliferation by Suppressing Wnt Signaling in Late Embryonic Development

**DOI:** 10.1016/j.stemcr.2018.04.014

**Published:** 2018-05-17

**Authors:** Huong Nguyen, Cemil Kerimoglu, Mehdi Pirouz, Linh Pham, Kamila A. Kiszka, Godwin Sokpor, M. Sadman Sakib, Joachim Rosenbusch, Ulrike Teichmann, Rho H. Seong, Anastassia Stoykova, Andre Fischer, Jochen F. Staiger, Tran Tuoc

**Affiliations:** 1 Institute of Neuroanatomy, University Medical Center, Georg-August- University, 37075 Goettingen, Germany; 2 Department of Psychiatry and Psychotherapy, University Medical Center, Georg- August-University Goettingen, 37077 Goettingen, Germany; 3 Max-Planck-Institute for Biophysical Chemistry, 37077 Goettingen, Germany; 4 DFG Center for Nanoscale Microscopy & Molecular Physiology of the Brain (CNMPB), 37075 Goettingen, Germany; 5 Department of Biological Sciences, Institute of Molecular Biology and Genetics, Research Center for Functional Cellulomics, Seoul National University, Seoul 151- 742, Korea; 6 Department for Systems Medicine and Epigenetics, German Center for Neurodegenerative Diseases, 37075 Goettingen, Germany

**Keywords:** cortical development, hippocampal development, neurogenesis, neural stem cells, chromatin remodeling, BAF (mSWI/SNF) complexes, epigenetics, H3K4me2, H3K27me3

## Abstract

During early cortical development, neural stem cells (NSCs) divide symmetrically to expand the progenitor pool, whereas, in later stages, NSCs divide asymmetrically to self-renew and produce other cell types. The timely switch from such proliferative to differentiative division critically determines progenitor and neuron numbers. However, the mechanisms that limit proliferative division in late cortical development are not fully understood. Here, we show that the BAF (mSWI/SNF) complexes restrict proliferative competence and promote neuronal differentiation in late corticogenesis. Inactivation of BAF complexes leads to H3K27me3-linked silencing of neuronal differentiation-related genes, with concurrent H3K4me2-mediated activation of proliferation-associated genes via de-repression of Wnt signaling. Notably, the deletion of BAF complexes increased proliferation of neuroepithelial cell-like NSCs, impaired neuronal differentiation, and exerted a Wnt-dependent effect on neocortical and hippocampal development. Thus, these results demonstrate that BAF complexes act as both activators and repressors to control global epigenetic and gene expression programs in late corticogenesis.

## Highlights

• Loss of BAF complexes increases H3K27me3 and H3K4me2 marks in late corticogenesis
• BAF complexes epigenetically regulate neural proliferation and differentiation
• BAF complexes suppress neuroepithelial cell fate and Wnt signaling
• BAF complexes control cortical development in a Wnt signaling-dependent manner

## Graphical Abstract

**Figure:**
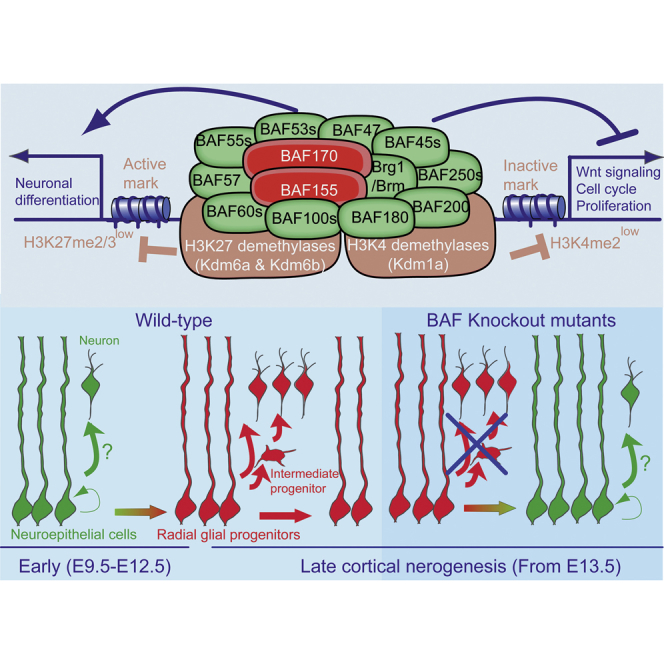

## Teaser

In this article, Tuoc and colleagues show that inactivation of BAF complexes in late cortical development leads to H3K27me3-linked silencing of neuronal differentiation-related genes, with concurrent H3K4me2-mediated activation of proliferation-associated genes via de-repression of Wnt signaling. The deletion of BAF complexes increased proliferation of neuroepithelial-like progenitors, impaired neuronal differentiation, and exerted a Wnt-dependent effect on neocortical and hippocampal development.

## Introduction

During vertebrate cerebral cortex development, neural stem cells (NSCs) undergo two types of temporally regulated cell division modes to generate distinct neural cell types. During early corticogenesis in mice (embryonic day 8.5–12.5 [E8.5–E12.5]), NSCs, also called neuroepithelial cells (NEs), mainly divide symmetrically to proliferate and expand their population (Dehay and Kennedy, 2007, Gotz and Huttner, 2005, Kriegstein and Alvarez-Buylla, 2009, Martynoga et al., 2012, Tuoc et al., 2014). At the onset of neurogenesis (E10.5), NEs differentiate into mature NSCs, also termed radial glial progenitors (RGs), which start to express astroglial markers (Hartfuss et al., 2001). This process coincides with the loss and appearance of tight and adherens junctional complexes respectively in the ventricular zone (VZ) (Aaku-Saraste et al., 1996, Sahara and O'Leary, 2009). Later, RGs primarily divide asymmetrically to produce an RG to maintain the proliferative pool, and either an excitatory neuron or a basal progenitor. Delayed RG differentiation from NEs causes aberrant neurogenesis (Sahara and O'Leary, 2009), yet factors that are required to suppress NE fate in late corticogenesis to ensure a balance between NSC proliferation and neuronal differentiation are unknown.

The temporal relationship and intricate balance between proliferative symmetric and neurogenic asymmetric divisions in the VZ of the cortex is controlled by diverse signaling pathways. Among these, Wnt/β-catenin signaling has been extensively investigated for its role in proliferative symmetric division (Chenn and Walsh, 2002). For example, elevation of Wnt signaling through overexpression of β-catenin massively enhanced cortical NSC proliferation (Chenn and Walsh, 2002). Interestingly, a recent study revealed irreversibility of the progression from proliferative to neurogenic division modes, thus implicating a default program in NSCs for division-mode transition during corticogenesis (Gao et al., 2014). As regulators of the spatiotemporal expression of developmental genes, epigenetic and chromatin regulatory mechanisms have been proposed to contribute to establishing the proliferative and differentiation competence of NSCs (Hirabayashi and Gotoh, 2010, Yao et al., 2016).

To investigate the possible involvement of chromatin-remodeling BAF (mSWI/SNF) complexes in this process, we applied a conditional deletion approach through double-knockout (dcKO) of the BAF155 and BAF170 subunits, which eliminate the entire BAF complex during late cortical neurogenesis in transgenic mice. In the absence of BAF complexes, transcriptional profiling and epigenetic analyses revealed an enrichment of downregulated RG (astroglial, adherens junctions)- and neuronal differentiation-related genes, with both gene groups showing increased H3K27me3 repressive marks. In contrast, upregulated genes with increased H3K4me2 active marks were predominantly involved in the regulation of NE cell fate (e.g., tight junction feature), proliferation, cell cycle, and Wnt signaling-related pathways. The results of this study suggest that BAF complexes exert genome-wide control on both active H3K4me2 and repressive H3K27me3 marks during late cortical development by directly interacting with the corresponding H3 demethylases and regulating their activity. Phenotypically, we found that deletion of BAF complexes during late cortical neurogenesis leads to dysgenesis of the upper cortical layers and the hippocampal formation. These perturbations were rescued by inhibition of Wnt/β-catenin signaling. Together, these observations provide insights into distinct epigenetic regulatory mechanisms mediated by chromatin-remodeling BAF complexes as a key factor that suppresses the proliferative competence of NSCs during late cortical development.

## Results

### Loss of BAF Complexes Causes a Genome-wide Increase in the Level of Both Active and Repressive Epigenetic Marks at Distinct Loci in the Developing Pallium during Late Neurogenesis

**Figure 1:**
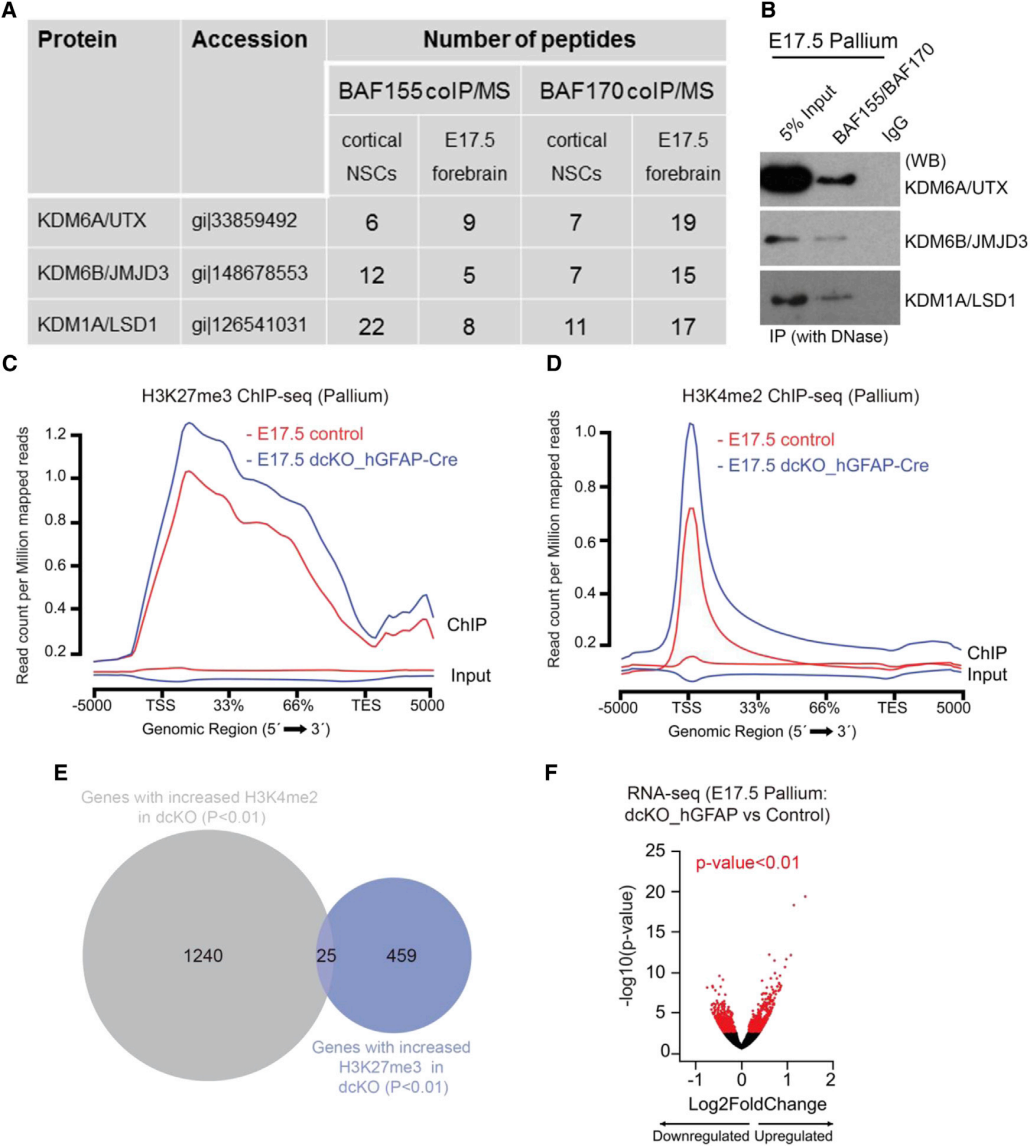
BAF Complexes Globally Control Epigenetic and Gene Expression Programs in Late Development Pallium (A) Table showing the peptide number for KDM6A, KDM6B, and KDM1A proteins purified from BAF155 and BAF170 immunoprecipitates of protein extracts from NS5 cells, E13.5 or E17.5 forebrain. (B) Interactions of BAF155 and BAF170 with KDM6A, KDM6B, and KDM1A were confirmed by coIP/western blot (WB) analyses of E17.5 pallium tissue. (C–E) Distribution of H3K27me3 (C) and H3K4me2 (D) marks along gene bodies in the dcKO and control pallium at E17.5. H3K27me3 levels are increased in dcKOs. dcKO (E) genes with increased H3K4me2 or H3K27me3 marks in the dcKO pallium at E17.5 are largely non-overlapping. (F) Volcano plot representing differentially regulated genes in the dcKO pallium at E17.5. Experimental replicates (n) = 4 (C, D; ChIP-seq), 4 (control for RNA-seq; F), 3 (dcKO_hGFAP-Cre for RNA-seq; F).

We previously reported that BAF complexes potentiate the activity of two main H3K27 demethylases, JMJD3 and UTX. Accordingly, elimination of BAF complexes during early corticogenesis leads to a global increase in repressive marks (H3K27Me2/3) and downregulation of gene expression at E13.5 (Narayanan et al., 2015, Nguyen et al., 2016). In further analysis, we performed co-immunoprecipitation (coIP) experiments on tissue lysates from the pallium of E17.5 wild-type (WT) embryos followed by mass spectrometry to identify BAF155/BAF170-interacting proteins. At E17.5, we found that BAF155 and BAF170 bind to the H3K27me2/3 demethylases, UTX/KDM6A and JMJD3/KDM6B, as shown in our previous study at E13.5 (Narayanan et al., 2015). BAF155/BAF170 was also observed to interact with H3K4me1/2 demethylase LSD1/KDM1A in the E17.5 pallium (Figures 1A, 1B, and S1A).

To investigate if BAF complexes regulate epigenetic programs in late cortical development, we crossed *Baf155*-floxed (*Baf155*^*fl/fl*^) mice and *Baf170*-floxed (*Baf170*^*fl/fl*^) mice with the *hGFAP-*Cre line to generate *dcKO* mutants. In contrast to the *Emx1*-Cre line used in our previous studies (Narayanan et al., 2015, Tuoc et al., 2009, Tuoc et al., 2013) with Cre recombination in the developing cortex as early as E10.5, the *hGFAP* promoter is not active in the pallium prior to E12.5. At E13.5, *hGFAP*-Cre activity is restricted to the medial pallium (MP), containing the hippocampal anlage and medial cortex (Figure S1B). From E15.5 onward, *hGFAP*-Cre activity extends to the dorsal pallium (DP; dorsal cortex) and lateral pallium (LP; lateral cortex) during development (Figure S1C). BAF155 and BAF170 proteins were not detected in the MP of dcKO mutants from E14.5 or in the entire VZ of the pallium from E15.5 onward (Figure S1D) (Narayanan et al., 2015), hence validating our *Baf155/Baf170* knockout system in late pallial progenitors.

Given the identified interaction of BAF complexes with the H3K27me2/3 demethylases KDM6A/B and H3K4me1/2 demethylase KDM1A in the E17.5 pallium, we next compared H3K27me3 repressive and H3K4me2 activatory marks in the E17.5 *dcKO* and control pallia. As reported previously, loss of BAF complexes in the E13.5 murine pallium in dcKO_*Emx1*-Cre mutants results in an increase in H3K27me3 levels (Narayanan et al., 2015). Similarly, chromatin immunoprecipitation sequencing (ChIP-seq) analysis performed using chromatin isolated from the E17.5 *dcKO* pallium also revealed an increase in H3K27me3 upon *Baf155/170* knockout (Figure 1C). Specifically, 181 genes showed a significant increase in these marks around their transcription start site (TSS) regions (±2000 bp) compared with 13 genes that showed a decrease (Table S1), a difference that likely reflects secondary effects and/or compensatory mechanisms. H3K27me3 is a broad chromatin mark localized not only at TSS but also spread over gene bodies. We also looked at the number of genes with altered H3K27me3 at their coding regions (including TSS). There were 484 genes with increased and 156 genes with decreased H3K27me3 (Figure 1E and Table S1). Strikingly, loss of BAF complexes in late corticogenesis resulted in a concurrent increase in activatory H3K4me2 marks in the E17.5 pallium (Figure 1D), with 1,265 genes showing a significant increase in this mark around their TSSs (Figure 1E, Table S1). Only 112 genes showed decreased H3K4me2, which again may represent some secondary effects. Importantly, genes affected by increased H3K27me3 and H3K4me2 were largely distinct (Figure 1E).

We also performed gene expression profiling of the *dcKO* pallium at E17.5 (Figure 1F). In contrast to the globally reduced gene expression in the *dcKO*_*Emx1*-Cre pallium at E12.5 (Narayanan et al., 2015), at E17.5, we found nearly equal number of downregulated and upregulated genes in the *dcKO* pallium (Figure 1F; Table S2).

Collectively, these data indicate that loss of BAF complexes during late corticogenesis induces an increase in activatory H3K4me2 and repressive H3K27me3 marks at distinct sets of genes, thereby pointing to possible dual functions of BAF complexes as both activators and repressors in late cortical neurogenesis.

### Conditional Inactivation of BAF Complexes during Late Cortical Development Impairs Neurogenesis of Upper Cortical Layer Neurons and the Hippocampus

**Figure 2:**
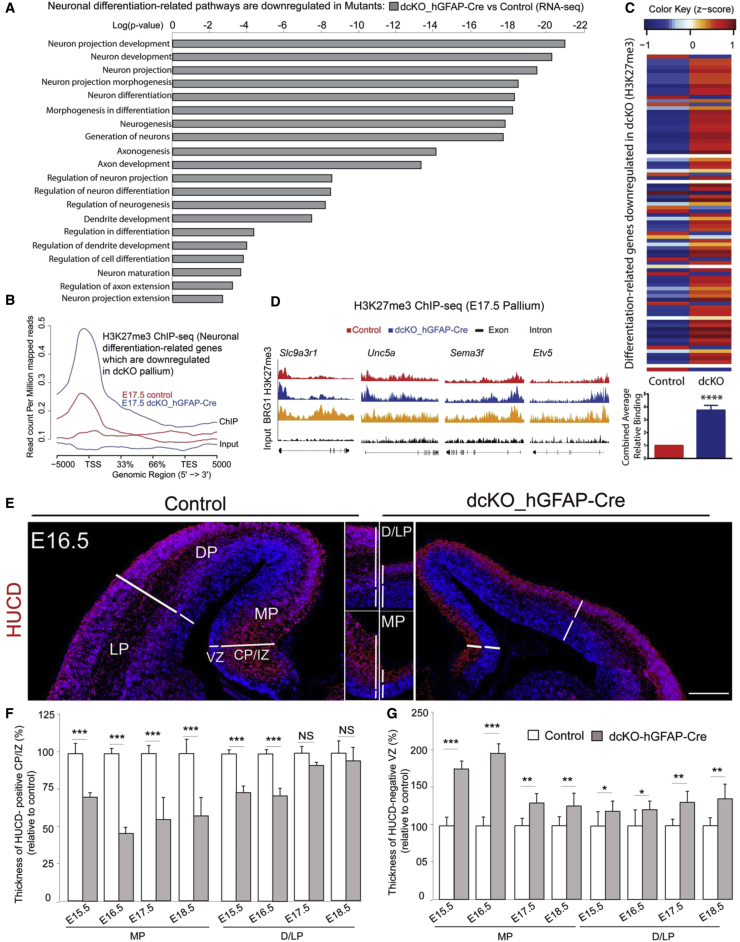
H3K27me3-Linked Silencing of Neuronal Differentiation-Related Genes in BAF Complex-Deleted Pallium in Late Stages (A) Neuronal differentiation-related genes are downregulated in the dcKO pallium at E17.5. (B) General H3K27me3 profile plot of neuronal differentiation-related genes that are downregulated in dcKO pallium. (C) Upper panel: heatmap depicting the changes in H3K27me3 levels at neural differentiation-related genes that are downregulated in dcKO pallium at E17.5 individually. Lower panel: average relative H3K27me3 binding levels on those genes combined. (D) Integrated genome browser views of H3K27me3 and BRG1 (GEO: GSE37151; Attanasio et al., 2014) binding along representative neural differentiation-related genes downregulated in dcKO pallium. (E–G) IF (E) and quantitative (F and G) analyses indicate that the loss of BAF155 and BAF170 leads to a diminished thickness of the HUCD^+^ cortical plate (CP) and intermediate zone (IZ) (F), and expanded thickness of the HUCD^−^ VZ (G) in the entire pallium at E15.5–E18.5. Values are presented as means ± SEMs (^∗^p < 0.05; ^∗∗^p < 0.01; ^∗∗∗^p < 0.005; ^∗∗∗∗^p < 0.0001). Experimental replicates (n) = 6 (F and G). Abbreviations: VZ, ventricular zone; CP, cortical plate; IZ, intermediate zone; MP, medial pallium; DP, dorsal pallium; LP, lateral pallium. Scale bar represents 100 μm (E).

We selected the downregulated genes in the E17.5 dcKO pallium in RNA sequencing (RNA-seq) and subjected them to functional category analysis. They are enriched in neuronal differentiation-related categories (Figure 2A, Table S2) and showed an overall increase in H3K27me3 mark (Figure 2B). Most of the differentiation-related genes that were significantly downregulated in *dcKO* mice (Table S3) showed an increase in H3K27me3. For some selected candidates, we also confirmed their downregulation and increased H3K27me3 by qPCR and ChIP-qPCR respectively (Figures S2A and S2B).

Next, we asked if these genes with decreased expression and increased H3K27me3 are directly bound by the BAF complexes. We made use of a previously published ChIP-seq dataset (GEO: GSE37151) for BRG1 in the developing mouse forebrain (Attanasio et al., 2014). Strikingly, the majority of genes that showed increased H3K27me3 in dcKO cortices were also bound by BRG1 (Figure S2C), with sites of increased H3K27me3 co-localizing with BRG1 binding sites (Figure 2D).

We further confirmed these observations in a reverse approach, in which we first selected the genes with increased H3K27me3 in E17.5 dcKO (Figure S2D) and subjected them to functional category analysis. Again, they also mostly fell under neuronal differentiation-related categories (Figure S2E). We then examined their expression in our RNA-seq analysis. As expected, most of them were downregulated in dcKO embryos.

Because the *hGFAP* promoter is active early in the MP (from E13.5) and later in the DP and LP (from E14.5) (Figures S1B and S1C), we compared neuronal differentiation between controls and dcKO mutants, in both the MP and at the area between the DP and LP (D/LP). Neurogenesis in late (E15.5–E17.5) development of the pallium in dcKO mutants was decreased, as shown by a decrease in the thickness of the cortical plate (CP) and intermediate zone (IZ), marked by the expression of the pan-neuronal markers HUCD, TUBB3, and NEUN in both the cortex (D/LP) and hippocampus (MP) (Figures 2E and 2F). Consistent with this, immunofluorescence (IF) analyses of neuronal subtype markers indicated that loss of BAF155 and BAF170 led to a significant decrease in the number of late-born SATB2^+^ or BRN2^+^ neurons, but not early-born TBR1^+^ neurons, in the DP and LP (Figures S3A–S3D).

**Figure 3:**
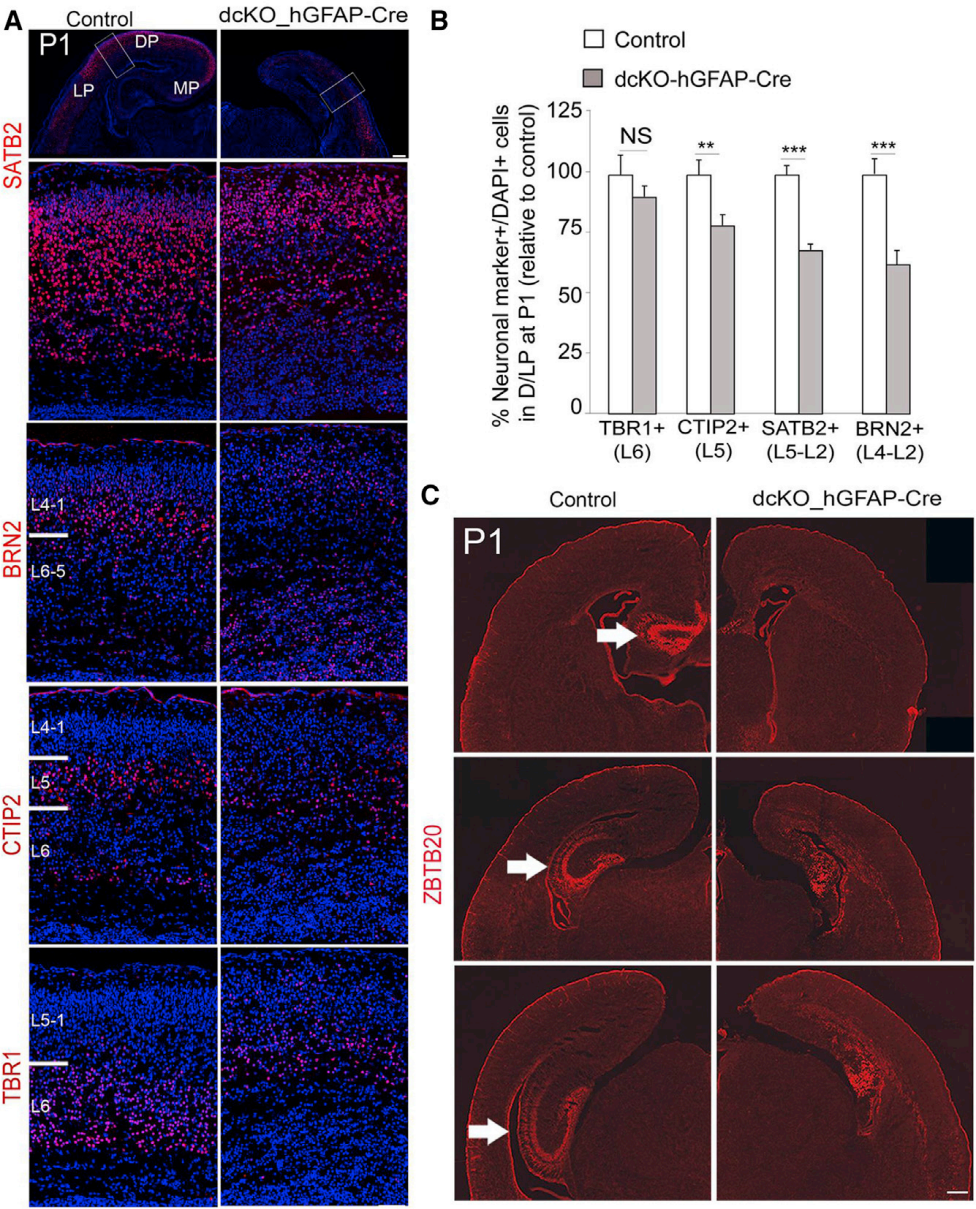
BAF Complexes Are Required for the Formation of Cortical Upper Layers and the Hippocampus (A and B) IF (A) and statistical (B) analyses of cortical phenotypes at postnatal stage 1 (P1) in a comparable dorsal/lateral area, immunostained for the indicated neuronal layer markers. NS, not significant. (C) IF analysis of Ztbt20 revealed that the hippocampus is underdeveloped in mutants (denoted by arrow). Values are presented as means ± SEMs (^∗∗^p < 0.01; ^∗∗∗^p < 0.005). Experimental replicates (n) = 4 (B). Abbreviations: MP, medial pallium; DP, dorsal pallium; LP, lateral pallium; L, layer. Scale bars represent 100 μm (A) and 100 μm (C).

To study neurogenesis specifically in the MP, we performed IF on sections from E15.5–E17.5 control and dcKO embryonic brains using the antibody ZBTB20 (Figure S3E), which outlines the hippocampal anlage as early as E14.5 and is confined postnatally to hippocampal cornu ammonis (CA1–CA3) regions. ZBTB20 staining revealed remnants of the hippocampus proper (Figure 3C) in mutants compared with controls at all examined stages, E14.5–E17.5 (Figures S3E and S3G). Indeed, three-dimensional (3D) reconstruction of ZBTB20 expression also revealed a substantial reduction in the volume of the developing hippocampus in dcKO embryos at E15.5 (Figure S3H and Video S1). Consistently, immunostaining of the dentate gyrus (DG) with its specific marker PROX1 indicated agenesis of this hippocampal domain (Figures S3F and S3I). In the DP/LP of mutants, whereas the generation of lower layer (TBR1^+^/L6, and CTIP2^+^/L5) neurons was only mildly decreased, the number of late-born SATB2^+^, and BRN2^+^ L4–L2 neurons was strongly diminished (Figures 3A and 3B). In further support, we found that BAF complexes control expression of sets of gene exerting important roles in generation of cortical layers and hippocampal development (Table S4).

To gain additional evidence about how the defect in neuronal differentiation is caused by increased level of H3K27me3, we used GSK-J4, a potent selective H3K27 demethylase (JMJD3 and UTX) inhibitor (Kruidenier et al., 2012). The elevated level of H3K27me3 by GSK-J4 administration significantly decreased the number of late-born SATB2^+^ and CUX1^+^ neurons (Figures S4A–S4D), as observed in dcKO pallium with enhanced level of H3K27me3.

Together, these findings suggest that deletion of BAF complexes in late NSCs leads to H3K27me3-linked silencing of neuronal differentiation genes and results in diminished late cortical and hippocampal neurogenesis.

### The NSC Pool Is Increased at Late Development Stages in the dcKO Pallium

**Figure 4:**
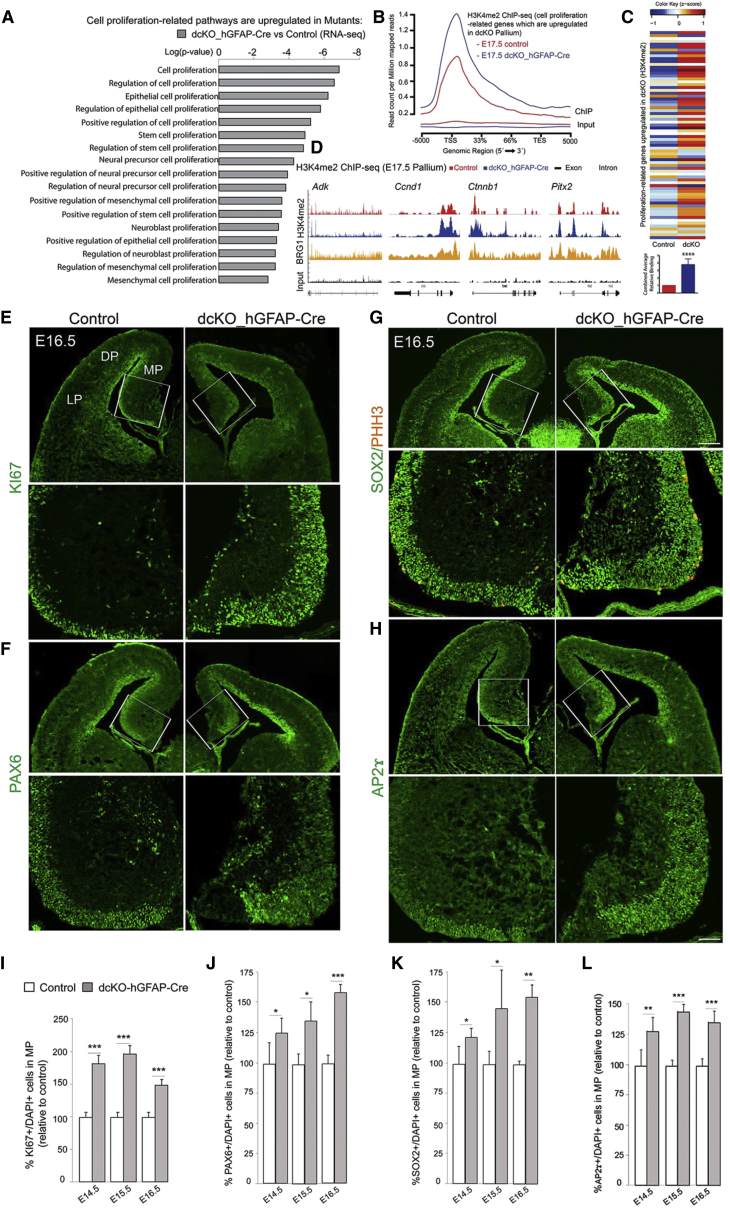
Loss of BAF155 and BAF170 Causes H3K4me2-Linked Upregulation of Genes Involved in the Mitotic Cell Cycle and Proliferation in Late Cortical Development (A) Proliferation- and cell-cycle-related genes are upregulated in the dcKO pallium at E17.5. (B) General H3K4me2 profile plot at proliferation-related genes that are upregulated in dcKO pallium. (C) Upper panel: heatmap depicting the changes in H3K4me2 levels at proliferation-related genes that are upregulated in dcKO pallium at E17.5. Lower panel: average relative H3K4me2 binding levels on those genes combined. (D) Integrated genome browser views of H3K4me2 and Brg1 binding (GEO: GSE37151) (Attanasio et al., 2014) along representative proliferation-related genes upregulated in dcKO pallium. (E–H) Representative images showing IF analyses of coronal sections of control and dcKO pallium at E16.5 using antibodies that specifically label the indicated NSC markers. Lower panels: higher-magnification images of areas indicated by white boxes. Note that a similar image of triple channels for PAX6/TBR2/CASP3 is shown in Figure S6H. (I–L) Quantitative analyses indicated increased numbers of NSCs in the MP of dcKO mutants at the indicated stages. Values are presented as means ± SEMs (^∗^p < 0.05; ^∗∗^p < 0.01; ^∗∗∗^p < 0.005; ^∗∗∗∗^p < 0.0001). Experimental replicates (n) = 6 (I and L), 4 (J and K). Abbreviations: TSS, transcription start site; TES, transcription end site; MP, medial pallium; DP, dorsal pallium; LP, lateral pallium. Scale bars represent 100 μm (G) and 50 μm (H).

Our previous data indicated that the loss of BAF complexes leads to large-scale downregulation of gene expression in early cortical development (Narayanan et al., 2015, Nguyen et al., 2016). Intriguingly, the late elimination of BAF complex function also led to upregulation of a substantial number of genes. In order to assess the role of the genes upregulated in dcKO embryos, we applied the aforementioned strategy. Functionally, they mainly converged into cell proliferation-related categories (Figure 4A, Table S2). Moreover, these genes also showed an overall increase in H3K4me2 in the dcKO pallium (Figure 4B, Table S3).

Next, we assessed the changes in H3K4me2 levels at their individual TSS regions. As expected, most of them had an increase in this activatory mark with the overall trend being highly significant (Figure 4C) and they mostly converged into cell cycle-related groups (Figures S2D and S2E). The sites of increased H3K4me2 also substantially overlapped with BAF complex (BRG1) binding (Figures 4D and S2C). Selected candidates were confirmed by qPCR and ChIP-qPCR (Figures S2A and S2B). Because the expression of genes encoding H3 demethylases LSD1/kdm1a, UTX/KDM6A, and JMJD3/KDM6B was unaltered in dcKO cortex in our RNA-seq experiment (Table S2), it is possible that BAF complexes control the methylation of H3K4 and H3K27 through mechanisms other than activating or inhibiting the expression of genes coding for these H3 demethylases. Our earlier study indicated that BAF complexes potentiate the H3K27 demethylase activity of UTX/KDM6A and JMJD3/KDM6B (Narayanan et al., 2015), which encouraged us to investigate whether endogenous BAF155 and BAF170 are required for full H3K4 demethylase activity of LSD1/KDM1A. We therefore performed the histone demethylase KDM1/LSD1 activity quantification assay (see Experimental Procedures). The results revealed that significantly less H3K4 is demethylated in BAF155/BAF170-ablated NSCs compared with control counterparts (Figure S2G).

IF analysis of the expression of HUCD, TUBB3, and NEUN indicated an enlargement of the VZ in the dcKO pallium, more strongly in MP than in D/LP (Figures 2E and 2G). Reconstruction analyses showed that the volume of the hippocampal neuroepithelium, as revealed by PAX6 expression, is larger in the mutant MP (Figures S5A and S5B; Video S1). These data suggest increased pools of progenitors in proliferative zones of the dcKO pallium. Indeed, more KI67^+^ mitotically active cells were found in mutants than in controls (Figures 4E, 4I, and S5C). We then examined pools of RGs and intermediate progenitors (IPs) (Figures 4F–4H, 4J–4L, and S5D–S5G). Similar to the increased number of KI67^+^ mitotic cells, the number of PAX6^+^, SOX2^+^, and AP2γ^+^ NSCs in the VZ gradually increased from E14.5 in the mutant MP (Figures 4F–4H). Notably, the effect was more profound in the NSC pool in the MP than in the D/LP (Figures S5C–S5F). This possibly relates to the spatiotemporal *hGFAP*-Cre activity, exerting early activity in the MP (Figures S1B and S1C). In contrast to the increased number of RGs, the number of TBR2^+^ IPs was decreased (Figures S5D and S5G), indicating disruption of neuronal differentiation in the mutant pallium.

To substantiate the effect of the H3K4me2 level on the cortical NSC pool, we examined an increased H3K4me2 by using (±)-trans-2-phenylcyclopropylamine hydrochloride (2-PCPA), a specific inhibitor of LSD1 histone demethylase. 2-PCPA has been shown to increase H3K4me2 in mouse brain (Sun et al., 2010). The treatment of 2-PCPA also led to an increased pool of PAX6^+^, SOX2^+^ NSCs in developing cortex (Figures S4E–S4G).

Together, these findings suggest that, in the absence of BAF155/BAF170, at late corticogenesis (E14.5–E17.5), NSCs in the VZ are kept in the proliferative phase rather than differentiating into IPs and/or neurons.

### RGs Acquire an NE-like Identity in the BAF155/BAF170-Deficient Pallium

**Figure 5:**
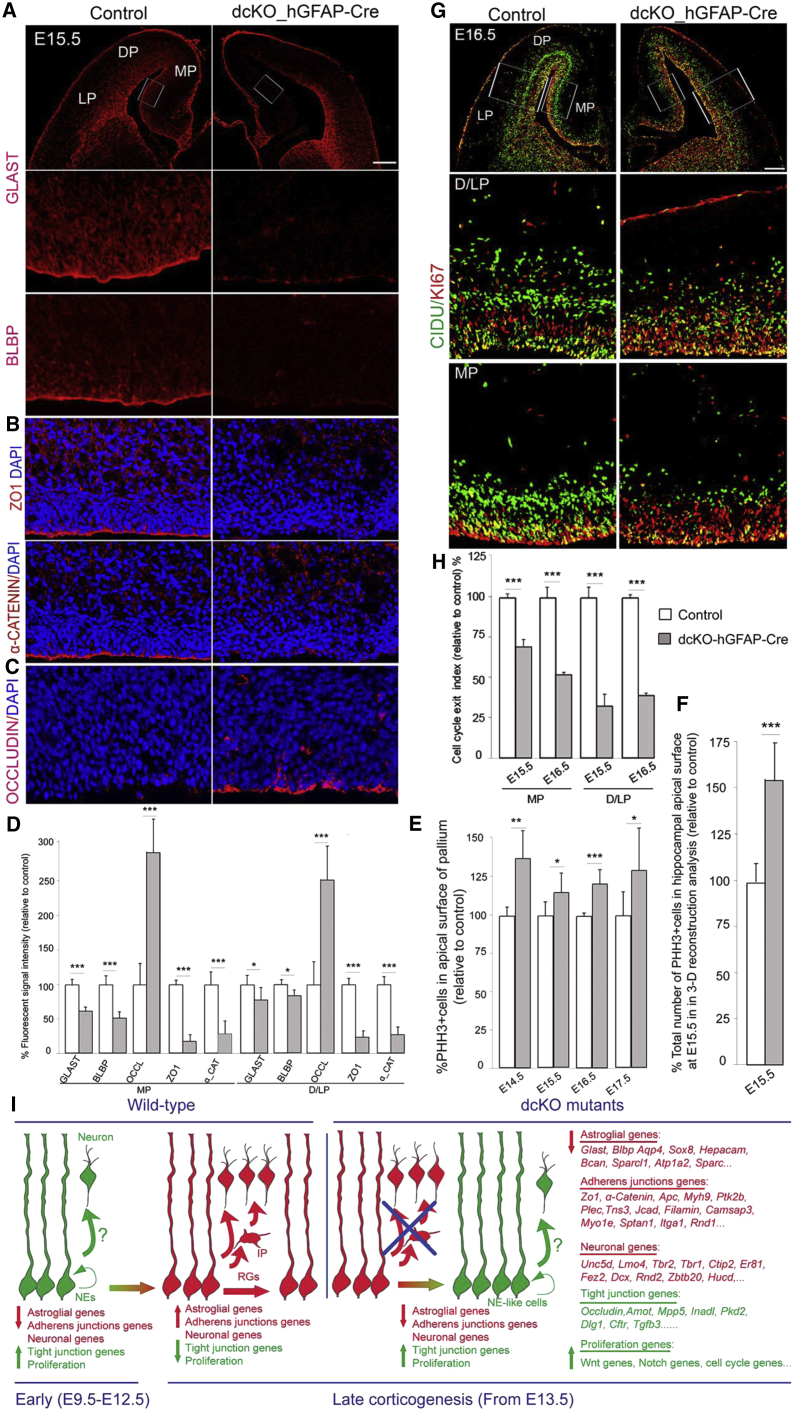
NE-like Cells in the BAF-Complex-Deleted Pallium in Late Development Retain Their Highly Proliferative Competence (A–C) Immunostaining of the control and dcKO pallium sections at E15.5 for indicated markers revealed an altered cell identity from GLAST^high+^/BLBP^high+^/ZO^high+^/α-Catenin^high+^/OCCLUDIN^−^ RGs in controls to GLAST^low+^/BLBP^low+^/ZO^low+^/α-Catenin^low+^/OCCLUDIN^high+^ NEs in dcKO MP. (D) Quantification and statistical analysis of (A)–(C) are shown. (E and F) Quantitative analyses showing that the loss of BAF155 and BAF170 leads to an increase in mitotic PHH3^+^ RGs in the pallium at E14.5–E16.5 (E). Note that quantification of PHH3^+^ cells (F) was done in the entire developing hippocampus (PAX6^+^/ZBTB20^+^) at E15.5 using 3D reconstruction (see also Figures S5A and S5B, Video S1). (G) Images showing double IF at E16.5 for CIDU, and KI67 in control and dcKO mutants. (H) Quantitative analyses showing a significantly lower exit index (number of CIDU^+^/KI67^-^ cells per total number of CIDU^+^ cells) in mutants in D/LP and MP areas than in controls. (I) Schema illustrating that a higher proportion of RG progenitors in the late-stage (from E13.5) dcKO pallium acquire NE-like identity (i.e., downregulated expression of astroglial, adherens junction, differentiation genes and upregulated expression of tight junction, proliferation genes). Values are presented as means ± SEMs (^∗^p < 0.05; ^∗∗^p < 0.01; ^∗∗∗^p < 0.005). Experimental replicates (n) = 6 (D and E), 4 (F and H). Abbreviations: NE, neuroepithelial cell; RG, ventricular radial glial progenitors, Hi, hippocampus; Cx, cortex; MP, medial pallium; DP, dorsal pallium; LP, lateral pallium. Scale bars represent 100 μm (A and G).

The appearance of RGs in the pallium is marked by initiation of the expression of the astrocytic differentiation markers GLAST and BLBP at E12.5 (Hartfuss et al., 2001, Sahara and O'Leary, 2009). At E13.5 and E14.5, expression level of GLAST and BLBP is comparable between control and mutants (Figures S5H–S5L). Following IF analysis at later stages (E15.5–E16.5) we observed that, despite the increased number of PAX6^+^/SOX2^+^/AP2γ^+^ NSCs (Figures 4F and 4G), immunopositive signals for GLAST and BLBP were diminished in the DP/LP and largely undetectable in the MP in dcKO mutants (Figures 5A and 5D).

Another hallmark for NE-RG cell transition is the replacement of tight junctional complexes (NE trait) with adherens junctions (RGs trait) (Aaku-Saraste et al., 1996, Sahara and O'Leary, 2009). Notably, we found that, during late corticogenesis, many genes encoding for tight junction proteins (e.g., *Amot*, *Mpp5*, *Occludin*, *Inadl*, *Pkd2*, *Dlg1*, *Cftr*, *Tgfb3*) were significantly upregulated and also those involved in adherens junction proteins (e.g., *Tns3*, *Plec*, *Ptk2b*, *Zo1*, *α-Catenin*, *Kiaa1462/Jcad*, *filamin*, *Camsap3*, *Apc*, *Myh9*, *Myo1e*, *Sptan1*, *Itga1*, *Rnd1*) were downregulated in the dcKO pallium (Tables S2 and S4). Additionally, we examined VZ expression of OCCLUDIN (a tight junction marker) and ZO1, α-CATENIN (adherens junction markers) localized at the apical surface. OCCLUDIN is normally downregulated in NEs as they differentiate into RGs (Aaku-Saraste et al., 1996, Sahara and O'Leary, 2009). At E13.5–E16.5, expression of OCCLUDIN at the apical surface of the VZ in the control pallium was undetectable, whereas its expression was strongly upregulated in the dcKO pallium at E15.5–E16.5 (Figures 5C, 5D, and S5I). The expression of adherens junction markers ZO1, and α-CATENIN at the apical surface of RGs was not affected at E13.5–E14.5 (Figures S5J–S5L) but was largely absent at E16.5–E17.5 in dcKO cortex (Figures 5B and 5D).

BAF complexes seem not only to block NE fate in late pallium development but also control the differentiation from NEs to RGs, as shown by downregulated expression of the RG markers BLBP and GLAST together with upregulated expression of the NE marker OCCLUDIN in the dcKO_*Emx1*-Cre cortex at E13.5 (Figures S5M–S5O). Thus, our data revealed that the downregulation of the expression of astroglial and adherens junction markers is correlated with upregulation of tight junction markers in late cortical development of dcKO mutants. These complementary datasets indicate that deletion of BAF complexes during late development of the pallium dedifferentiates RGs to NE-like cells.

### Change in Spindle Orientation and Increased Proliferative Capacity of NSCs in the BAF155/BAF170-Deficient Pallium

To assess the implications of the retention of an NE-like identity in the mutant cortex, we first found out whether the dedifferentiation from RGs to NE-like cells was consequent to or caused altered spindles orientation. We stained E15.5–E16.5 sections from control and dcKO pallium using antibodies against PVIM and PHH3 to mark mitotic cells and chromatin, respectively (Figure S5A). The division angles of apical RGs were quantified and categorized based on cleavage angle: vertical (60°–90°), oblique (30°–60°), and horizontal (0°–30°). Notably, more progenitors with vertical cleavage were detected in the mutant pallium than in controls (Figures S5A and S5B), suggesting that the loss of BAF complexes in late pallial development induces proliferative symmetric divisions, which mainly generate NEs and RGs.

The increased number of PAX6^+^/SOX2^+^ NSCs suggested that RGs were kept in the cell cycle to promote their proliferation, instead of exiting to become neurons. To ascertain whether loss of functional BAF155 and BAF170 leads to altered cell proliferation, we labeled M-phase cells by immunostaining with an anti-PHH3 antibody. Quantitative comparisons of immunostained medial brain sections of the E14.5–E17.5 pallium (Figures 4A and 5D) and 3D reconstruction analyses of the entire hippocampus at E15.5 (Figure 5E) indicated that the loss of BAF155 and BAF170 resulted in an increased number of PHH3^+^ cells in the pallium (Figures 5D and 5E).

To better characterize BAF155/BAF170 loss-of-function effects on neuronal differentiation, we next used a thymidine analog (CIdU) injection paradigm (24-hr CIDU pulse labeling) to establish a quantitative *in vivo* cell cycle exit index in the developing MP and D/LP (Figures 5G and 5H). We also performed double-labeling IF using antibodies against CIDU to label both cycling progenitors and those that recently exited the cell cycle, and KI67 for proliferating progenitors in all cell cycle phases. Statistical analyses revealed a significantly lower cell cycle exit index in dcKO mutants compared with controls (Figures 5G and 5H). To examine whether BAF155/BAF170-deficient NSCs undergo several proliferative rounds, we again detected the sequential incorporation of different thymidine analogues (CIdU, IdU) into cortical NSCs (Figure S6C). Given that cell cycle length of cortical progenitors between E14.5 and E16.5 is about 15–18 hr per cycle (Takahashi et al., 1995), pregnant mice were injected with CIdU (at E14.5) and IdU (at E15.5). Tissue was collected at E16.5 and processed for IHC analysis with antibodies against CIDU, IDU, and KI67 (Figure S6D). The cortical cells between 1 and 3 successive rounds of cell division were labeled as follows: (1) cells from the first and the second cell divisions as well as their progenies are marked with CIDU (in green) and with IDU (in violet) respectively; (2) cells in third mitotic cell cycle will be labeled by KI67 (red). Our statistical analysis (Figure S6E) indicated that, between E14.5 and E16.5, many cortical progenitors exit from the first (CIDU^+^/IDU^−^/KI67^-^: 6% ± 0.83% in control and 0.7% ± 0.38% in dcKO, green part of chart) and second (CIDU^+^/IDU^+^/KI67^−^: 73% ± 2.52% in control and 31.82% ± 3.30% in dcKO, violet part of chart) cell cycles in control cortex, whereas a large fraction of NSCs further enters third (CIDU^+^/IDU^+^/KI67^+^: 20.16% ± 1.94% in control and 67.47% ± 9.70% in dcKO, red part of chart) cell cycle in dcKO cortex.

We also examined apoptosis at different stages from E14.5 to E18.5 in the MP by performing IF for CASP3 (Figures S6F–S6K). Compared with controls, significantly higher numbers of dying cells were found in the MP of dcKO pallium (Figures S6F–S6J). Particularly, most apoptotic cells in mutants were PAX6^+^ RGs, while apoptotic TBR2^+^ IPs were detected to a lesser extent. Apoptotic HUCD^+^ neurons were rarely detected (Figures S6F–S6I and S6K), and this paralleled our previously observed apoptotic effect of selective loss of BAF complex in post-mitotic neurons. The latter effect is further supported by the observation that selective elimination of BAF155 and BAF170 in post-mitotic neurons in dcKO_*Nex*-Cre had no effect on the populations of CTIP2^+^/ZBTB20^+^ neurons, PAX6^+^/SOX2^+^ NSCs (Figures S6L–S6N) or CASP3^+^ apoptotic cells (Narayanan et al., 2015).

Collectively, these findings indicate that the deletion of BAF complexes results in H3K4me2-linked activation of proliferation- and cell-cycle-associated genes. This resulted in three main morphogenetic defects of the dcKO pallium: (1) an expanded pool of NSCs, (2) diminished neurogenesis in late corticogenesis, and (3) malformed late-formed structures such as upper cortical layers and the hippocampus.

### Elimination of BAF155 and BAF170 De-represses Wnt Signaling in Late Corticogenesis

**Figure 6:**
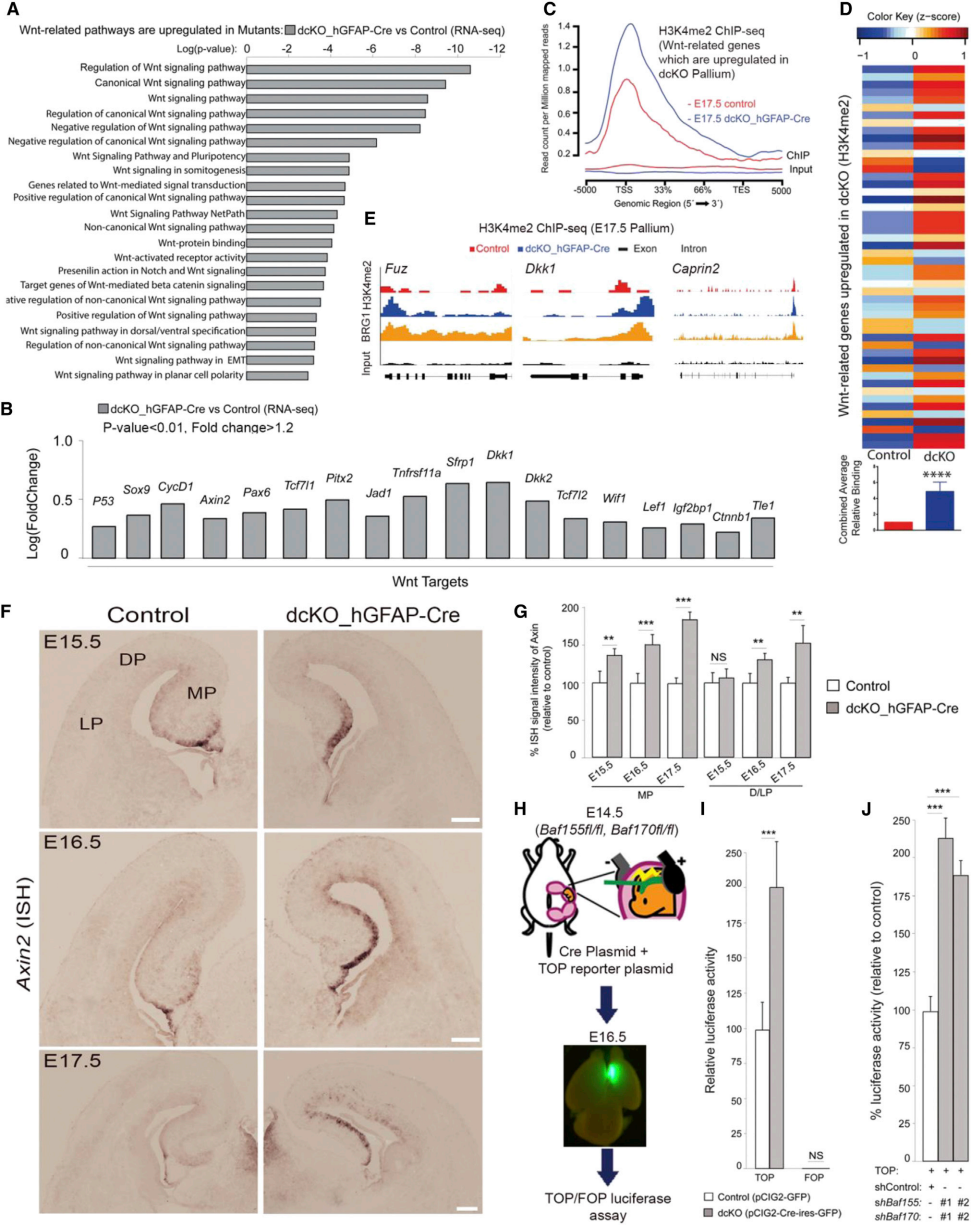
BAF Complexes Suppress Wnt Signaling Activity (A) Wnt-related genes are upregulated in the dcKO pallium at E17.5. (B) Wnt target genes upregulated in the dcKO pallium are shown. (C) General H3K4me2 profile plot of Wnt-related genes that are upregulated in dcKO pallium. (D) Upper panel: heatmap depicting the changes in H3K4me2 levels of Wnt-related genes that are upregulated in dcKO pallium at E17.5. Lower panel: average relative H3K4me2 binding levels on those genes combined. (E) Integrated genome browser views of H3K4me2 and BRG1 binding (GEO: GSE37151) (Attanasio et al., 2014) along representative Wnt-related genes upregulated in dcKO pallium. (F and G) ISH (F) and quantitative (G) analyses comparing the expression of the Wnt target *Axin2* in the control and dcKO pallium at E15.5–E17.5. (H–J) *In vivo* (H and I) and *in vitro* (J) luciferase assay indicating higher Wnt signaling activity in BAF155/BAF170-depleted pallial cells (I) and in Neuro2A cells (J) compared with control cells. Values are presented as means ± SEMs (^∗∗^p < 0.01; ^∗∗∗^p < 0.005; ^∗∗∗∗^p < 0.0001). Experimental replicates (n) = 6 (G and J), 4 (I). Abbreviations: TSS, transcription start site; TES, transcription end site; MP, medial pallium; DP, dorsal pallium; LP, lateral pallium. Scale bars represent 100 μm (F).

Previous work has suggested that enhanced Wnt signaling promotes cortical NSC proliferation (Chenn and Walsh, 2002, Machon et al., 2007). We found that, during late corticogenesis, a considerable number of genes involved in Wnt signaling, including many Wnt target genes, were significantly upregulated in the dcKO pallium (Figures 6A, 6B, and S7A, Table S2). These genes showed an overall (Figures 6C and 6D) increase in H3K4me2 levels. Moreover, their TSS regions, where increased H3K4me2 is observed in dcKO embryos, also coincide with BRG1 binding sites (Figure 6E).

To provide additional support, we also performed *in situ* hybridization (ISH) analysis of the expression of *Axin2*, a direct target of Wnt/β-catenin activity. This analysis showed that, unlike controls, in which *Axin2* mRNA staining was faint and confined mostly to the MP at E15.5–E17.5, the BAF complex-deficient pallium exhibited diffused *Axin2* staining in the MP VZ at E15.5 and throughout the pallium VZ at E16.5–E17.5 (Figures 6F and 6G).

**Figure 7:**
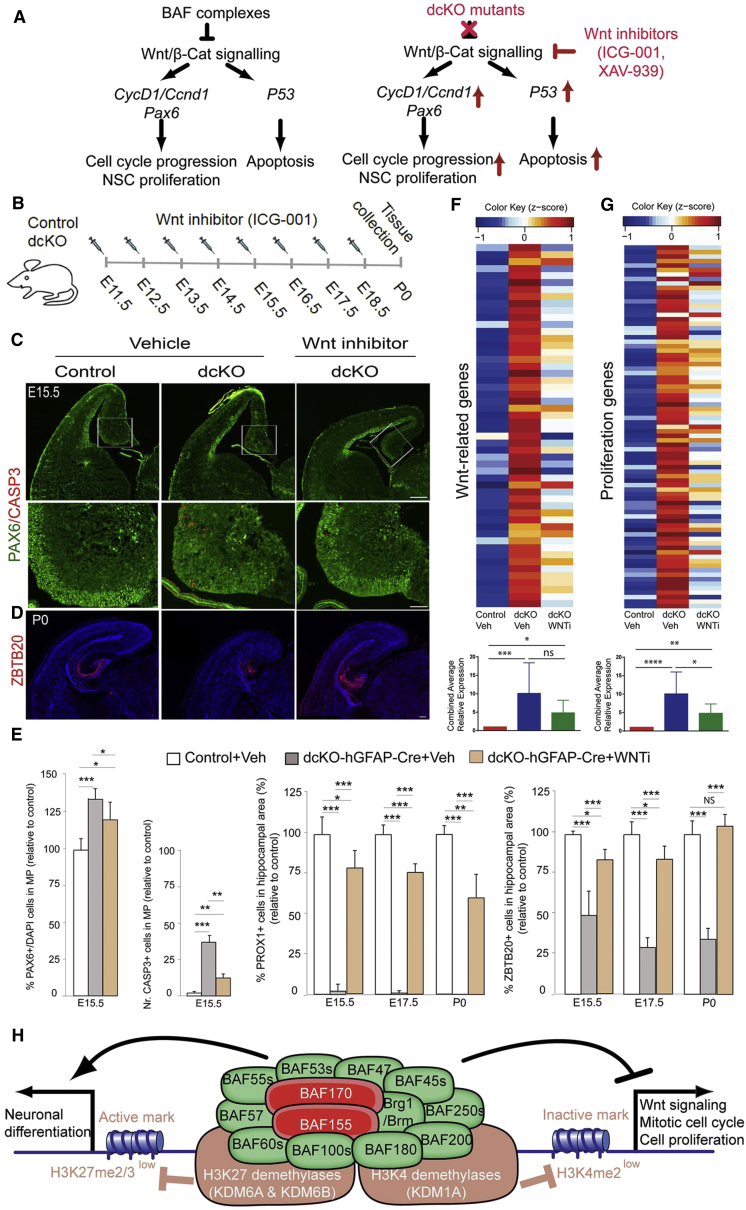
BAF Complexes Control Hippocampal Development by Suppressing Wnt Signaling Activity (A) Schematic model of the molecular cascades underlying late stages of pallium development in WT and dcKO pallium. (B) Rescue experimental paradigm with the Wnt inhibitor (WNTi, ICG-001). (C–E) IF (C and D) and quantitative (E) analyses of dcKO mutants at the indicated stages, showing the effects of treatment with ICG-001 on pools of PAX6^+^ NSCs (C and E), CASP3^+^ apoptotic cells (C and E), and ZBTB20^+^ (D and E) and PROX1^+^ neurons (E) in the developing hippocampus. (F and G) Expression of Wnt (F) and Proliferation (G)-related genes in control, vehicle (Veh), WNTi-treated pallium. (H) A proposed model showing how loss of BAF155 and BAF170 in dcKO mutants controls epigenetic and neural gene expression programs in proliferation and neuronal differentiation of the pallium in late developmental stages. Values are presented as means ± SEMs (^∗^p < 0.05; ^∗∗^p < 0.01, ^∗∗∗^p < 0.005; ^∗∗∗∗^p < 0.0001). Experimental replicates (n) = 4 (E, F, and G). Scale bars represent 100 μm (C, D, and F) and 50 μm (C).

To further address the capacity of BAF complexes to regulate Wnt signaling, we performed *in vivo* reporter assays by electroporating a luciferase promoter construct TOP, containing β-catenin/TCF binding sites and a mutated form, FOP, as negative control into the embryonic brain. To eliminate BAF function, we electroporated TOP-/FOP-FLASH reporter plasmids plus a Cre-expressing plasmid into the E14.5 MP of *Baf155*^*fl/fl*^*:Baf170*^*fl/fl*^ embryos. We then examined isolated tissue samples from the MP using the TOP/FOP luciferase assay. These analyses indicated that BAF complex knockout in the pallium significantly enhanced TOP-, but not FOP-reporter activity (Figure 6H and 6I). Similarly, dual silencing of *Baf155* and *Baf170* markedly increased Wnt signaling activity in Neuro2A cells *in vitro* (Figure 6J), suggesting that BAF complex deficiency indeed increased the transcriptional activity of the Wnt target genes that control NSC proliferation. Such candidate genes (e.g., *Pax6*, *Ap2γ*, and *Cyclin D1*) are critical for the timely progression of the cell cycle (Figure 7A).

Next, we directly determined whether BAF complex in the MP regulates hippocampal development via suppression of Wnt signaling. To this end, we used ICG-001, a Wnt signaling inhibitor, to perform rescue experiments (Figures 7A and 7B). Starting from E11.5, pregnant mice were intraperitoneally injected daily with an ICG-001 solution, and brain samples were collected at E15.5, E17.5, and postnatal stage 0 (P0) (Figure 7B). ICG-001 treatment of dcKO mutants resulted in the reversal of NE-like cell characteristics (BLBP^low^/GLAST^low^/OCCLUDIN^high^) to RG features (BLBP^high^/GLAST^high^/OCCLUDIN^low^) that typify the WT pallium (Figures S7B–S7E). Furthermore, IF analyses at E15.5 with PAX6 and CASP3 antibodies revealed that the Wnt inhibition decreased the number of PAX6^+^ NSCs and CASP3^+^ apoptotic cells (Figures 7C and 7E) in dcKO mutants. Concurrently, ICG-001 administration in dcKO mutants caused a near-WT increase in the number of PROX1^+^ DGs and ZBTB20^+^ hippocampal neurons in MP (Figures 7D and 7E) and also in the number of CTIP2^+^, SATB2^+^, and CUX1^+^ cortical neurons in L/DP (Figures S7F–S7H). Strikingly, treatment with the Wnt inhibitor almost completely rescued the aberrant hippocampal morphology in mutants (Figures 7D and 7E). To consolidate this claim, pregnant mice were treated with XAV-939, a substance with similar effect as ICG-001 (Mutch et al., 2010). As expected, XAV-939 treatment reproduced the Wnt inhibition-dependent rescue of cortical anomalies in dcKO mutants (Figures S7I–S7O).

Finally, we compared the transcriptome of cortices from control and dcKO embryos, which were treated with either Veh or Wnt inhibitor (Table S5, Figures 7F and 7G). Treatment with Wnt inhibitor decreases the expression of proliferation- and Wnt-related genes that are upregulated in dcKO embryos (Figures 7F and 7G).

Taken together, these results demonstrate that loss of BAF complexes during late cortical neurogenesis leads to aberrant enhancement of Wnt signaling activity and causes increased NSC proliferation-related defects similar to those observed after Wnt/β-catenin overexpression (Chenn and Walsh, 2002, Machon et al., 2007). These findings demonstrate that BAF complexes are required for proper hippocampal development through appropriate suppression of Wnt signaling in late developmental stages of the pallium.

## Discussion

In this study, we present evidence for the involvement of chromatin-remodeling BAF complexes in the regulation of global gene expression and epigenetic programs during late cortical neurogenesis. We showed that specific interactions of BAF155/BAF170 subunits with H3K27 and H3K4 demethylases possibly potentiate their activity during corticogenesis. During late development, loss of H3K27me3 and H3K4me2 marks on regulatory regions of distinct sets of genes potentiates disinhibition of transcription of RG- and neuronal differentiation-related genes, and suppresses NE-, Wnt signaling-, cell cycle-, and proliferation-related genes, respectively (Figure 5I). Thus, BAF complexes act both as activators and as repressors to regulate global epigenetic and gene expression programs during late corticogenesis and hippocampus development.

### BAF155/BAF170-Dependent Maintenance of RG Cell Fate during Late Cortical Neurogenesis

Cortical neurogenesis comprises three main phases: (1) an expansion phase, characterized by symmetric division of NEs and expansion of the proliferative cell population; (2) a transition period during which NEs differentiate into RGs via asymmetric divisions to generate neurons as well as basal progenitors; and (3) a terminal phase during which progenitors undergo a terminal symmetric division to generate neurons and become quiescent (Dehay and Kennedy, 2007, Gotz and Huttner, 2005, Kriegstein and Alvarez-Buylla, 2009, Martynoga et al., 2012).

A few molecular factors are known to regulate NE to RG transition and RG differentiation (Dehay and Kennedy, 2007, Gotz and Huttner, 2005, Kriegstein and Alvarez-Buylla, 2009, Martynoga et al., 2012). For example, ablation of *Fgf10* delayed RG differentiation during early corticogenesis, whereas NE fate seemed unaffected (Sahara and O'Leary, 2009). It is thus conceivable that, at later stages, other mechanisms may be required in limiting NSC fate to allow neuronal differentiation.

We showed that elimination of the BAF complex, by deleting the two scaffolding subunits BAF155 and BAF170 in cortex from E14.5 onward, results in a loss of RG fate hallmarks (diminished expression of astroglial and adherens junction markers), accompanied by gain of NE features (activation of tight junction and proliferation genes) (Figure 5H). Phenotypically, in the *dcKO* pallium, we found an overactive progenitor proliferation through symmetric divisions (a feature of NEs), instead of the typical predominant late-stage asymmetric division to produce one RG and a neuron or an IP. This leads to overproduction of NSCs at the expense of their derivatives (IPs and upper-layer neurons), and instigating the decreased radial cortical thickness and hypoplasia of the hippocampus in the dcKO mutants (Figure 5H).

Altogether, our results demonstrate that the chromatin-remodeling BAF complex is a crucial factor for ensuring the suppression of NE fate in the late neurogenic phase of corticogenesis.

### BAF Complexes Control NSC Proliferation and Differentiation in Early and Late Embryonic Stages via Distinct Epigenetic Mechanisms

Discrete histone marks activate or inhibit gene expression programs that regulate neural development. Modifications such as H3K4me2/3 and H3K27Me2/3, regulated by their corresponding histone lysine methyltransferases (KMTs) and demethylases (KDMs), are associated with transcriptional activation and repression respectively.

H3K4 is commonly targeted by numerous KMTs and KDMs. In pluripotent ESCs, H3K4me2 marks signaling pathway genes that are required for the transition of neural progenitors to mature neurons (Zhang et al., 2012). H3K4me2 marks are established mainly by KMT2C/D methyltransferases and are removed by the KDM1 (LSD1) demethylase (Shi et al., 2004), which we found to be highly expressed in late cortical progenitors (Figure S1). Interestingly, LSD1 is also highly expressed in late progenitors in the developing mouse retina, and its inhibition blocks the differentiation of rod photoreceptors during late developmental stages (Popova et al., 2016).

Our earlier work indicated that BAF complexes interact with the H3K27 demethylases KDM6A/B to promote cell proliferation and neuronal differentiation in early cortical development (Narayanan et al., 2015). Accordingly, loss of BAF complexes in early corticogenesis results in a global increase in repressive H3K27me3 marks and downregulation of genes important for progenitor proliferation and differentiation. These two outcomes following ablation of BAF complexes during late development suggest a dual function of BAF complexes in activating neuronal differentiation genes and suppressing proliferation-related pathways that may reflect independent processes mediated by distinct BAF complex cofactors. As in early stages, BAF complexes possibly induce neuronal differentiation by interacting with KDM6A/B to remove inactivating H3K27me3 marks on loci of neuronal differentiation genes (Figure 7H). In parallel, however, BAF complexes inhibit cell amplification, probably by interacting with KDM1A, and potentiate its demethylase activity in H3K4me2 removal at genomic loci of genes involved in Wnt signaling, mitotic cell cycling, and proliferation (Figure 7H).

Based on these data, we propose that, during late pallium development, endogenous BAF complexes associate with the coactivators KDM6A/B to promote neuronal differentiation, while inhibiting cell proliferation via KDM1A recruitment.

### BAF Complexes Suppress Wnt Signaling Activity

During cortical neurogenesis, temporal differentiation of NSCs leads to generation of cohorts of neurons with distinct layer identities, a process that depends on multiple regulatory pathways. Wnt signaling regulates the switch between proliferation and differentiation of cortical progenitors. Accordingly, ablation of *β-Catenin* or *Lrp6* (Wnt co-receptor) causes early cell cycle exit and premature differentiation of RGs into IPs and neurons (Draganova et al., 2015, Machon et al., 2007, Woodhead et al., 2006, Zhou et al., 2006). Conversely, persistent expression of β-catenin suppresses progenitor exit from mitosis, causing hyper-proliferation of NSCs through excessive symmetric division that consequently delays generation of TBR2^+^ IPs and neuronal differentiation in the pallium (Chenn and Walsh, 2002, Machon et al., 2007, Mutch et al., 2010, Wrobel et al., 2007). Interestingly, these phenotypes are reminiscent of the observed abnormalities in the *dcKO* cortex.

Previous studies demonstrated that, in mammalian non-neural cells, the core BAF subunit BRG1 positively regulates Wnt signaling at distinct levels (e.g., exerting a control of genes encoding for Wnt receptors and also modulating β-catenin-dependent transcriptional activity) (Barker et al., 2001, Griffin et al., 2011). Surprisingly, upon loss of BAF155 and BAF170 in late cortical progenitors, multiple components and targets of the canonical Wnt/β-catenin signaling were upregulated, suggesting that the SWI/SNF complex can act to control the Wnt/β-catenin signaling pathway in a tissue- and context-dependent manner. Pharmacological inhibition of Wnt/β-catenin signaling rescued the observed defects in cell proliferation, cell survival, and restored hippocampal morphology in the dcKO mutants, hence making us posit that BAF (SWI/SNF) complexes negatively regulate Wnt signaling during late cortical neurogenesis.

Altogether, our results indicate that the chromatin remodeler BAF plays a crucial role in late-stage development of mammalian cortex in two distinct ways. On one hand, BAF complexes induce heterochromatin formation at loci of cell cycle-, proliferation-, and Wnt-related genes, thereby suppressing their expression; and on the other, they facilitate the expression of neural differentiation-related genes by establishing euchromatin at related genomic regions. Together, these activities ensure the generation of appropriate numbers of NSCs and neurons in late cortical development.

## Experimental Procedures

### Transgenic Lines, Plasmids, and Antibodies

Animals were handled in accordance with the German Animal Protection Law. A list of transgenic lines, plasmids, and antibodies with detailed descriptions is provided in Supplemental Information.

### Mass Spectrometry, CoIP, ChIP-Seq, and RNA-Seq

Detailed descriptions were provided previously (Narayanan et al., 2015) and can be found in Supplemental Information.

### ISH, IF, 3D Reconstruction Spindle Angle Analysis, and Cell-Cycle Index

ISH, IF, 3D reconstruction, and determination of cell-cycle index were performed as previously described (Bachmann et al., 2016, Tuoc et al., 2013). The spindle angle analysis is described in Supplemental Information.

### *In Vivo* Pharmacological Treatment and *In Vivo* β-Catenin Transcriptional Activity Assay

A detailed description of treatment and assay are provided in Supplemental Information.

### Imaging, Quantification, Statistical Analysis, and Data Availability

Images were captured by confocal fluorescence microscopy (TCS SP5, Leica) and analyzed using an Axio Imager M2 (Zeiss) with a Neurolucida system. Images were further processed with Adobe Photoshop. IF signal intensities were quantified using ImageJ software, as described previously (Narayanan et al., 2015, Tuoc and Stoykova, 2008). The statistical quantification was carried out as average from at least three biological replicates. Details of statistical analyses and descriptions for histological experiments are presented in Table S6 and in Supplemental Information.

## Author Contributions

H.N. performed most characterization of dcKO phenotypes; C.K. and A.F. generated RNA-seq and ChIP-seq data; M.P. performed the protein-protein interaction study; L.P., G.S., and J.R. contributed to histological analyses; K.A.K. characterized hGFAP-Cre_ROSA-dtTOM mouse line; C.K. performed ChIP-qPCR; M.S.S. performed qPCR; J.F.S., R.H.S., U.T., and A.S. provided research tools and transgenic lines and contributed to discussions; T.T. conceived, supervised, and wrote the manuscript; C.K. designed all experiments related to RNA-seq and ChIP-seq and their confirmation, analyzed all related data, and wrote the corresponding parts of the manuscript; J.F.S., A.S., and A.F. offered suggestions for the study. The authors declare no competing financial interests.

## Accession Numbers

All RNA-seq and ChIP-seq data have been deposited in GEO under accession number GEO: GSE106711.

## Supplemental Information

Document S1. Supplemental Experimental Procedures and Figures S1–S7

Table S1. H3K27me3 ChIP-Seq and H3K4me2 ChIP-Seq Data of dcKO_*hGFAP*-Cre Pallium versus Control at E17.5, Related to Figure 1

Table S2. RNA-Seq and Gene Ontology Analysis (for Downregulated Genes, Upregulated Genes, WNT Signaling) in the dcKO_*hGFAP*-Cre Pallium at E17.5, Related to Figure 1

Table S3. Lists of Each of Those Gene Groups (Downregulated Neurogenesis Genes, Neurogenesis Genes with High H3K27me3, Upregulated Proliferation Genes, Cell-Cycle Genes with High H3K4me2, Upregulated Wnt, Wnt Target Genes) Regulated by BAF155/BAF170 in the dcKO_*hGFAP*-Cre Pallium at E17.5, Related to Figure S2

Table S4. List of Genes (Important for Development of Nervous System, Hippocampus, Cortical Layers, Proliferation, Differentiation, Orientation of the Mitotic Spindle, and Markers of Tight Junction, Astroglial, Adherens Junctions) with Altered Expression in dcKO_hGFAP-Cre Pallium, Related to Figures 2 and 3

Table S5. Genes Regulated by BAF155/BAF170 and Wnt Signaling in the dcKO_*hGFAP*-Cre Pallium at E17.5 with and without WNT Inhibitor Treatment, Related to Figure 7

Table S6. Statistical Analyses, Related to Figures 2–7 and S3–S7

Video S1. 3D Reconstruction of the E15.5 Hippocampus (See Also Figure S5A for IF Analysis to Define Hippocampal VZ, Plate, and Volume), Related to Figures 4 and 5

Document S2. Article plus Supplemental Information
