# Supplementary material for: Epigenetic Regulation by BAF Complexes Limits Neural Stem Cell Proliferation by Suppressing Wnt Signaling in Late Embryonic Development
Source: Stem Cell Reports. 2018 May 17;10(6):1734–50. doi: 10.1016/j.stemcr.2018.04.014 (PMC5993560; doi:10.1016/j.stemcr.2018.04.014)
Supplement: Document S1. Supplemental Experimental Procedures and Figures S1–S7 [file mmc1.pdf]

Stem Cell Reports, Volume *10*

Supplemental Information

Epigenetic Regulation by BAF Complexes Limits Neural Stem Cell Proliferation by Suppressing Wnt Signaling in Late Embryonic Development

Huong Nguyen, Cemil Kerimoglu, Mehdi Pirouz, Linh Pham, Kamila A. Kiszka, Godwin Sokpor, M. Sadman Sakib, Joachim Rosenbusch, Ulrike Teichmann, Rho H. Seong, Anastassia Stoykova, Andre Fischer, Jochen F. Staiger, and Tran Tuoc

SUPPLEMENTAL DATA

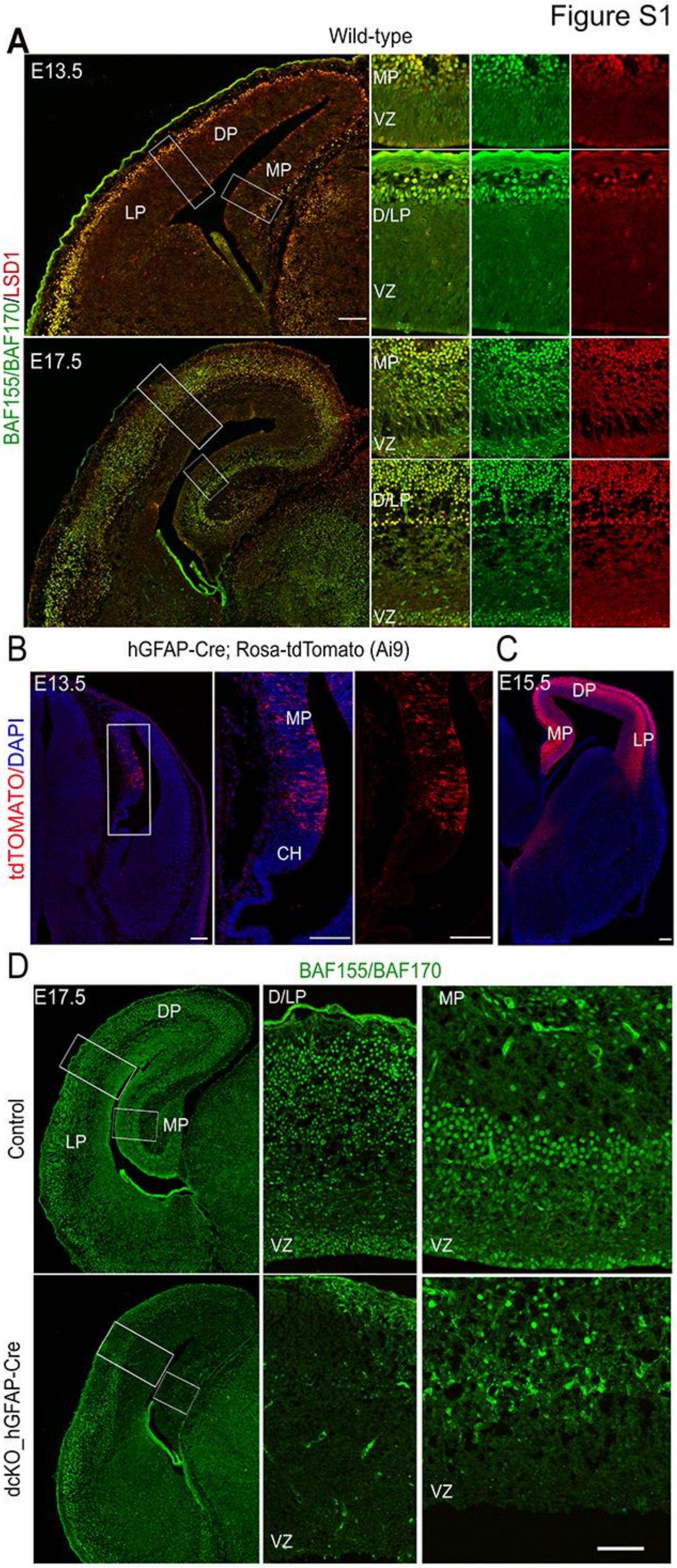

Figure S1 (related to figure 1). Co-expression of BAF155/BAF170 with H3K4 and H3K27 demethylases in the developing pallium; hGFAP-Cre activity in the developing pallium; and expression of BAF155/BAF170 in the dcKO_*hGFAP*-Cre pallium.

(A) Double-label IF analysis with anti-BAF155/BAF170 (green) and anti-LSD1/KDM1A (red) antibodies, showing co-expression of BAF155/BAF170 with LSD1/KDM1A in pallium cells. Co-expression of BAF155/BAF170 with UTX/KDM6A or JMJD3/ KDM6B in the developing forebrain was analyzed previously (Narayanan et al., 2015). (B, C) Immunostaining for dtTomato on cortical sections from hGFAP-Cre; Rosa-tdTomato (Ai9) embryos at E13.5 (B) and E15.5 (C). (B) The right panels are higher-magnification images from the fields in the MP indicated by white frames. By E13.5, recombination is restricted in MP but absent from D/LP and cortical hem (CH) (B). From E15.5 onward, Cre recombination was detected in entire pallium (C). (D) Compared to controls, expression of BAF155 and BAF170 was largely lost in the dcKO pallium, as shown by IF analysis with anti-BAF155/BAF170 antibodies. Abbreviations: VZ, ventricular zone; MP, medial pallium; DP, dorsal pallium; LP, lateral pallium; CH, cortical hem. Scale bars = 100 μm.

Figure S2

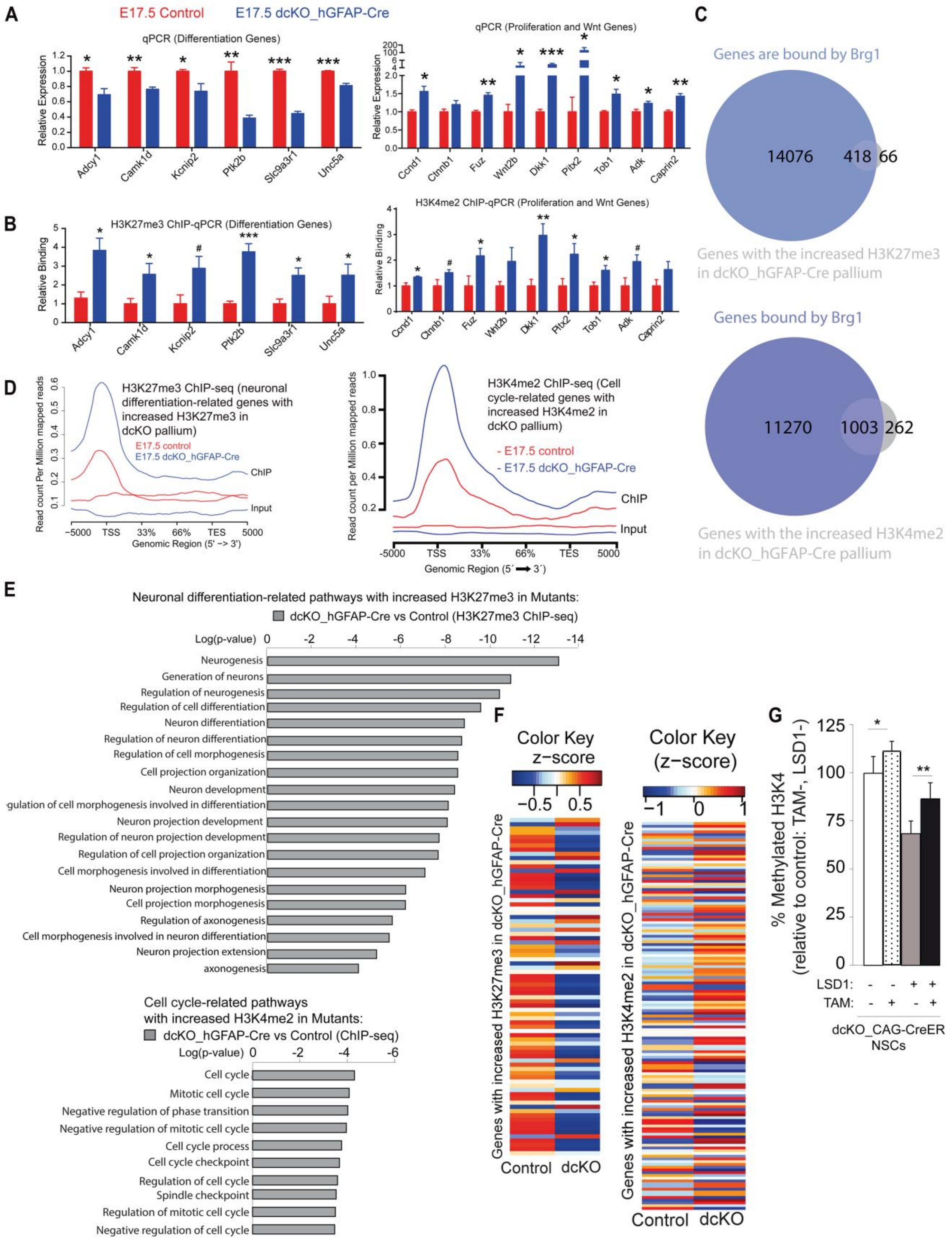
A
E17.5 Control
E17.5 dcKO_hGFAP-Cre
qPCR (Differentiation Genes)
Relative Expression
Adcy1
Camk1d
Kcnip2
Ptk2b
Slc9a3r1
Unc5a
qPCR (Proliferation and Wnt Genes)
Ccnd1
Ctnnb1
Fuz
Wnt2b
Dkk1
Pitx2
Tob1
Adk
Caprin2
B
H3K27me3 ChIP-qPCR (Differentiation Genes)
Relative Binding
H3K4me2 ChIP-qPCR (Proliferation and Wnt Genes)
C
Genes are bound by Brg1
14076
418
66
Genes with the increased H3K27me3 in dcKO_hGFAP-Cre pallium
Genes bound by Brg1
11270
1003
262
Genes with the increased H3K4me2 in dcKO_hGFAP-Cre pallium
D
Read count Per Million mapped reads
H3K27me3 ChIP-seq (neuronal differentiation-related genes with increased H3K27me3 in dcKO pallium)
E17.5 control
E17.5 dcKO_hGFAP-Cre
ChIP
Input
Genomic Region (5' -> 3')
H3K4me2 ChIP-seq (Cell cycle-related genes with increased H3K4me2 in dcKO pallium)
- E17.5 control
- E17.5 dcKO_hGFAP-Cre
E
Neuronal differentiation-related pathways with increased H3K27me3 in Mutants:
dcKO_hGFAP-Cre vs Control (H3K27me3 ChIP-seq)
Log(p-value)
Neurogenesis
Generation of neurons
Regulation of neurogenesis
Regulation of cell differentiation
Neuron differentiation
Regulation of neuron differentiation
Regulation of cell morphogenesis
Cell projection organization
Neuron development
gulation of cell morphogenesis involved in differentiation
Neuron projection development
Regulation of neuron projection development
Regulation of cell projection organization
Cell morphogenesis involved in differentiation
Neuron projection morphogenesis
Cell projection morphogenesis
Regulation of axonogenesis
Cell morphogenesis involved in neuron differentiation
Neuron projection extension
axonogenesis
Cell cycle-related pathways with increased H3K4me2 in Mutants:
dcKO_hGFAP-Cre vs Control (ChIP-seq)
Cell cycle
Mitotic cell cycle
Negative regulation of phase transition
Negative regulation of mitotic cell cycle
Cell cycle process
Cell cycle checkpoint
Regulation of cell cycle
Spindle checkpoint
Regulation of mitotic cell cycle
Negative regulation of cell cycle
F
Color Key z-score
Color Key (z-score)
Genes with increased H3K27me3 in dcKO_hGFAP-Cre
Genes with increased H3K4me2 in dcKO_hGFAP-Cre
Control
dcKO
G
% Methylated H3K4 (relative to control: TAM-, LSD1-)
LSD1:
TAM:
dcKO_CAG-CreER NSCs

Figure S2 (related to figures 1, 2, 4). Characterization of H3K27me3 and H3K4me2 marks in dcKO_hGFAP-Cre cortex.

(A) qPCR confirmation of selected neuronal differentiation-related genes downregulated, proliferation- and Wnt-related genes upregulated in dcKO_*hGFAP*-Cre embryos at E17.5 (B) ChIP-qPCR confirmation of H3K27me3 levels at selected neuronal differentiation-related genes downregulated and H3K4me2 levels at selected proliferation and Wnt-related genes upregulated in dcKO_*hGFAP*-Cre embryos at E17.5. (C) Overlap between genes bound by BRG1 (GSE37151; Attanasio et al., 2014) and those with increased H3K27me3 and those with increased H3K4me2 in dcKO embryos (hypergeometric test: *p*-value < 0.0001). (D) General profile plots of H3K27me3 at neuronal differentiation-related genes with increased H3K27me3 and H3K4me2 at cell cycle-related genes with increased H3K4me2 in dcKO_*hGFAP*-Cre embryos (*p*-value < 0.0001, Student's t-test). (E) Neuronal differentiation-related genes have increased H3K27me3 and Cell cycle-related genes have increased H3K4me2 (*p*-value < 0.01) in dcKO_*hGFAP*-Cre embryos at E17.5. (F) Heatmaps depicting the expression changes in neural differentiation-related genes that have increased H3K27me3 and in cell cycle-related genes that have increased H3K4me2 in dcKO_*hGFAP*-Cre embryos at E17.5. (G) In the LSD1 demethylase activity quantification assay, cultured dcKO_CAG-Cre NSCs were nucleofected with a mammalian expression vector for LSD1/KDM1A. The Cre-mediated deletion of BAF155 and BAF170 alleles was induced by adding TAM. Compared to control (LSD1-, TAM-, white bar), overexpression of LSD1 (LSD1+, TAM-, grey bar) decreased the methylated H3K4. In absence of BAF complexes (LSD1+, TAM, black bar) LSD1 displayed its low H3K4 demethylase activity. Values are presented as means ± SEMs (**p*-value < 0.05, ****p*-value < 0.01, ****p*-value < 0.005). Experimental replicates (n) = 4 (A, B), 6 (G).

Figure S3

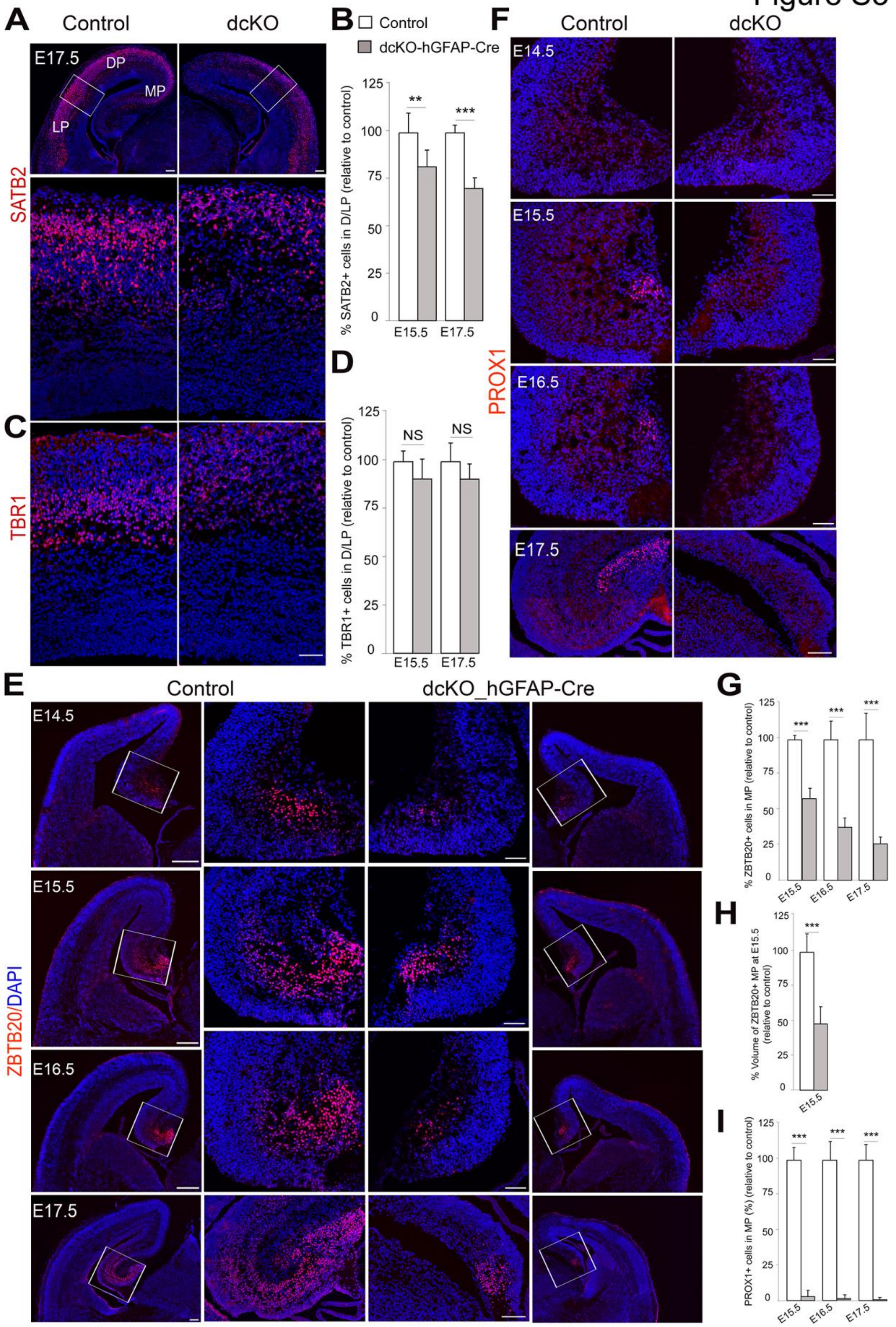

A
Control
dcKO
E17.5
DP
MP
LP
SATB2
B
Control
dcKO-hGFAP-Cre
% SATB2+ cells in D/LP (relative to control)
125
100
75
50
25
0
**
***
E15.5
E17.5
C
TBR1
D
% TBR1+ cells in D/LP (relative to control)
NS
NS
F
PROX1
E14.5
E15.5
E16.5
E17.5
E
Control
dcKO_hGFAP-Cre
ZBTB20/DAPI
G
% ZBTB20+ cells in MP (relative to control)
E16.5
H
% Volume of ZBTB20+ MP at E15.5 (relative to control)
I
PROX1+ cells in MP (%) (relative to control)

Figure S3 (related to figures 2, 3). BAF155 and BAF170 are essential for neurogenesis in the developing cortex and hippocampus.

(A–B) IF (A, C) and quantitative (B, D) analyses of the neurogenesis phenotype using the late-born neuronal marker SATB2 (A, B) and early-born neuronal marker TBR1 (C, D). (E - I) IF (E, F) and quantitative (G-I) analyses for the neuronal markers ZBTB20 in the hippocampus (E, G, H) and PROX1 in the dentate gyrus (F, I) revealed that hippocampal sections of the MP of mutants have a reduced number of $ZBTB20^{+}$ neurons and $PROX1^{+}$ neurons at E15.5–E17.5 compared to controls. (H) Quantification of the $ZBTB20^{+}$ hippocampal volume performed across the entire hippocampus using 3D reconstruction (see also Figure S5A and Movie S1). Values are expressed as means ± SEMs (**P* < 0.05; ***P* < 0.01; ****P* < 0.005). Experimental replicates (n) = 6 (B, D, G, I), 4 (H). Abbreviations: MP, medial pallium; DP, dorsal pallium; LP, lateral pallium. Scale bars = 100 μm (10x; A,E), 50 μm (25x; A,C), and 50 μm (40x; E,F).

Figure S4

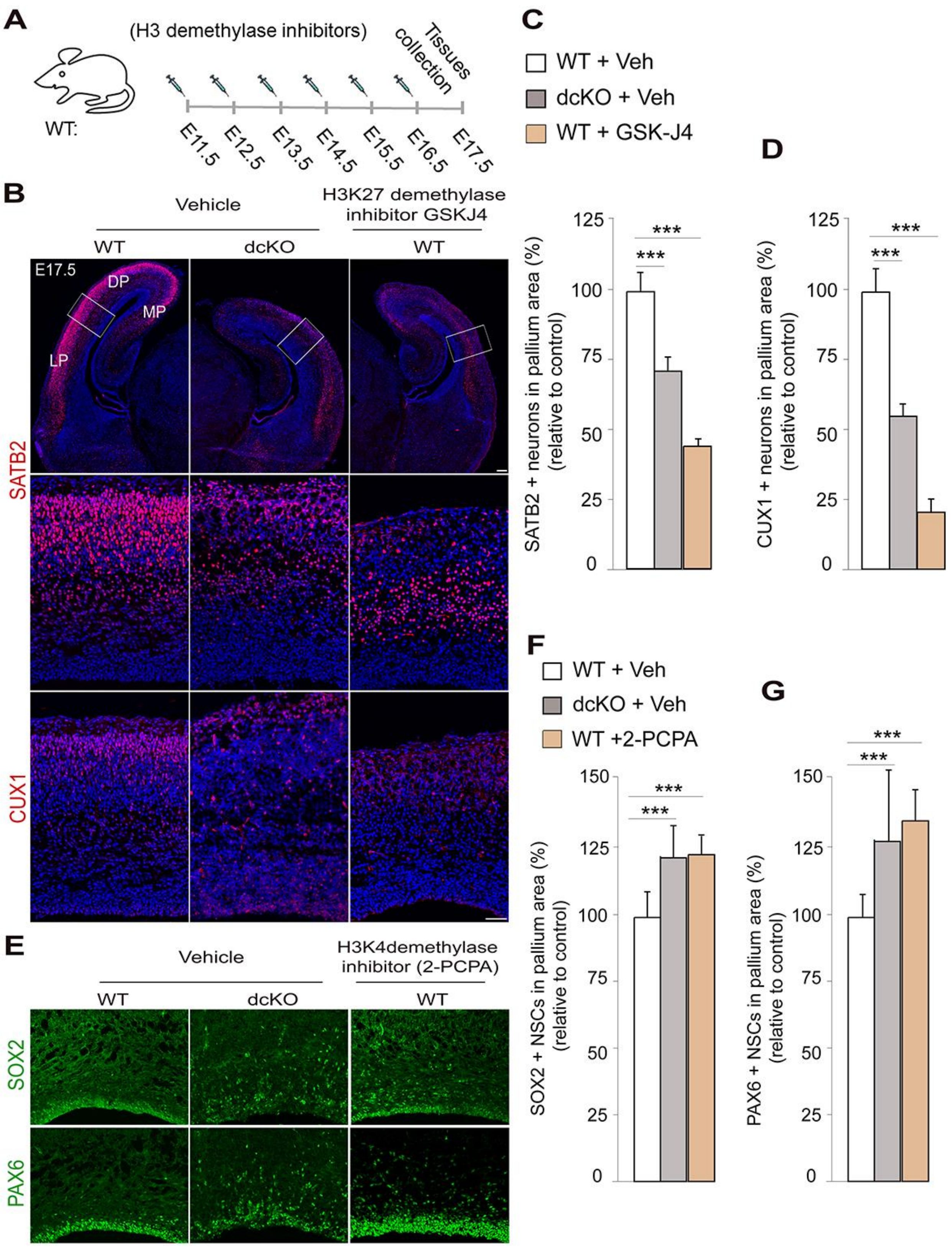
A
(H3 demethylase inhibitors)
Tissues collection
WT:
E11.5
E12.5
E13.5
E14.5
E15.5
E16.5
E17.5
B
Vehicle
H3K27 demethylase inhibitor GSKJ4
WT
dcKO
WT
E17.5
DP
MP
LP
SATB2
CUX1
C
WT + Veh
dcKO + Veh
WT + GSK-J4
SATB2 + neurons in pallium area (%) (relative to control)
D
CUX1 + neurons in pallium area (%) (relative to control)
E
Vehicle
H3K4demethylase inhibitor (2-PCPA)
WT
dcKO
WT
SOX2
PAX6
F
WT + Veh
dcKO + Veh
WT +2-PCPA
SOX2 + NSCs in pallium area (%) (relative to control)
G
PAX6 + NSCs in pallium area (%) (relative to control)

Figure S4 (related to figures 2, 4). Elevated level of H3K27me3 and H3K4me2 by inhibition of H3K27 and H3K4me2 demethylases caused the defect in neuronal differentiation in developing pallium.

(A) Experimental paradigms in which WT embryos were treated with H3K27 demethylase inhibitor GSK-J4 and H3K4 demethylase inhibitor 2-PCPA. (B–G) IF (B, E) and quantitative (C, D, F, G) analyses are to compare cortical phenotype of Veh-treated WT, Veh-dcKO and WT which is treated with GSK-J4 (a-d) or with 2-PCPA (e-g). (C, D) Statistical comparisons indicate that the increased level of H3K27me3 in cortices of dcKO embryos or GSK-J4 treated embryos decreased the number of late-born SATB2^{+} (C) and CUX1^{+} neurons (D) compared to Veh-treated WT. Likewise, the increased level of H3K4me2 in cortices of dcKO embryos or 2-PCPA treated embryos increased the number of SOX2^{+} (F) and PAX6^{+} NSCs (G) compared to Veh-treated WT. Values are expressed as means ± SEMs (*P < 0.05; **P < 0.01; ***P < 0.005). Experimental replicates (n) = 4 (C, D, F, G). Abbreviations: MP, medial pallium; DP, dorsal pallium; LP, lateral pallium. Scale bars = 100 μm (10x, B) and 50 μm (40x, B).

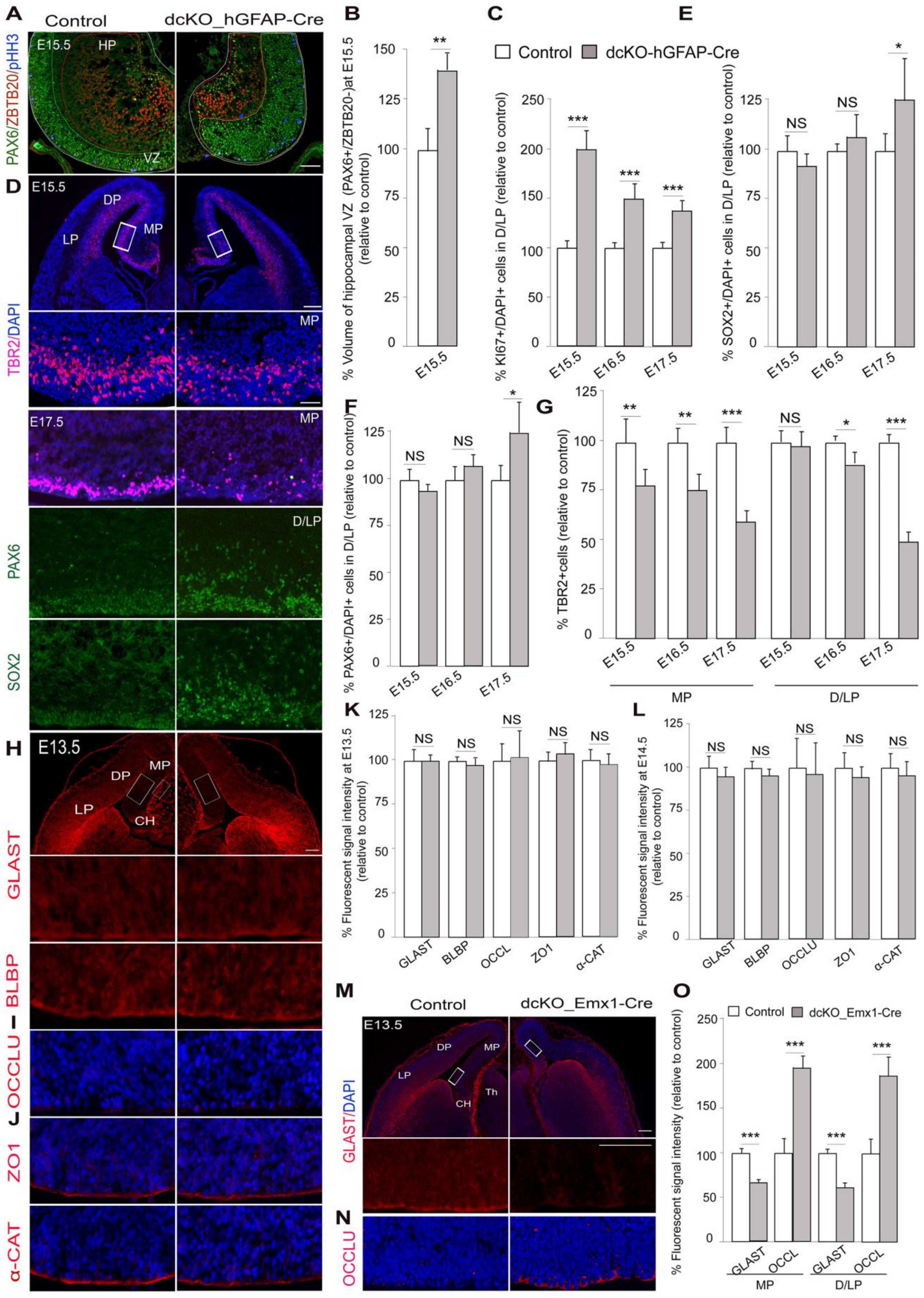
A
Control
dcKO_hGFAP-Cre
PAX6/ZBTB20/pHH3
E15.5
HP
VZ
B
% Volume of hippocampal VZ (PAX6+/ZBTB20-)at E15.5 (relative to control)
C
Control
dcKO-hGFAP-Cre
% KI67+/DAPI+ cells in D/LP (relative to control)
E
% SOX2+/DAPI+ cells in D/LP (relative to control)
NS
D
E15.5
DP
MP
LP
TBR2/DAPI
E17.5
PAX6
D/LP
SOX2
F
% PAX6+/DAPI+ cells in D/LP (relative to control)
G
% TBR2+cells (relative to control)
E15.5
E16.5
E17.5
MP
D/LP
H
E13.5
CH
GLAST
BLBP
I
OCCLU
J
ZO1
α-CAT
K
% Fluorescent signal intensity at E13.5 (relative to control)
GLAST
BLBP
OCCL
ZO1
α-CAT
L
% Fluorescent signal intensity at E14.5 (relative to control)
OCCLU
M
Control
dcKO_Emx1-Cre
Th
GLAST/DAPI
N
OCCLU
O
Control
dcKO_Emx1-Cre
% Fluorescent signal intensity (relative to control)

Figure S5 (related to figures 4, 5). Loss of BAF155 and BAF170 in dcKO mutants has a profound effect on the pool of NSCs, expression of adherens junction molecules.

(A) Representative images of triple IF analysis of PAX6 to visualize hippocampal VZ, ZBTB20 to mark hippocampal plate (HP) and PHH3 to label mitotic M-phase cells in our 3D reconstruction analysis. Total hippocampal volume was surrounded by white line. (B) Quantitative analyses of the volume of the hippocampal VZ ($PAX6^+/ZBTB20^-$) in the dcKO_*hGFAP*-Cre mutant and control at E15.5 (see also Movie S1 for 3D reconstruction analysis). (C–G) IF (D) and quantitative (C, E, F and G) analyses of the mitotic marker KI67 (C), RG markers PAX6 and SOX2 (D, E and F), and IP marker TBR2 in the MP and D/LP of dcKO_*hGFAP*-Cre mutants and control (G). Generally, the loss of BAF155 and BAF170 in dcKO_*hGFAP*-Cre mutants had a more profound effect on the pool of NSCs in the MP (see also Fig. 4E–L) than in the D/LP. It should be noted that the loss of BAF155 and BAF170 in the E15.5–E16.5 D/LP did not affect the pool of $PAX6^+/SOX2^+$ RGs; however, the number of $KI67^+$ active mitotic progenitors was already increased. Counting was done in selected frames, denoted by white boxes. (H-L) IF (H-J) and quantitative (K, L) analyses revealed that in contrast to later stages (E15.5. E16.5), there is no obvious difference in expression level of GLAST, BLBP, ZO1, α-CATENIN and OCCLUDIN between the control and dcKO_hGFAP-Cre pallium at E13.5 and E14.5. (M–O) Immunostaining of coronal sections from the control and dcKO_*Emx1*-Cre pallium at E13.5 for GLAST (M) and OCCLUDIN (N) revealed an altered cell identity from $GLAST^{high+}/OCCLUDIN^-$ RGs in controls to $GLAST^{low+}/OCCLUDIN^{high+}$ NEs in the dcKO_*Emx1*-Cre mutant. (O) Quantitative analyses of panels M–N are shown. Values are expressed as means ± SEMs (*$P < 0.05$; **$P < 0.01$; ***$P < 0.005$). Experimental replicates (n) = 4 (B), 6 (C, E, F, G, K, L, O). Abbreviations: MP, medial pallium; DP, dorsal pallium; LP, lateral pallium; HP, hippocampal plate; VZ, ventricular zone; CH, cortical hem; Th, thalamus. Scale bar = 100 μm.

Figure.S6

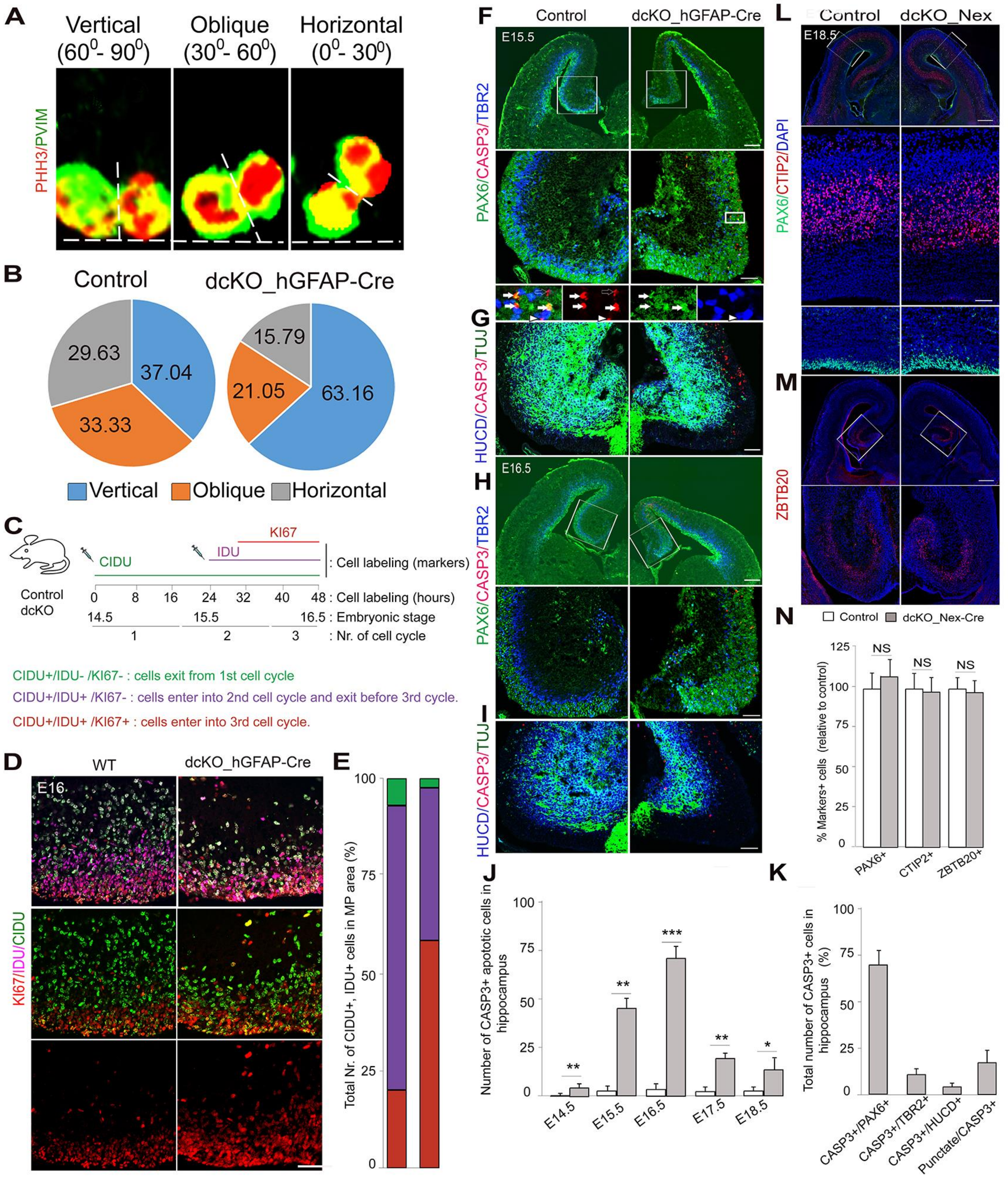
A
Vertical (60⁰- 90⁰)
Oblique (30⁰- 60⁰)
Horizontal (0⁰- 30⁰)
PHH3/PVIM
B
Control
dcKO_hGFAP-Cre
29.63
37.04
33.33
15.79
21.05
63.16
Vertical
Oblique
Horizontal
C
KI67
IDU
CIDU
: Cell labeling (markers)
Control
dcKO
0 8 16 24 32 40 48 : Cell labeling (hours)
14.5 15.5 16.5 : Embryonic stage
1 2 3 : Nr. of cell cycle
CIDU+/IDU- /KI67- : cells exit from 1st cell cycle
CIDU+/IDU+ /KI67- : cells enter into 2nd cell cycle and exit before 3rd cycle.
CIDU+/IDU+ /KI67+ : cells enter into 3rd cell cycle.
D
WT
dcKO_hGFAP-Cre
E16
KI67/IDU/CIDU
E
Total Nr. of CIDU+, IDU+ cells in MP area (%)
100
75
50
25
0
F
Control
dcKO_hGFAP-Cre
E15.5
PAX6/CASP3/TBR2
G
HUCD/CASP3/TUJ
H
E16.5
PAX6/CASP3/TBR2
I
HUCD/CASP3/TUJ
J
Number of CASP3+ apotic cells in hippocampus
100
75
50
25
0
**
**
***
**
*
E14.5
E15.5
E16.5
E17.5
E18.5
K
Total number of CASP3+ cells in hippocampus (%)
100
75
50
25
0
CASP3+/PAX6+
CASP3+/TBR2+
CASP3+/HUCD+
Punctate/CASP3+
L
Control
dcKO_Nex
E18.5
PAX6/CTIP2/DAPI
M
ZBTB20
N
Control
dcKO_Nex-Cre
% Markers+ cells (relative to control)
125
100
75
50
25
0
NS
NS
NS
PAX6+
CTIP2+
ZBTB20+

Figure S6 (related to figures 2-5). The spindle orientation, selective apoptosis of RGs in dcKO_hGFAP-Cre mutants and phenotypes of cortical neuron-specific dcKO_Nex-Cre mutants.

(A, B) IF analyses in control and dcKO mutants using antibodies against PHH3 and PVIM , to visualize the orientation of spindles (vertical, oblique, horizontal) (A), and quantitative analyses (B) indicate that loss of BAF155 and BAF170 induces proliferative, symmetric division. (C) Experimental paradigm for determining cell-cycle parameters and color scheme for immunolabeling of micrographs (in D). (D) Images show triple-label IHC of cortical sections with antibodies for CIDU (48-h labeling), IDU (24-h labeling) to mark both exited and cycling progenitors, KI67 to label proliferating progenitors at E16.5. (E) Statistical comparisons indicated a decreased number of cells, which exit from 1st and 2nd cell cycles and an increased number of progenitors, which enter the 3rd cycle in the medial pallium of dcKO embryos compared with control. (F-K) Triple IF for PAX6/TBR2/CASP3 and HuCD/TUBB3/CASP3 (F–I) and quantitative analyses (J, K) show that the developing hippocampus of the dcKO-*hGFAP*-Cre embryo has a high number of CASP3^{+} apoptotic cells at E14.5–E18.5 (F–J). Notably, most CASP3^{+} cells are immunoreactive for PAX6 (white filled arrows) or exhibit punctate forms (late phase of apoptosis, empty arrows), whereas less extended cells are TBR2^{+} IPs and HUCD^{+}/TUBB3^{+} neurons (F, H, K; arrowhead). Lower panels are higher-magnification images from the fields indicated by white rectangles. Noted that a similar image of single channel for Pax6 is shown in Figure 4F. (L–N) Phenotype analysis of the pallium from neuron-specific dcKO_*Nex*-Cre mutants. Immunostaining of coronal sections of E18.5 brains with antibodies specific for RGs (PAX6; L), layer 5 neurons (CTIP2; L) and hippocampal neurons (ZBTB20; M), and quantification of results in (L) showed no discernible differences in the number of immunopositive cells between the dcKO_*Nex*-Cre and control pallium. Values are presented as means ± SEMs (**P* < 0.05, ****P* < 0.01, ****P* < 0.005). Experimental replicates (n) = 6 (E, J, K), 4 (N). Scale bars = 100 μm (10x; F, H, L, M), 50 μm (40x; F, G, H and I), 100 μm (L) and 50 μm (D).

Figure S7

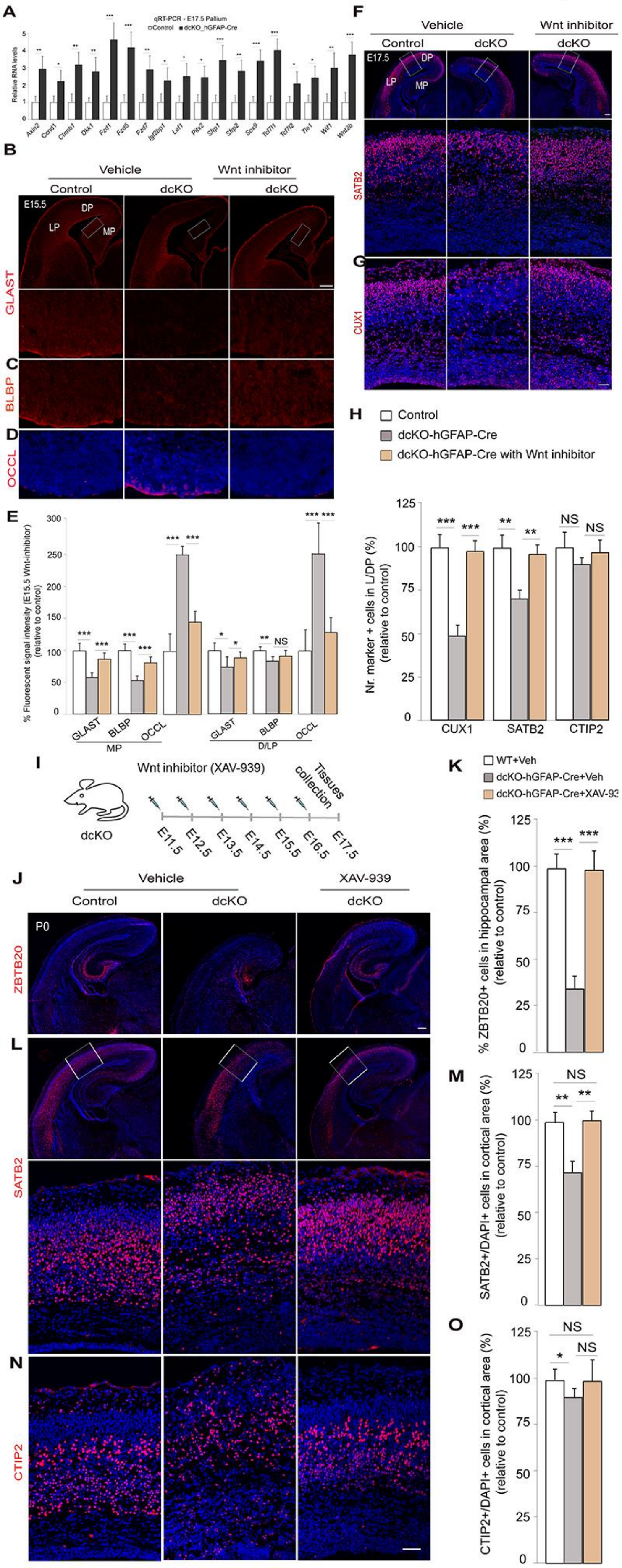

qRT-PCR - E17.5 Pallium
Control
dcKO_hGFAP-Cre
Relative RNA levels
Axin2
Ccnd1
Ctnnb1
Dkk1
Fzd1
Fzd5
Fzd7
Igf2bp1
Lef1
Pitx2
Sfrp1
Sfrp2
Sox9
Tcf7l1
Tcf7l2
Tle1
Wif1
Wnt2b
Vehicle
Wnt inhibitor
Control
dcKO
E15.5
DP
LP
MP
GLAST
BLBP
OCCL
% Fluorescent signal intensity (E15.5 Wnt-inhibitor) (relative to control)
D/LP
E17.5
SATB2
CUX1
Control
dcKO-hGFAP-Cre
dcKO-hGFAP-Cre with Wnt inhibitor
Nr. marker + cells in L/DP (%) (relative to control)
CTIP2
NS
Wnt inhibitor (XAV-939)
Tissues collection
dcKO
E11.5
E12.5
E13.5
E14.5
E15.5
E16.5
E17.5
WT+Veh
dcKO-hGFAP-Cre+Veh
dcKO-hGFAP-Cre+XAV-93
% ZBTB20+ cells in hippocampal area (%) (relative to control)
XAV-939
P0
ZBTB20
SATB2+/DAPI+ cells in cortical area (%) (relative to control)
CTIP2+/DAPI+ cells in cortical area (%) (relative to control)

Figure S7 (related to figures 6, 7). Suppression of Wnt signaling in the developing pallium by BAF complexes in the developing forebrain.

(A) Expression of upregulated Wnt genes identified by RNA-Seq analysis (see Fig. 6A, B) was further verified by qRT-PCR. (B–E) IF (B–D) and quantitative (E) analyses of dcKO_*hGFAP*-Cre mutants at E15.5 showing the effects of treatment with the Wnt inhibitor ICG-001 on expression of the RG markers GLAST (B) and BLBP (C), and the NE marker OCCLUDIN (D). IF (F-G) and quantitative (H) analyses of dcKO_*hGFAP*-Cre mutants at E17.5, showing the effects of treatment with WNTi on pools of SATB2$^+$ (F), CUX1$^+$ (G) cortical neurons in L/DP. Quantitative analyses indicated that inhibition of Wnt signaling largely rescues defects in L/DP in dcKO_*hGFAP*-Cre mutants (H). (I–O) Rescued cortical defects in dcKO_hGFAP-Cre mutant by Wnt inhibitor XAV-939. (I) Experimental paradigm in which WT (control), dcKO embryos were treated with Wnt inhibitor XAV-939 or Vehicle (Veh). (J–O) IF (J, L, N) and quantitative (K, M, O) analyses are to compare population of cortical neuron: ZBTB20$^+$ hippocampal neurons (J, K), SATB2$^+$ late-born neurons (L, M), CTIP2$^+$ early-born neurons (N, O) in Veh-treated WT, Veh-treated dcKO and WNTi-treated WT. Quantitative analyses indicated that inhibition of Wnt signaling by XAV-939 largely rescues the neurogenesis defects in dcKO_*hGFAP*-Cre mutants. Values are expressed as means ± SEMs (*$P < 0.05$; **$P < 0.01$; ***$P < 0.005$; n = 4). Experimental replicates (n) = 4 (A, H, K, M, O), 6 (E). Abbreviations: MP, medial pallium; DP, dorsal pallium; LP, lateral pallium. Scale bars = 100 μm (10x; B, F), 50μm (40x; G), 100 μm (10x; J), and 50 μm (40x; N).

SUPPLEMENTAL EXPERIMENTAL PROCEDURES

Transgenic mice

BAF155$^{f/f}$ (Choi et al., 2012), *BAF170*$^{f/f}$ (Tuoc et al., 2013), *Emx1*-Cre (Gorski et al., 2002), *hGFAP*-Cre (Zhuo et al., 2001), *Nex*-Cre (Goebbels et al., 2006) and *Rosa-tdTomato (Ai9)(Madisen et al., 2010)* mice were maintained in a C57BL6/J background. Animals were handled in accordance with the German Animal Protection Law.

Plasmids

Plasmids used in this study: pCIG2-ires-eGFP, pCIG2-Cre-ires-eGFP (gift from Dr Francois Guillemot, NIMR London); 8XTOPFLASH (TOP) (Veeman et al., 2003) and Super8XFOPFLASH (FOP) (Veeman et al., 2003) were gifts from Randall Moon (Addgene plasmid # 12456, 12457)

Antibodies

The following polyclonal (pAb) and monoclonal (mAb) primary antibodies used in this study were obtained from the indicated commercial sources: AP2γ mouse mAb (1:100; Cat. ab87475, Abcam), BAF170 rabbit pAb (Cat. IHC-00213; Bethyl), BAF170 rabbit pAb (Cat. HPA021213; Sigma), BAF155 rabbit pAb (1:20; Cat. sc-10756; Santa Cruz), BAF155 mouse mAb (1:100; Cat. sc-48350X; Santa Cruz), Brn2 goat pAb (1:100; Cat. sc-6029, Santa Cruz), BLBP rabbit pAb (1:200; Cat. AB9558/ABN14; Chemicon), CASP3 rabbit pAb (1:100; Cat. #9661S; Cell Signaling), CTIP2 rat pAb (1:200; Cat. ab18465; Abcam), GLAST pig pAb (1:500; Cat. Af1000-1; Frontier), CIDU rat pAb (1:100; Cat. OBT-0030; Accurate), H3K27me3 rabbit pAb (Cat. 07-449; Upstate),

KDM6A/UTX rabbit pAb (Cat. sc-292326; Santa Cruz), KDM6B/jmjd3 rabbit pAb (De Santa et al., 2007), KI67 rabbit pAb (1:50; Cat. VP-RM04; Vector), HuCD mouse mAb (1:20; Cat. A21271; Invitrogen), LSD1 rabbit pAb (1:100; Cat. #2139s; Cell Signaling), SATB2 mouse mAb (1:200; Cat. ab51502; Abcam), SOX2 mouse mAb (1:100; Cat. MAB2018; R&D Systems), PROX1 rabbit pAb (1:1000; Cat. PRB-238C; Covance), PAX6 mouse mAb (1:100; Developmental Studies Hybridoma Bank), PAX6 rabbit pAb (1:200; Cat. PRB-278P; Covance), Flag mouse mAb (1:1000; Cat. F1804 Sigma), phospho-H3 rabbit mAb (1:200; Cat. Millipore), phospho-H3 rat pAb (1:300; Cat. Abcam), PVIM mouse mAb (1:500; Cat. D076-3 MBL), OCCLUDIN rabbit pAb (1:50; Cat. 40-4700 Thermo Fisher), TUBB3 mouse mAb (Tuj1, 1:500; Cat. Chemicon), TBR2 rabbit pAb (1:200; Cat. ab23345; Abcam), TBR1 rabbit pAb (1:300; Cat. AB9616; Chemicon), ZBTB20 rabbit pAb (1:50; Cat. HPA016815; Sigma), and RFP rabbit pAb (1:10000; Cat. 600-401-379; Biomol/Rockland).

Secondary antibodies used were horseradish peroxidase (HRP)-conjugated goat anti-rabbit IgG (1:10000; Cat. 111-035-003; Covance), HRP-conjugated goat anti-mouse IgG (1:5000; Cat. 115-035-003; Covance), HRP-conjugated goat anti-rat IgG (1:10000; Cat. 112-035-143; Covance), and Alexa 488-, Alexa 568-, Alexa 594- and Alexa 647-conjugated IgG (various species, 1:400; Molecular Probes).

Generation of dcKO mutants

To eliminate *BAF155* and *BAF170* in early cortical progenitors, late cortical progenitors, or projection neurons, we used the early progenitor-active *Emx1*-Cre (Gorski et al., 2002), late progenitor-active *hGFAP*-Cre (Zhuo et al., 2001) and neuron-specific *Nex*-Cre (Goebbels et al., 2006) mouse lines, respectively. Heterozygous

animals (*BAF155*$^{fl/+}$, *BAF170*$^{fl/+}$, *Cre*) were used as controls. Mutants crossed with *Emx1*-Cre or *hGFAP*-Cre died soon after birth.

Chromatin immunoprecipitation (ChIP)

ChIP assays performed on the pallium from control (n = 4) and dcKO_*hGFAP*-Cre E17.5 (n = 4) littermate embryos were performed as described previously (Narayanan et al., 2015). Briefly, tissues were homogenized in sucrose solution (0.32 M sucrose, 5 mM $Cacl_2$, 5 mM $Mg(Ac)_2$, 0.1 mM EDTA, 50 mM HEPES pH 8, 1 mM DTT, 0.1% Triton X-100), and then fixed in 37% formaldehyde. After stopping fixation by adding 1.25 M glycine, samples were washed with Nelson buffer (140 mM NaCl, 20 mM EDTA pH 8, 50 mM Tris pH 8, 0.5% NP-40, 1% Triton X-100) and sonicated in RIPA buffer (140 mM NaCl, 1 mM EDTA, 1% Triton X-100, 0.1% sodium deoxycholate, 10 mM Tris pH 8, 1% SDS).

For ChIP against histone marks 500 ng of chromatin with either 4 µg of anti-H3K4me2 antibody (Millipore) or 2 µg of anti-H3K27me3 antibody (Millipore) were used for each experiment, with input DNA of 50 ng. For ChIP against histone demethylases 10 µg of chromatin with either 10 µg of anti-LSD1 antibody (Abcam) or 10 µg of anti-JMJD3 antibody (Abcam) were used for each experiment with input DNA of 100 ng. After incubating overnight, samples were incubated with 15 µl of Protein A-coated beads (Diagenode) that had been blocked by incubating with 0.5% bovine serum albumen (BSA). Beads were washed with IP buffer (140 mM NaCl, 1% NP-40, 0.5% sodium deoxycholate, 50 mM Tris pH 8, 20 mM EDTA, 0.1% SDS) and wash buffer (100 mM Tris pH 8, 500 mM LiCl, 1% NP-40, 1% sodium deoxycholate, 20 mM EDTA), and chromatin was eluted with 0.1 µg/µl of RNase A diluted in 10 mM Tris (pH 8). De-

crosslinking was carried out in Wiemann buffer (100 mM Tris pH 8, 20 mM EDTA, 2% SDS) with Proteinase K (1 µg/µl). DNA was eluted in 10 mM Tris (pH 8).

ChIP-Sequencing

Libraries were prepared with a NEBNext Ultra DNA Library Prep Kit for Illumina (NEB) and analyzed using QuBit and an Agilent 2100 Bioanalyzer. Input DNA (50 ng) was isolated from each sample and then pooled separately for each group.

Base calling and FASTQ conversions were performed using standard Illumina scripts, as described previously (Halder et al., 2016; Narayanan et al., 2015). Quality control was also performed for each sample using FastQC (www.bioinformatics.babraham.ac.uk/projects/fastqc). Reads were mapped to the mm10 mouse reference genome using STAR aligner v2.3.0 (Djebali et al., 2012). BAM files were filtered leaving only high-quality reads [MAPQ !=(0,2,3,4)] as described previously (Halder et al., 2016).

BAM files of replicates from the same group were combined using the *merge* function of SAMTools (Li et al., 2009). Genomic profile plots were created from combined BAM files using NGSPlot (Shen et al., 2014). Wiggle (WIG) files were created from these BAM files using the script available in the MEDIPS package of Bioconductor (Lienhard et al., 2014). Visualization of individual gene loci was performed with Integrated Genome Browser (Nicol et al., 2009) using these WIG files.

Peaks were called using MACS2, with $q < 0.1$ (Feng et al., 2012). Differential binding analyses were performed using the DiffBind package of Bioconductor (Ross-Innes et al., 2012) with the DESEQ2 option for differential analysis. Peak annotation was performed using HOMER (Heinz et al., 2010) and homemade scripts.

RNA-Sequencing

RNA was extracted (RNeasy kit; Qiagen) from the pallium of control (n = 4) and dcKO_*hGFAP*-Cre (n = 3) E17.5 littermate embryos. cDNA libraries were prepared using the TruSeq RNA Sample Preparation v2 Kit. DNA was quantified using a Nanodrop spectrophotometer, and its quality was assessed using an Agilent 2100 Bioanalyzer.

Base calling, fastq conversion, quality control, and read alignments were all performed as outlined for ChIP-Seq. Reads were aligned to mouse genome mm10 and counted using FeaturesCount (http://bioinf.wehi.edu.au/featureCounts/). Differential expression was assessed using DESeq2 from Bioconductor (Love et al., 2014). Functional GO enrichment analyses were performed using ToppGene (Chen et al., 2009).

The high-throughput RNA-seq and ChIP-Seq data will be deposited in the NCBI Gene Expression Omnibus and made accessible through GEO Series accession numbers upon acceptance of the manuscript.

qPCR, ChIP-qPCR and Western blot analyses

qPCR and Western blot analyses were performed as described previously (Tuoc and Stoykova, 2008). Briefly, qPCR for confirmation of gene expression changes cDNA was synthesized using Transcriptor High Fidelity cDNA Synthesis Kit (Roche) and the results were normalized to housekeeping gene hypoxanthine phosphoribosyltransferase (*Hprt1*). For ChIP-qPCR the results were normalized against input DNA. All qPCR reactions were performed in Roche 480 Light Cycler using

SYBR Green.To validate the upregulated expression of Wnt-related genes in our RNA-Seq dataset, we performed qPCR using the mouse WNT Signaling Pathway RT2 Profiler PCR Array (PAMM-043Z; Qiagen) and mouse WNT Signaling Targets RT2 Profiler PCR Array (PAMM-243Z; Qiagen). The following primers were used for gene expression qPCR (5’ – 3’):

**Primer**	**Sequence (5' - 3')**
Adk_forward	CGCAGAATTCAATGAAAGTGG
Adk_reverse	GCATCCAAAGAATGTTGCTG
Adcy1_forward	AGATGGGACTTGACATGATCG
Adcy1_reverse	CGCATGTTCAGGTCTACTTCAG
Camk1d_forward	GAGTTTGATTCCCCCTACTGG
Camk1d_reverse	TCCATCAGATTCCGAATGAAG
Caprin2_forward	AGTTAAATGTAGAACCCAAAGATGTG
Caprin2_reverse	CCTCAATACTGGATCCTTTGGA
Ccnd1_forward	TTTCTTTCCAGAGTCATCAAGTGT
Ccnd1_reverse	TGACTCCAGAAGGGCTTCAA
Ctnnb1_forward	TGCAGATCTTGGACTGGACA
Ctnnb1_reverse	AAGAACGGTAGCTGGGATCA
Dkk1_forward	CCGGGAACTACTGCAAAAAT
Dkk1_reverse	CCAAGGTTTTCAATGATGCTT
Fuz_forward	CCTTAGAAACTTCTACACGCTGGT

Fuz_reverse	GGCTGGGTAGACTGTGTCCT
Kcnip2_forward	CTGCCCTCAGTCAGTGAAAA
Kcnip2_reverse	GTGGCACACCGTGGATAGTT
Pitx2_forward	CCTTACGGAAGCCCGAGT
Pitx2_reverse	AAAGCCATTCTTGCACAGC
Ptk2b_forward	CTCCTCCACAGACCAACCTG
Ptk2b_reverse	AGGAGAGCTGGCACACAGAC
Slc9a3r1_forward	CCCTTCAGCAATGGAGAGATAC
Slc9a3r1_reverse	TGGGGCTCTCTGAAGCTG
Tob1_forward	ACTTTTGCTGCCACCAAGTT
Tob1_reverse	GAGCTACCTTGCTGCTACGG
Unc5a_forward	CGTCATTGAACGCAGCAC
Unc5a_reverse	GATACGTTGATCCGGACCTC
Wnt2b_forward	GGGCCCTCATGAACTTACAC
Wnt2b_reverse	CCACTCACACCGTGACACTT
Hprt1_forward	GACAGGGACTCACTGCATAGTTT
Hprt1_reverse	GAGGCCAAGACAAGAAGACG
Others	Ordered from GeneGlobe, Qiagen (https://www.qiagen.com/us/geneglobe/)

And the following primers were used for ChIP-qPCR (5’ – 3’):

Primer	Sequence (5' - 3')
Adcy1_forward	GTGGCCCTTGCTGCATAC
Adcy1_reverse	CCAGCAGGCTCCTAACACC
Adk_forward	GCGCTCAGTGTCTGATGACTT
Adk_reverse	TGATGCGTCTATTTTTAGGTAGCA
Camk1d_forward	GGAAATGTTAAGTTTTCTTTGTAGCA
Camk1d_reverse	CAATTCAGCCGAACAGCTC
Caprin2_forward	TGCCCCACAGATGATTTTTC
Caprin2_reverse	TGGCGCATACCCTCCTACTA
Ccnd1_forward	GGGGCTTCTTTCCCTAAGAG
Ccnd1_reverse	CCTCCCTCCTAGCTGTCCTC
Ctnnb1_forward	TCCCAGGCTGAAGTCCTTAAT
Ctnnb1_reverse	ACTCTGTCCCAGGCAAGCTA
Dkk1_forward	GCTCCTCAGGGAAGACAACA
Dkk1_reverse	CGAGGGGAGAGTGTCAAAGT
Fuz_forward	CGTGGTTTTCATGATTTTGGA
Fuz_reverse	CCACCAAATGAGATGGTTGTT
Kcnip2_forward	GTGTCACACACGCCTACACC
Kcnip2_reverse	GGGCTGAGGAAGAACAGGT
Pitx2_forward	ACTCCGACCCGAAGAAGG

Pitx2_reverse	TGGTGGCAAGACGTCACTC
Ptk2b_forward	CAGTCCTGCAGAGACAACAGA
Ptk2b_reverse	GATGTGGCATGTGGCTTG
Slc9a3r1_forward	AGACATCCGTCCGGTTCTTA
Slc9a3r1_reverse	TGCTCCCCCACACTCACT
Tob1_forward	CACTCCGGACGATAACTCG
Tob1_reverse	ACTCTAGGGGCGCGGATA
Unc5a_forward	CATCCAGTTTGAATGGAGAGC
Unc5a_reverse	ATGGCTGACATCCACATTCTC
Wnt2b_forward	GGTACCACCGTAGGCAACC
Wnt2b_reverse	GGAGGGAACGTTGAGTCCTT

Co-immunoprecipitation and mass spectrometry (CoIP/MS)

BAF155 and BAF170 interaction analyses were performed using the neural stem cell line, NS5 (Conti et al., 2005), and E13.5 and E17.5 embryonic telencephalic tissue. Tissues were dissected and minced in cold phosphate-buffered saline (PBS) and then washed twice with PBS. Equivalent amounts of cells from one embryo were lysed for 30 min in 1 ml RIPA buffer containing a proteinase inhibitor cocktail (Roche) and DNase. All steps were performed at 4°C. Lysates were centrifuged for 10 min at 13,000 rpm to sediment out non-lysed tissues. The supernatant was pre-cleared by incubating with normal mouse IgG together with protein A/G-agarose beads, as described by the manufacturer (sc-2003; Santa Cruz). Interacting proteins were immunoprecipitated by

incubating pre-cleared supernatant with rabbit anti-BAF155 and anti-BAF170 antibodies and A/G-agarose beads. The beads were then washed first with 500 µl cold RIPA buffer (three times for 5 min each) and then with 40 µl of elution buffer (2.5 µl 20% SDS, 5 µl 1 M $NaHCO_3$, 42.5 µl double-distilled H_2O) for 15 min at room temperature.

For MS analyses (performed in the department of Prof. Dr. Henning Urlaub), samples were suspended in NuPage loading buffer and resolved on commercial SDS polyacrylamide gels (Novex NuPage Bis-Tris gel, 4–12% gradient; Invitrogen). Individual lanes were then cut into six squares for MS analysis. The parameters for the identification of proteins were set to the following values: limit, 95% probability of detection; limit of unique peptides detected, 1; and threshold detection probability of peptides, 80%.

The list of BAF155- and BAF170-interacting proteins revealed by MS analysis was obtained by subtracting nonspecific interactions with IgG in IPs and in telencephalic tissues from BAF155-null (*BAF155cKO_FoxG1*-Cre) and BAF170-null (*BAF170cKO_FoxG1*-Cre) mutants. The first set of controls excludes nonspecific binding to the antibody, and the second excludes nonspecific interactions that possibly could be precipitated by either the anti-BAF155 or anti-BAF170 antibody.

Injection of Wnt inhibitor (WNTi, ICG 001, XAV-939) and Wnt activator (WNTa, SB-216763), H3K27 demethylase inhibitor (GSK-J4), LSD1 histone demethylase (2-PCPA).

ICG001 (Tocris Bioscience, Cat. No. 4505), XAV-939 (Tocris Bioscience, Cat. No. 3748), SB-216763 (Tocris Bioscience, Cat. No. 1616), GSK-J4 (SIGMA, Cat. No. SML0701) and 2-PCPA (Tocris Bioscience, Cat. No. 3852) were dissolved in vehicle

(DMSO). 11.5 d.p.c. pregnant mice received daily injections of vehicle (150 µl), or ICG001 (150 µl of a 1-mg/ml solution), XAV-939, (150 µl of a 0.2 mg/ml solution), SB-216763 (100 µl of a 1-mg/ml solution plus 100 µl of saline), GSK-J4 (150µl of 2.5mg/ml solution)) and 2-PCPA (150µl of 5 mg/ml solution). Treated mice were sacrificed at different developmental stages as indicated in the text.

In vivo β-catenin transcriptional activity assay

In vivo and in vitro β-catenin transcriptional activity assay were performed as previously described (Durak et al., 2016; Mao et al., 2009). Briefly, Wnt/β-catenin transcriptional activity in vivo was monitored by electroporating brains of E13.5 *BAF155*$^{fl/fl}$;*BAF170*$^{fl/fl}$ embryos with a Cre plasmid (or empty plasmid as a control) and the reporter constructs Super8XTOPFLASH (TOP) or Super8XFOPFLASH (FOP) together with pRL-TK constructs at a 5:1:0.3 ratio. Wnt/β-catenin transcriptional activity was measured at E15.5. For in vitro assay, Neuro2A cells at 1×10^5 per well density were plated into 24-well plates. Cells were transfected with 0.8 µg of shRNA plasmids (shBAF155, shBAF170) along with 50 ng of Super8XTOPFLASH and 10 ng of pRL-TK. 2 days post-transfection, cells were collected for Wnt/β-catenin transcriptional activity measurement. In all cases, firefly luciferase activity was normalized to that of Renilla luciferase.

Demethylase assays

Cell culture-based demethylase activity assay for Utx/KDM6A and Jmjd3/KDM6B was performed previously (Narayanan et al., 2015) .

For Histone demethylase KDM1/LSD1 activity quantification, cultured dcKO_CAG-Cre NSCs were nucleofected with a mammalian expression vector for LSD1/KDM1A. The Cre-mediated deletion of BAF155 and BAF170 alleles was induced by adding TAM to the growth medium at a final concentration of 1 µM. After 2 DIV, The LSD1 demethylation assay was performed by using nuclear extracts from above NSCs and Epigenase LSD1 Demethylase activity Kit according to manufacturer (Epigentek)'s recommendations (Katz et al., 2014).

IF and cell cycle parameters

IF experiments and determination of cell cycle indexes were carried out as previously described (Narayanan et al., 2015; Tuoc and Stoykova, 2008).

Spindle angle analysis

Brain sections were stained with PVIM to outline the cell shape and PHH3 to identify anaphase and early telophase dividing cells. Images of z-stack sections were obtained by SP5 confocal microscopy, and 3D reconstruction of the confocal stacks was done as described previously (Postiglione et al., 2011; Tuoc et al., 2013).

3D reconstruction and cell counting

3D images of the developing hippocampus were constructed using Neurolucida software version 11.03. Consecutive sections (25 µm each) of the E15.5 WT and dcKO hippocampus were imaged in rostro-caudal order. Triple IF with antibodies against ZBTB20, PAX6 and PHH3 was performed to quantify the volume of the hippocampal plate (ZBTB20^{+}/PAX6^{-}), hippocampal neuroepithelium (ZBTB20^{-}/PAX6^{+}), and total

number of pHH3$^+$ M-phase cells in the apical surface of the developing hippocampus. Contours were drawn in each section based on the expression of the hippocampus-specific marker Ztbt20. The 3D reconstruction was produced from whole-stack contours. The volume analysis was done using Neurolucida Explorer v. 11.03.

Cell counts and quantitative analysis of IF signal intensity

Immunostaining in IF images was quantified using anatomically matched forebrain sections. Nucleus-marker–positive cells within the pallium of confocal images were counted for comparison. In most cases, cell counts of six matched sections were averaged from three biological replicates (control/dcKO pallium). In many cases, the number of lineage marker cells was quantified using total marker-positive cells alone, or by normalizing to the total number of DAPI$^+$ (nucleus-stained) cells using the following equation: Normalized number = marker-positive cell number/DAPI$^+$ cell number. Statistical analyses of histological data were performed using Student's *t*-test. All bar graphs are plotted as means ± SEM. All statistical tests are two-tailed, and *P*-values are considered to be significant for $\alpha = 0.05$. All details of statistical analyses of histological experiments are presented in Table S6.

Statistical Analysis

Statistical analyses were designed using the assumption of normal distribution and similar variance among groups, as previously tested. The sample size was determined based on preliminary results or similar experiments carried-out in the past. Power analysis was performed using G-power in order to estimate the number of animals required, for a signal-to-noise ratio of 1.4 and 80% to 90% power assuming a 5%

significance level. For histological analysis, qPCR, ChIP-qPCR and comparison of genome-wide histone marks, statistics were assessed with Student's *t*-test. The significance of overlaps was assessed using hypergeometric test. RNA-Seq and ChIP-Seq analyses were carried out using DESeq2 and DiffBind packages of Bioconductor respectively, with their own in-built statistical calculation tools. The results are presented as means ± SEM. *P* values of < 0.05 were considered to be statistically significant unless otherwise indicated. All the relevant information pertaining to statistical analysis is also specified in each figure legend separately. A detailed description of quantitative analysis methods is presented in Supplemental Information. The statistic quantification was carried out as average from at least three biological replicate. All details of statistical analyses for histological experiments are presented in Table S6.

SUPPLEMENTAL REFERENCES

Chen, J., Bardes, E.E., Aronow, B.J., and Jegga, A.G. (2009). ToppGene Suite for gene list enrichment analysis and candidate gene prioritization. Nucleic acids research *37*, W305-311.

Choi, J., Ko, M., Jeon, S., Jeon, Y., Park, K., Lee, C., Lee, H., and Seong, R.H. (2012). The SWI/SNF-like BAF complex is essential for early B cell development. J Immunol *188*, 3791-3803.

Conti, L., Pollard, S.M., Gorba, T., Reitano, E., Toselli, M., Biella, G., Sun, Y., Sanzone, S., Ying, Q.L., Cattaneo, E.*, et al.* (2005). Niche-independent symmetrical self-renewal of a mammalian tissue stem cell. PLoS Biol *3*, e283.

De Santa, F., Totaro, M.G., Prosperini, E., Notarbartolo, S., Testa, G., and Natoli, G. (2007). The histone H3 lysine-27 demethylase Jmjd3 links inflammation to inhibition of polycomb-mediated gene silencing. Cell *130*, 1083-1094.

Djebali, S., Davis, C.A., Merkel, A., Dobin, A., Lassmann, T., Mortazavi, A., Tanzer, A., Lagarde, J., Lin, W., and Schlesinger, F. (2012). Landscape of transcription in human cells. Nature *489*, 101-108.

Durak, O., Gao, F., Kaeser-Woo, Y.J., Rueda, R., Martorell, A.J., Nott, A., Liu, C.Y., Watson, L.A., and Tsai, L.H. (2016). Chd8 mediates cortical neurogenesis via transcriptional regulation of cell cycle and Wnt signaling. Nat Neurosci.

Feng, J., Liu, T., Qin, B., Zhang, Y., and Liu, X.S. (2012). Identifying ChIP-seq enrichment using MACS. Nature protocols *7*, 1728-1740.

Goebbels, S., Bormuth, I., Bode, U., Hermanson, O., Schwab, M.H., and Nave, K.A. (2006). Genetic targeting of principal neurons in neocortex and hippocampus of NEX-Cre mice. Genesis *44*, 611-621.

Gorski, J.A., Talley, T., Qiu, M., Puelles, L., Rubenstein, J.L., and Jones, K.R. (2002). Cortical excitatory neurons and glia, but not GABAergic neurons, are produced in the Emx1-expressing lineage. J Neurosci *22*, 6309-6314.

Halder, R., Hennion, M., Vidal, R.O., Shomroni, O., Rahman, R.-U., Rajput, A., Centeno, T.P., van Bebber, F., Capece, V., and Vizcaino, J.C.G. (2016). DNA methylation changes in plasticity genes accompany the formation and maintenance of memory. Nature neuroscience *19*, 102-110.

Heinz, S., Benner, C., Spann, N., Bertolino, E., Lin, Y.C., Laslo, P., Cheng, J.X., Murre, C., Singh, H., and Glass, C.K. (2010). Simple combinations of lineage-determining transcription factors prime cis-regulatory elements required for macrophage and B cell identities. Molecular cell *38*, 576-589.

Katz, T.A., Vasilatos, S.N., Harrington, E., Oesterreich, S., Davidson, N.E., and Huang, Y. (2014). Inhibition of histone demethylase, LSD2 (KDM1B), attenuates DNA methylation and increases sensitivity to DNMT inhibitor-induced apoptosis in breast cancer cells. Breast Cancer Res Treat *146*, 99-108.

Li, H., Handsaker, B., Wysoker, A., Fennell, T., Ruan, J., Homer, N., Marth, G., Abecasis, G., and Durbin, R. (2009). The sequence alignment/map format and SAMtools. Bioinformatics *25*, 2078-2079.

Lienhard, M., Grimm, C., Morkel, M., Herwig, R., and Chavez, L. (2014). MEDIPS: genome-wide differential coverage analysis of sequencing data derived from DNA enrichment experiments. Bioinformatics *30*, 284-286.

Love, M.I., Huber, W., and Anders, S. (2014). Moderated estimation of fold change and dispersion for RNA-seq data with DESeq2. Genome biology *15*, 1.

Madisen, L., Zwingman, T.A., Sunkin, S.M., Oh, S.W., Zariwala, H.A., Gu, H., Ng, L.L., Palmiter, R.D., Hawrylycz, M.J., Jones, A.R.*, et al.* (2010). A robust and high-throughput Cre reporting and characterization system for the whole mouse brain. Nat Neurosci *13*, 133-140.

Mao, Y., Ge, X., Frank, C.L., Madison, J.M., Koehler, A.N., Doud, M.K., Tassa, C., Berry, E.M., Soda, T., Singh, K.K.*, et al.* (2009). Disrupted in schizophrenia 1 regulates neuronal progenitor proliferation via modulation of GSK3beta/beta-catenin signaling. Cell *136*, 1017-1031.

Narayanan, R., Pirouz, M., Kerimoglu, C., Pham, L., Wagener, R.J., Kiszka, K.A., Rosenbusch, J., Seong, R.H., Kessel, M., Fischer, A.*, et al.* (2015). Loss of BAF (mSWI/SNF) Complexes Causes Global Transcriptional and Chromatin State Changes in Forebrain Development. Cell Rep *13*, 1842-1854.

Nicol, J.W., Helt, G.A., Blanchard, S.G., Raja, A., and Loraine, A.E. (2009). The Integrated Genome Browser: free software for distribution and exploration of genome-scale datasets. Bioinformatics *25*, 2730-2731.

Postiglione, M.P., Juschke, C., Xie, Y., Haas, G.A., Charalambous, C., and Knoblich, J.A. (2011). Mouse inscuteable induces apical-Basal spindle orientation to facilitate intermediate progenitor generation in the developing neocortex. Neuron *72*, 269-284.

Ross-Innes, C.S., Stark, R., Teschendorff, A.E., Holmes, K.A., Ali, H.R., Dunning, M.J., Brown, G.D., Gojis, O., Ellis, I.O., and Green, A.R. (2012). Differential oestrogen receptor binding is associated with clinical outcome in breast cancer. Nature *481*, 389-393.

Shen, L., Shao, N., Liu, X., and Nestler, E. (2014). ngs. plot: Quick mining and visualization of next-generation sequencing data by integrating genomic databases. BMC genomics *15*, 1.

Tuoc, T.C., Boretius, S., Sansom, S.N., Pitulescu, M.E., Frahm, J., Livesey, F.J., and Stoykova, A. (2013). Chromatin Regulation by BAF170 Controls Cerebral Cortical Size and Thickness. Developmental Cell *25*, 256-269.

Tuoc, T.C., and Stoykova, A. (2008). Trim11 modulates the function of neurogenic transcription factor Pax6 through ubiquitin-proteosome system. Genes & development *22*, 1972-1986.

Veeman, M.T., Slusarski, D.C., Kaykas, A., Louie, S.H., and Moon, R.T. (2003). Zebrafish prickle, a modulator of noncanonical Wnt/Fz signaling, regulates gastrulation movements. Current biology : CB *13*, 680-685.

Zhuo, L., Theis, M., Alvarez-Maya, I., Brenner, M., Willecke, K., and Messing, A. (2001). hGFAP-cre transgenic mice for manipulation of glial and neuronal function in vivo. Genesis *31*, 85-94.
