# Supplementary material for: Epigenetic Regulation by BAF Complexes Limits Neural Stem Cell Proliferation by Suppressing Wnt Signaling in Late Embryonic Development
Source: Stem Cell Reports. 2018 May 17;10(6):1734–50. doi: 10.1016/j.stemcr.2018.04.014 (PMC5993560; doi:10.1016/j.stemcr.2018.04.014)
Supplement: Table S3. Lists of Each of Those Gene Groups (Downregulated Neurogenesis Genes, Neurogenesis Genes with High H3K27me3, Upregulated Proliferation Genes, Cell-Cycle Genes with High H3K4me2, Upregulated Wnt, Wnt Target Genes) Regulated by BAF155/BAF170 in the dcKO_hGFAP-Cre Pallium at E17.5, Related to [file mmc4.docx]

**Table S8. Function of BAF complexes – dependent genes**

| **Gene** | **Expression in dcKO** | **Mutant** | **Phenotype** | **Reference** |
| --- | --- | --- | --- | --- |
| **Hippocampal development** | | | | |
| Zbtb20 | Downregulated | KO | Hippocampal malformation | ^1, 2^ |
| MAPT | Downregulated | KO/KI | Hippocampal malformation | ^3^ |
| Ncor2/SMRT | Downregulated | KO | Hippocampal malformation | ^4^ |
| Kif1b | Downregulated | KO | Hippocampal malformation | ^5^ |
| **Cortical layer formation** | | | | |
| ADCY1 | Downregulated | KO | Cortical layer defect | ^6^ |
| APC | Downregulated | KO | Cortical layer defect | ^7^ |
| Dcx | Downregulated | KO | Cortical layer defect | ^8^ |
| KLF7 | Downregulated | KO | Malformations of the nervous system | ^9^ |
| L1CAM | Downregulated | KO | Malformations of the nervous system | ^10^ |
| MAP1B | Downregulated | KO | Malformations of the nervous system | ^11^ |
| Rybp | Downregulated | KO | Malformations of the nervous system | ^12^ |
| Unc5d | Downregulated | KO | Cortical layer defect | ^13^ |
| Zbtb20 | Downregulated | KO | Cortical layer defect | ^14^ |
| Lmo4 | Downregulated | KO | Cortical layer defect | 15, 16 |
| Ncor2/SMRT | Downregulated | KO | Cortical layer defect | ^4^ |
| Kif1b | Downregulated | KO | Malformations of the nervous system | ^5^ |
| **Proliferation** | | | | |
| MAPT | Downregulated | KO/KI | Increased proliferation | ^3^ |
| Trnp1 | Downregulated | KD(OE) | Altered proliferation | ^17^ |
| Lmo4 | Downregulated | KO | Increased proliferation | 18 |
| Pax6 | upregulated | OE | Positively control of proliferation | 19 |
| Ctnnb1/  β-Catintin | upregulated | OE | Positively control of proliferation | 20 |
| Axin2 | upregulated | OE | Positively control of proliferation | 20 |
| Shh signalling (Evc, Cc2d2a, Ift80, Sfrp1, Fuz, Shox2, Gli1, Gli3 Cdon)  Notch (Notch3, Dll1, Hes5, Lfng, Jag1, Tle1) | | | | |
| Gli3 | upregulated | KO | Positively control of proliferation | 21-23 |
| EPHB1 | upregulated | KO | Positively control of proliferation | 24 |
| TFAP2C/ AP2γ | upregulated | KO | Positively control of proliferation | 25 |
| TFAP2A | upregulated |  |  |  |
| Nr2e1/Tlx | upregulated | KO, OE | Positively control of proliferation | ^26-29^ |
| Cdc45 | upregulated | KO | Positively control of proliferation | ^30^ |
| E2f1 | upregulated | KO | Positively control of proliferation | ^31, 32^ |
| E2f5 | upregulated | KO | Positively control of proliferation | ^33^ |
| Myc | upregulated | KO | Positively control of proliferation | ^34, 35^ |
| Lfng(Notch) | upregulated | KO | Positively control of proliferation | ^36, 37^ |
| **Differentiation** | | | | |
| MAPT | Downregulated | KO/KI | Decreased differentiation | 3 |
| Lmo4 | Downregulated | KO | Decreased differentiation | 15 |
| **Genes involved in orientation of the mitotic spindle** | | | | |
| KIF5A | Downregulated |  |  | ^38, 39^ |
| KIF5C | Downregulated |  |  | ^38, 39^ |
| MAPRE2 | Downregulated |  |  | ^40, 41^ |
| APC | Downregulated |  |  | ^42^ |
| STARD9 | Downregulated |  |  | ^43-45^ |
| PRPH | Downregulated |  |  | ^38, 39^ |
| CKAP5 | Downregulated |  |  | ^46^ |
| CD34 | Downregulated |  |  | ^46^ |
| CALM1 | Downregulated |  |  | ^46^ |
| PRKCB | Downregulated |  |  | ^46^ |
| **Neuroepithelial cells (tight junction genes)** | | | | |
| occludin | upregulated |  |  | ^47, 48^ |
| AMOT | Upregulated |  |  | ^49-57^ |
| MPP5 | upregulated |  |  | ^54, 55, 58-60^ |
| INADL | Upregulated |  |  | ^47, 54, 60-68^ |
| PKD2 | upregulated |  |  | ^69-71^ |
| DLG1 | upregulated |  |  | ^72-74^ |
| CFTR | upregulated |  |  | ^75-80^ |
| TGFB3 | upregulated |  |  | ^81-84^ |
| **Radial glial progenitors (astroglial, adherens junctions genes)** | | | | |
| GLAST | Downregulated | astroglial |  | ^85^ |
| BLBP | Downregulated | astroglial |  | ^85^ |
| SOX8 | Downregulated | astroglial |  | ^86^ |
| HEPACAM | Downregulated | astroglial |  | ^87, 88^ |
| SPARCL1 | Downregulated | astroglial |  | ^89-91^ |
| BCAN | Downregulated | astroglial |  | ^92-96^ |
| AQP4 | Downregulated | astroglial |  | ^97, 98^ |
| ATP1A2 | Downregulated | astroglial |  | ^99-101^ |
| SPARC | Downregulated | astroglial |  | ^102-106^ |
| ZO1 | Downregulated | adherens junctions |  | ^48^ |
| α-Catenin | Downregulated | adherens junctions |  | ^107, 108^ |
| PTK2B | Downregulated | adherens junctions |  | ^109^ |
| PLEC | Downregulated | adherens junctions |  | ^110, 111^ |
| TNS3 | Downregulated | adherens junctions |  | ^112^ |
| KIAA1462/ Jcad | Downregulated | adherens junctions |  | ^112^ |
| filamin | Downregulated | adherens junctions |  | ^113, 114^ |
| CAMSAP3 | Downregulated | adherens junctions |  | ^115^ |
| APC | Downregulated | adherens junctions |  | ^112, 116^ |
| MYH9 | Downregulated | adherens junctions |  | ^117-119^ |
| MYO1E | Downregulated | adherens junctions |  | ^120, 121^ |
| SPTAN1 | Downregulated | adherens junctions |  | ^122-124^ |
| ITGA1 | Downregulated | adherens junctions |  | ^125^ |
| RND1 | Downregulated | adherens junctions |  | ^126^ |

References

1. Sutherland, A.P. *et al.* Zinc finger protein Zbtb20 is essential for postnatal survival and glucose homeostasis. *Molecular and cellular biology* **29**, 2804-2815 (2009).

2. Rosenthal, E.H., Tonchev, A.B., Stoykova, A. & Chowdhury, K. Regulation of archicortical arealization by the transcription factor Zbtb20. *Hippocampus* **22**, 2144-2156 (2012).

3. Sennvik, K. *et al.* Tau-4R suppresses proliferation and promotes neuronal differentiation in the hippocampus of tau knockin/knockout mice. *FASEB journal : official publication of the Federation of American Societies for Experimental Biology* **21**, 2149-2161 (2007).

4. Jepsen, K. *et al.* SMRT-mediated repression of an H3K27 demethylase in progression from neural stem cell to neuron. *Nature* **450**, 415-419 (2007).

5. Zhao, C. *et al.* Charcot-Marie-Tooth disease type 2A caused by mutation in a microtubule motor KIF1Bbeta. *Cell* **105**, 587-597 (2001).

6. Abdel-Majid, R.M. *et al.* Loss of adenylyl cyclase I activity disrupts patterning of mouse somatosensory cortex. *Nat Genet* **19**, 289-291 (1998).

7. Yokota, Y. *et al.* The adenomatous polyposis coli protein is an essential regulator of radial glial polarity and construction of the cerebral cortex. *Neuron* **61**, 42-56 (2009).

8. Pilz, D.T. *et al.* LIS1 and XLIS (DCX) mutations cause most classical lissencephaly, but different patterns of malformation. *Hum Mol Genet* **7**, 2029-2037 (1998).

9. Laub, F. *et al.* Transcription factor KLF7 is important for neuronal morphogenesis in selected regions of the nervous system. *Mol Cell Biol* **25**, 5699-5711 (2005).

10. Dahme, M. *et al.* Disruption of the mouse L1 gene leads to malformations of the nervous system. *Nat Genet* **17**, 346-349 (1997).

11. Meixner, A. *et al.* MAP1B is required for axon guidance and Is involved in the development of the central and peripheral nervous system. *J Cell Biol* **151**, 1169-1178 (2000).

12. Pirity, M.K., Locker, J. & Schreiber-Agus, N. Rybp/DEDAF is required for early postimplantation and for central nervous system development. *Mol Cell Biol* **25**, 7193-7202 (2005).

13. Yamagishi, S. *et al.* FLRT2 and FLRT3 act as repulsive guidance cues for Unc5-positive neurons. *EMBO J* **30**, 2920-2933 (2011).

14. Tonchev, A.B., Tuoc, T.C., Rosenthal, E.H., Studer, M. & Stoykova, A. Zbtb20 modulates the sequential generation of neuronal layers in developing cortex. *Mol Brain* **9**, 65 (2016).

15. Asprer, J.S. *et al.* LMO4 functions as a co-activator of neurogenin 2 in the developing cortex. *Development* **138**, 2823-2832 (2011).

16. Cederquist, G.Y., Azim, E., Shnider, S.J., Padmanabhan, H. & Macklis, J.D. Lmo4 establishes rostral motor cortex projection neuron subtype diversity. *J Neurosci* **33**, 6321-6332 (2013).

17. Stahl, R. *et al.* Trnp1 regulates expansion and folding of the mammalian cerebral cortex by control of radial glial fate. *Cell* **153**, 535-549 (2013).

18. Lee, S.K. *et al.* The LIM domain-only protein LMO4 is required for neural tube closure. *Mol Cell Neurosci* **28**, 205-214 (2005).

19. Wong, F.K. *et al.* Sustained Pax6 Expression Generates Primate-like Basal Radial Glia in Developing Mouse Neocortex. *Plos Biology* **13**, e1002217 (2015).

20. Chenn, A. & Walsh, C.A. Regulation of cerebral cortical size by control of cell cycle exit in neural precursors. *Science* **297**, 365-369 (2002).

21. Wang, H., Ge, G.N., Uchida, Y., Luu, B. & Ahn, S. Gli3 Is Required for Maintenance and Fate Specification of Cortical Progenitors. *Journal of Neuroscience* **31**, 6440-6448 (2011).

22. Zhan, X., Shi, X., Zhang, Z., Chen, Y. & Wu, J.I. Dual role of Brg chromatin remodeling factor in Sonic hedgehog signaling during neural development. *Proc Natl Acad Sci U S A* **108**, 12758-12763 (2011).

23. Petrova, R., Garcia, A.D.R. & Joyner, A.L. Titration of GLI3 Repressor Activity by Sonic Hedgehog Signaling Is Critical for Maintaining Multiple Adult Neural Stem Cell and Astrocyte Functions. *Journal of Neuroscience* **33**, 17490-17505 (2013).

24. Chumley, M.J., Catchpole, T., Silvany, R.E., Kernie, S.G. & Henkemeyer, M. EphB receptors regulate stem/progenitor cell proliferation, migration, and polarity during hippocampal neurogenesis. *J Neurosci* **27**, 13481-13490 (2007).

25. Pinto, L. *et al.* AP2gamma regulates basal progenitor fate in a region- and layer-specific manner in the developing cortex. *Nat Neurosci* **12**, 1229-1237 (2009).

26. Murai, K. *et al.* The TLX-miR-219 cascade regulates neural stem cell proliferation in neurodevelopment and schizophrenia iPSC model. *Nat Commun* **7**, 10965 (2016).

27. Niu, W., Zou, Y., Shen, C. & Zhang, C.L. Activation of postnatal neural stem cells requires nuclear receptor TLX. *J Neurosci* **31**, 13816-13828 (2011).

28. Zhang, C.L., Zou, Y., He, W., Gage, F.H. & Evans, R.M. A role for adult TLX-positive neural stem cells in learning and behaviour. *Nature* **451**, 1004-1007 (2008).

29. Roy, K. *et al.* The Tlx gene regulates the timing of neurogenesis in the cortex. *J Neurosci* **24**, 8333-8345 (2004).

30. Blumroder, R. *et al.* Mcm3 replicative helicase mutation impairs neuroblast proliferation and memory in Drosophila. *Genes, brain, and behavior* **15**, 647-659 (2016).

31. Cooper-Kuhn, C.M. *et al.* Impaired adult neurogenesis in mice lacking the transcription factor E2F1. *Mol Cell Neurosci* **21**, 312-323 (2002).

32. Palm, T. *et al.* A systemic transcriptome analysis reveals the regulation of neural stem cell maintenance by an E2F1-miRNA feedback loop. *Nucleic Acids Res* **41**, 3699-3712 (2013).

33. Vied, C.M. *et al.* A multi-resource data integration approach: identification of candidate genes regulating cell proliferation during neocortical development. *Frontiers in neuroscience* **8**, 257 (2014).

34. Knoepfler, P.S., Cheng, P.F. & Eisenman, R.N. N-myc is essential during neurogenesis for the rapid expansion of progenitor cell populations and the inhibition of neuronal differentiation. *Genes Dev* **16**, 2699-2712 (2002).

35. Zhao, X. *et al.* The N-Myc-DLL3 cascade is suppressed by the ubiquitin ligase Huwe1 to inhibit proliferation and promote neurogenesis in the developing brain. *Dev Cell* **17**, 210-221 (2009).

36. Semerci, F. *et al.* Lunatic fringe-mediated Notch signaling regulates adult hippocampal neural stem cell maintenance. *Elife* **6** (2017).

37. Nikolaou, N. *et al.* Lunatic fringe promotes the lateral inhibition of neurogenesis. *Development* **136**, 2523-2533 (2009).

38. Pfeffer, T.J. *et al.* The natural diterpene tonantzitlolone A and its synthetic enantiomer inhibit cell proliferation and kinesin-5 function. *Eur J Med Chem* **112**, 164-170 (2016).

39. Ferenz, N.P., Gable, A. & Wadsworth, P. Mitotic functions of kinesin-5. *Semin Cell Dev Biol* **21**, 255-259 (2010).

40. Su, L.K. & Qi, Y. Characterization of human MAPRE genes and their proteins. *Genomics* **71**, 142-149 (2001).

41. Ning, W. *et al.* The CAMSAP3-ACF7 Complex Couples Noncentrosomal Microtubules with Actin Filaments to Coordinate Their Dynamics. *Dev Cell* **39**, 61-74 (2016).

42. Yumoto, T. *et al.* Radmis, a novel mitotic spindle protein that functions in cell division of neural progenitors. *PLoS One* **8**, e79895 (2013).

43. Torres, J.Z. *et al.* The STARD9/Kif16a kinesin associates with mitotic microtubules and regulates spindle pole assembly. *Cell* **147**, 1309-1323 (2011).

44. Torres, J.Z. STARD9/Kif16a is a novel mitotic kinesin and antimitotic target. *Bioarchitecture* **2**, 19-22 (2012).

45. Okamoto, N. *et al.* A novel genetic syndrome with STARD9 mutation and abnormal spindle morphology. *Am J Med Genet A* (2017).

46. Booth, D.G., Hood, F.E., Prior, I.A. & Royle, S.J. A TACC3/ch-TOG/clathrin complex stabilises kinetochore fibres by inter-microtubule bridging. *EMBO J* **30**, 906-919 (2011).

47. Du, D. *et al.* The tight junction protein, occludin, regulates the directional migration of epithelial cells. *Dev Cell* **18**, 52-63 (2010).

48. Aaku-Saraste, E., Hellwig, A. & Huttner, W.B. Loss of occludin and functional tight junctions, but not ZO-1, during neural tube closure--remodeling of the neuroepithelium prior to neurogenesis. *Developmental biology* **180**, 664-679 (1996).

49. Campbell, C.I. *et al.* The RNF146 and tankyrase pathway maintains the junctional Crumbs complex through regulation of angiomotin. *J Cell Sci* **129**, 3396-3411 (2016).

50. Citi, S., Guerrera, D., Spadaro, D. & Shah, J. Epithelial junctions and Rho family GTPases: the zonular signalosome. *Small GTPases* **5**, 1-15 (2014).

51. Hultin, S. *et al.* AmotL2 integrates polarity and junctional cues to modulate cell shape. *Sci Rep* **7**, 7548 (2017).

52. Mack, N.A. & Georgiou, M. The interdependence of the Rho GTPases and apicobasal cell polarity. *Small GTPases* **5**, 10 (2014).

53. Shimada, H. *et al.* Loss of tricellular tight junction protein LSR promotes cell invasion and migration via upregulation of TEAD1/AREG in human endometrial cancer. *Sci Rep* **7**, 37049 (2017).

54. Wells, C.D. *et al.* A Rich1/Amot complex regulates the Cdc42 GTPase and apical-polarity proteins in epithelial cells. *Cell* **125**, 535-548 (2006).

55. Yi, C. *et al.* A tight junction-associated Merlin-angiomotin complex mediates Merlin's regulation of mitogenic signaling and tumor suppressive functions. *Cancer Cell* **19**, 527-540 (2011).

56. Zheng, Y. *et al.* Angiomotin-like protein 1 controls endothelial polarity and junction stability during sprouting angiogenesis. *Circ Res* **105**, 260-270 (2009).

57. Bratt, A. *et al.* Angiomotin regulates endothelial cell-cell junctions and cell motility. *J Biol Chem* **280**, 34859-34869 (2005).

58. Ebnet, K., Iden, S., Gerke, V. & Suzuki, A. Regulation of epithelial and endothelial junctions by PAR proteins. *Front Biosci* **13**, 6520-6536 (2008).

59. Gosens, I. *et al.* FERM protein EPB41L5 is a novel member of the mammalian CRB-MPP5 polarity complex. *Exp Cell Res* **313**, 3959-3970 (2007).

60. Michel, D. *et al.* PATJ connects and stabilizes apical and lateral components of tight junctions in human intestinal cells. *J Cell Sci* **118**, 4049-4057 (2005).

61. Adachi, M. *et al.* Similar and distinct properties of MUPP1 and Patj, two homologous PDZ domain-containing tight-junction proteins. *Mol Cell Biol* **29**, 2372-2389 (2009).

62. Shin, K., Straight, S. & Margolis, B. PATJ regulates tight junction formation and polarity in mammalian epithelial cells. *J Cell Biol* **168**, 705-711 (2005).

63. Straight, S.W. *et al.* Loss of PALS1 expression leads to tight junction and polarity defects. *Mol Biol Cell* **15**, 1981-1990 (2004).

64. Roh, M.H., Fan, S., Liu, C.J. & Margolis, B. The Crumbs3-Pals1 complex participates in the establishment of polarity in mammalian epithelial cells. *J Cell Sci* **116**, 2895-2906 (2003).

65. Hurd, T.W., Gao, L., Roh, M.H., Macara, I.G. & Margolis, B. Direct interaction of two polarity complexes implicated in epithelial tight junction assembly. *Nat Cell Biol* **5**, 137-142 (2003).

66. Poliak, S., Matlis, S., Ullmer, C., Scherer, S.S. & Peles, E. Distinct claudins and associated PDZ proteins form different autotypic tight junctions in myelinating Schwann cells. *J Cell Biol* **159**, 361-372 (2002).

67. Roh, M.H., Liu, C.J., Laurinec, S. & Margolis, B. The carboxyl terminus of zona occludens-3 binds and recruits a mammalian homologue of discs lost to tight junctions. *J Biol Chem* **277**, 27501-27509 (2002).

68. Lemmers, C. *et al.* hINADl/PATJ, a homolog of discs lost, interacts with crumbs and localizes to tight junctions in human epithelial cells. *J Biol Chem* **277**, 25408-25415 (2002).

69. Duning, K. *et al.* Polycystin-2 activity is controlled by transcriptional coactivator with PDZ binding motif and PALS1-associated tight junction protein. *J Biol Chem* **285**, 33584-33588 (2010).

70. Yu, A.S., Kanzawa, S.A., Usorov, A., Lantinga-van Leeuwen, I.S. & Peters, D.J. Tight junction composition is altered in the epithelium of polycystic kidneys. *The Journal of pathology* **216**, 120-128 (2008).

71. Lee, D.B., Huang, E. & Ward, H.J. Tight junction biology and kidney dysfunction. *American journal of physiology. Renal physiology* **290**, F20-34 (2006).

72. Su, W., Wong, E.W., Mruk, D.D. & Cheng, C.Y. The Scribble/Lgl/Dlg polarity protein complex is a regulator of blood-testis barrier dynamics and spermatid polarity during spermatogenesis. *Endocrinology* **153**, 6041-6053 (2012).

73. Golebiewski, L., Liu, H., Javier, R.T. & Rice, A.P. The avian influenza virus NS1 ESEV PDZ binding motif associates with Dlg1 and Scribble to disrupt cellular tight junctions. *J Virol* **85**, 10639-10648 (2011).

74. Stucke, V.M., Timmerman, E., Vandekerckhove, J., Gevaert, K. & Hall, A. The MAGUK protein MPP7 binds to the polarity protein hDlg1 and facilitates epithelial tight junction formation. *Mol Biol Cell* **18**, 1744-1755 (2007).

75. Castellani, S. *et al.* Emerging relationship between CFTR, actin and tight junction organization in cystic fibrosis airway epithelium. *Histology and histopathology* **32**, 445-459 (2017).

76. Molina, S.A. *et al.* Junctional abnormalities in human airway epithelial cells expressing F508del CFTR. *American journal of physiology. Lung cellular and molecular physiology* **309**, L475-487 (2015).

77. Ruan, Y.C. *et al.* CFTR interacts with ZO-1 to regulate tight junction assembly and epithelial differentiation through the ZONAB pathway. *J Cell Sci* **127**, 4396-4408 (2014).

78. De Lisle, R.C. Disrupted tight junctions in the small intestine of cystic fibrosis mice. *Cell Tissue Res* **355**, 131-142 (2014).

79. Castellani, S. *et al.* NHERF1 and CFTR restore tight junction organisation and function in cystic fibrosis airway epithelial cells: role of ezrin and the RhoA/ROCK pathway. *Lab Invest* **92**, 1527-1540 (2012).

80. Chen, J. *et al.* Cryptorchidism-induced CFTR down-regulation results in disruption of testicular tight junctions through up-regulation of NF-kappaB/COX-2/PGE2. *Human reproduction* **27**, 2585-2597 (2012).

81. Ye, P. Modulation of epithelial tight junctions by TGF-beta 3 in cultured oral epithelial cells. *Australian dental journal* **57**, 11-17 (2012).

82. Xia, W. & Cheng, C.Y. TGF-beta3 regulates anchoring junction dynamics in the seminiferous epithelium of the rat testis via the Ras/ERK signaling pathway: An in vivo study. *Dev Biol* **280**, 321-343 (2005).

83. Lui, W.Y., Lee, W.M. & Cheng, C.Y. TGF-betas: their role in testicular function and Sertoli cell tight junction dynamics. *International journal of andrology* **26**, 147-160 (2003).

84. Lui, W.Y., Lee, W.M. & Cheng, C.Y. Transforming growth factor beta3 regulates the dynamics of Sertoli cell tight junctions via the p38 mitogen-activated protein kinase pathway. *Biology of reproduction* **68**, 1597-1612 (2003).

85. Hartfuss, E., Galli, R., Heins, N. & Gotz, M. Characterization of CNS precursor subtypes and radial glia. *Dev Biol.* **229**, 15-30. (2001).

86. Kordes, U., Cheng, Y.C. & Scotting, P.J. Sox group E gene expression distinguishes different types and maturational stages of glial cells in developing chick and mouse. *Brain Res Dev Brain Res* **157**, 209-213 (2005).

87. Sirisi, S. *et al.* Megalencephalic leukoencephalopathy with subcortical cysts protein 1 regulates glial surface localization of GLIALCAM from fish to humans. *Hum Mol Genet* **23**, 5069-5086 (2014).

88. Favre-Kontula, L. *et al.* GlialCAM, an immunoglobulin-like cell adhesion molecule is expressed in glial cells of the central nervous system. *Glia* **56**, 633-645 (2008).

89. Kucukdereli, H. *et al.* Control of excitatory CNS synaptogenesis by astrocyte-secreted proteins Hevin and SPARC. *Proc Natl Acad Sci U S A* **108**, E440-449 (2011).

90. Weimer, J.M. *et al.* A BAC transgenic mouse model to analyze the function of astroglial SPARCL1 (SC1) in the central nervous system. *Glia* **56**, 935-941 (2008).

91. Lively, S. & Brown, I.R. Analysis of the extracellular matrix protein SC1 during reactive gliosis in the rat lithium-pilocarpine seizure model. *Brain Res* **1163**, 1-9 (2007).

92. Han, D. *et al.* Global transcriptome profiling of genes that are differentially regulated during differentiation of mouse embryonic neural stem cells into astrocytes. *Journal of molecular neuroscience : MN* **55**, 109-125 (2015).

93. Thon, N. *et al.* The chondroitin sulphate proteoglycan brevican is upregulated by astrocytes after entorhinal cortex lesions in adult rats. *Eur J Neurosci* **12**, 2547-2558 (2000).

94. McKeon, R.J., Jurynec, M.J. & Buck, C.R. The chondroitin sulfate proteoglycans neurocan and phosphacan are expressed by reactive astrocytes in the chronic CNS glial scar. *J Neurosci* **19**, 10778-10788 (1999).

95. Yamada, H. *et al.* The brain chondroitin sulfate proteoglycan brevican associates with astrocytes ensheathing cerebellar glomeruli and inhibits neurite outgrowth from granule neurons. *J Neurosci* **17**, 7784-7795 (1997).

96. Yamada, H., Watanabe, K., Shimonaka, M. & Yamaguchi, Y. Molecular cloning of brevican, a novel brain proteoglycan of the aggrecan/versican family. *J Biol Chem* **269**, 10119-10126 (1994).

97. Yoneda, K. *et al.* Regulation of aquaporin-4 expression in astrocytes. *Brain research. Molecular brain research* **89**, 94-102 (2001).

98. Badaut, J., Verbavatz, J.M., Freund-Mercier, M.J. & Lasbennes, F. Presence of aquaporin-4 and muscarinic receptors in astrocytes and ependymal cells in rat brain: a clue to a common function? *Neuroscience letters* **292**, 75-78 (2000).

99. Illarionova, N.B., Brismar, H., Aperia, A. & Gunnarson, E. Role of Na,K-ATPase alpha1 and alpha2 isoforms in the support of astrocyte glutamate uptake. *PLoS One* **9**, e98469 (2014).

100. Xue, Z. *et al.* Increased Na, K-ATPase alpha2 isoform gene expression by ammonia in astrocytes and in brain in vivo. *Neurochem Int* **57**, 395-403 (2010).

101. Hartford, A.K., Messer, M.L., Moseley, A.E., Lingrel, J.B. & Delamere, N.A. Na,K-ATPase alpha 2 inhibition alters calcium responses in optic nerve astrocytes. *Glia* **45**, 229-237 (2004).

102. Yan, Q., Sage, E.H. & Hendrickson, A.E. SPARC is expressed by ganglion cells and astrocytes in bovine retina. *J Histochem Cytochem* **46**, 3-10 (1998).

103. Mendis, D.B., Ivy, G.O. & Brown, I.R. SC1, a brain extracellular matrix glycoprotein related to SPARC and follistatin, is expressed by rat cerebellar astrocytes following injury and during development. *Brain Res* **730**, 95-106 (1996).

104. McKinnon, P.J. & Margolskee, R.F. SC1: a marker for astrocytes in the adult rodent brain is upregulated during reactive astrocytosis. *Brain Res* **709**, 27-36 (1996).

105. Mendis, D.B., Malaval, L. & Brown, I.R. SPARC, an extracellular matrix glycoprotein containing the follistatin module, is expressed by astrocytes in synaptic enriched regions of the adult brain. *Brain Res* **676**, 69-79 (1995).

106. Webersinke, G., Bauer, H., Amberger, A., Zach, O. & Bauer, H.C. Comparison of gene expression of extracellular matrix molecules in brain microvascular endothelial cells and astrocytes. *Biochemical and biophysical research communications* **189**, 877-884 (1992).

107. Lien, W.H., Klezovitch, O., Fernandez, T.E., Delrow, J. & Vasioukhin, V. alphaE-catenin controls cerebral cortical size by regulating the hedgehog signaling pathway. *Science* **311**, 1609-1612 (2006).

108. Stocker, A.M. & Chenn, A. Differential expression of alpha-E-catenin and alpha-N-catenin in the developing cerebral cortex. *Brain Res* **1073-1074**, 151-158 (2006).

109. Haidari, M. *et al.* Myosin light chain phosphorylation facilitates monocyte transendothelial migration by dissociating endothelial adherens junctions. *Cardiovascular research* **92**, 456-465 (2011).

110. Fausser, J.L. *et al.* Localization of antigens associated with adherens junctions, desmosomes, and hemidesmosomes during murine molar morphogenesis. *Differentiation* **63**, 1-11 (1998).

111. Johnson, K.J. & Boekelheide, K. Dynamic testicular adhesion junctions are immunologically unique. I. Localization of p120 catenin in rat testis. *Biology of reproduction* **66**, 983-991 (2002).

112. Stocker, A.M. & Chenn, A. The role of adherens junctions in the developing neocortex. *Cell adhesion & migration* **9**, 167-174 (2015).

113. Griffiths, G.S., Grundl, M., Allen, J.S., 3rd & Matter, M.L. R-Ras interacts with filamin a to maintain endothelial barrier function. *J Cell Physiol* **226**, 2287-2296 (2011).

114. Feng, Y. *et al.* Filamin A (FLNA) is required for cell-cell contact in vascular development and cardiac morphogenesis. *Proc Natl Acad Sci U S A* **103**, 19836-19841 (2006).

115. Sako-Kubota, K., Tanaka, N., Nagae, S., Meng, W. & Takeichi, M. Minus end-directed motor KIFC3 suppresses E-cadherin degradation by recruiting USP47 to adherens junctions. *Mol Biol Cell* **25**, 3851-3860 (2014).

116. Akong, K., McCartney, B.M. & Peifer, M. Drosophila APC2 and APC1 have overlapping roles in the larval brain despite their distinct intracellular localizations. *Dev Biol* **250**, 71-90 (2002).

117. Priya, R. *et al.* Coronin 1B supports RhoA signaling at cell-cell junctions through Myosin II. *Cell Cycle* **15**, 3033-3041 (2016).

118. Yamamoto, H. *et al.* Impairment of radial glial scaffold-dependent neuronal migration and formation of double cortex by genetic ablation of afadin. *Brain Res* **1620**, 139-152 (2015).

119. Mason, F.M., Tworoger, M. & Martin, A.C. Apical domain polarization localizes actin-myosin activity to drive ratchet-like apical constriction. *Nat Cell Biol* **15**, 926-936 (2013).

120. Liu, K.C. & Cheney, R.E. Myosins in cell junctions. *Bioarchitecture* **2**, 158-170 (2012).

121. Stoffler, H.E. *et al.* Targeting of the myosin-I myr 3 to intercellular adherens type junctions induced by dominant active Cdc42 in HeLa cells. *J Cell Sci* **111 ( Pt 18)**, 2779-2788 (1998).

122. Naydenov, N.G. & Ivanov, A.I. Spectrin-adducin membrane skeleton: A missing link between epithelial junctions and the actin cytoskeletion? *Bioarchitecture* **1**, 186-191 (2011).

123. Lee, H.G., Zarnescu, D.C., MacIver, B. & Thomas, G.H. The cell adhesion molecule Roughest depends on beta(Heavy)-spectrin during eye morphogenesis in Drosophila. *J Cell Sci* **123**, 277-285 (2010).

124. Roper, K. & Brown, N.H. Maintaining epithelial integrity: a function for gigantic spectraplakin isoforms in adherens junctions. *J Cell Biol* **162**, 1305-1315 (2003).

125. Chen, A. *et al.* E-cadherin loss alters cytoskeletal organization and adhesion in non-malignant breast cells but is insufficient to induce an epithelial-mesenchymal transition. *BMC cancer* **14**, 552 (2014).

126. Nobes, C.D. *et al.* A new member of the Rho family, Rnd1, promotes disassembly of actin filament structures and loss of cell adhesion. *J Cell Biol* **141**, 187-197 (1998).
