# Supplementary material for: Epigenetic Regulation by BAF Complexes Limits Neural Stem Cell Proliferation by Suppressing Wnt Signaling in Late Embryonic Development
Source: Stem Cell Reports. 2018 May 17;10(6):1734–50. doi: 10.1016/j.stemcr.2018.04.014 (PMC5993560; doi:10.1016/j.stemcr.2018.04.014)
Supplement: Document S2. Article plus Supplemental Information [file mmc9.pdf]

Stem Cell Reports
Article
OPEN ACCESS

Epigenetic Regulation by BAF Complexes Limits Neural Stem Cell Proliferation by Suppressing Wnt Signaling in Late Embryonic Development

Huong Nguyen,[1,8] Cemil Kerimoglu,[2,6,8] Mehdi Pirouz,[3,7] Linh Pham,[1] Kamila A. Kiszka,[1,4] Godwin Sokpor,[1] M. Sadman Sakib,[2,6] Joachim Rosenbusch,[1] Ulrike Teichmann,[3] Rho H. Seong,[5] Anastassia Stoykova,[3,4] Andre Fischer,[2,6] Jochen F. Staiger,[1,4] and Tran Tuoc[1,4,*]

[1]Institute of Neuroanatomy, University Medical Center, Georg-August- University, 37075 Goettingen, Germany
[2]Department of Psychiatry and Psychotherapy, University Medical Center, Georg- August-University Goettingen, 37077 Goettingen, Germany
[3]Max-Planck-Institute for Biophysical Chemistry, 37077 Goettingen, Germany
[4]DFG Center for Nanoscale Microscopy & Molecular Physiology of the Brain (CNMPB), 37075 Goettingen, Germany
[5]Department of Biological Sciences, Institute of Molecular Biology and Genetics, Research Center for Functional Cellulomics, Seoul National University, Seoul 151- 742, Korea
[6]Department for Systems Medicine and Epigenetics, German Center for Neurodegenerative Diseases, 37075 Goettingen, Germany
[7]Present address: Stem Cell Program, Division of Hematology/Oncology, Boston Children's Hospital, Department of Biological Chemistry and Molecular Pharmacology, Harvard Medical School, Boston MA 02115, USA
[8]Co-first author
*Correspondence: tran.tuoc@med.uni-goettingen.de
https://doi.org/10.1016/j.stemcr.2018.04.014

SUMMARY

During early cortical development, neural stem cells (NSCs) divide symmetrically to expand the progenitor pool, whereas, in later stages, NSCs divide asymmetrically to self-renew and produce other cell types. The timely switch from such proliferative to differentiative division critically determines progenitor and neuron numbers. However, the mechanisms that limit proliferative division in late cortical development are not fully understood. Here, we show that the BAF (mSWI/SNF) complexes restrict proliferative competence and promote neuronal differentiation in late corticogenesis. Inactivation of BAF complexes leads to H3K27me3-linked silencing of neuronal differentiation-related genes, with concurrent H3K4me2-mediated activation of proliferation-associated genes via derepression of Wnt signaling. Notably, the deletion of BAF complexes increased proliferation of neuroepithelial cell-like NSCs, impaired neuronal differentiation, and exerted a Wnt-dependent effect on neocortical and hippocampal development. Thus, these results demonstrate that BAF complexes act as both activators and repressors to control global epigenetic and gene expression programs in late corticogenesis.

INTRODUCTION

During vertebrate cerebral cortex development, neural stem cells (NSCs) undergo two types of temporally regulated cell division modes to generate distinct neural cell types. During early corticogenesis in mice (embryonic day 8.5–12.5 [E8.5–E12.5]), NSCs, also called neuroepithelial cells (NEs), mainly divide symmetrically to proliferate and expand their population (Dehay and Kennedy, 2007; Gotz and Huttner, 2005; Kriegstein and Alvarez-Buylla, 2009; Martynoga et al., 2012; Tuoc et al., 2014). At the onset of neurogenesis (E10.5), NEs differentiate into mature NSCs, also termed radial glial progenitors (RGs), which start to express astroglial markers (Hartfuss et al., 2001). This process coincides with the loss and appearance of tight and adherens junctional complexes respectively in the ventricular zone (VZ) (Aaku-Saraste et al., 1996; Sahara and O'Leary, 2009). Later, RGs primarily divide asymmetrically to produce an RG to maintain the proliferative pool, and either an excitatory neuron or a basal progenitor. Delayed RG differentiation from NEs causes aberrant neurogenesis (Sahara and O'Leary, 2009), yet factors that are required to suppress NE fate in late corticogenesis to ensure a balance between NSC proliferation and neuronal differentiation are unknown.

The temporal relationship and intricate balance between proliferative symmetric and neurogenic asymmetric divisions in the VZ of the cortex is controlled by diverse signaling pathways. Among these, Wnt/β-catenin signaling has been extensively investigated for its role in proliferative symmetric division (Chenn and Walsh, 2002). For example, elevation of Wnt signaling through overexpression of β-catenin massively enhanced cortical NSC proliferation (Chenn and Walsh, 2002). Interestingly, a recent study revealed irreversibility of the progression from proliferative to neurogenic division modes, thus implicating a default program in NSCs for division-mode transition during corticogenesis (Gao et al., 2014). As regulators of the spatiotemporal expression of developmental genes, epigenetic and chromatin regulatory mechanisms have been proposed to contribute to establishing the proliferative and differentiation competence of NSCs (Hirabayashi and Gotoh, 2010; Yao et al., 2016).

To investigate the possible involvement of chromatin-remodeling BAF (mSWI/SNF) complexes in this process, we applied a conditional deletion approach through

This is an open access article under the CC BY license (http://creativecommons.org/licenses/by/4.0/).

double-knockout (dcKO) of the BAF155 and BAF170 subunits, which eliminate the entire BAF complex during late cortical neurogenesis in transgenic mice. In the absence of BAF complexes, transcriptional profiling and epigenetic analyses revealed an enrichment of downregulated RG (astroglial, adherens junctions)- and neuronal differentiation-related genes, with both gene groups showing increased H3K27me3 repressive marks. In contrast, upregulated genes with increased H3K4me2 active marks were predominantly involved in the regulation of NE cell fate (e.g., tight junction feature), proliferation, cell cycle, and Wnt signaling-related pathways. The results of this study suggest that BAF complexes exert genome-wide control on both active H3K4me2 and repressive H3K27me3 marks during late cortical development by directly interacting with the corresponding H3 demethylases and regulating their activity. Phenotypically, we found that deletion of BAF complexes during late cortical neurogenesis leads to dysgenesis of the upper cortical layers and the hippocampal formation. These perturbations were rescued by inhibition of Wnt/β-catenin signaling. Together, these observations provide insights into distinct epigenetic regulatory mechanisms mediated by chromatin-remodeling BAF complexes as a key factor that suppresses the proliferative competence of NSCs during late cortical development.

RESULTS

Loss of BAF Complexes Causes a Genome-wide Increase in the Level of Both Active and Repressive Epigenetic Marks at Distinct Loci in the Developing Pallium during Late Neurogenesis

We previously reported that BAF complexes potentiate the activity of two main H3K27 demethylases, JMJD3 and UTX. Accordingly, elimination of BAF complexes during early corticogenesis leads to a global increase in repressive marks (H3K27Me2/3) and downregulation of gene expression at E13.5 (Narayanan et al., 2015; Nguyen et al., 2016). In further analysis, we performed co-immunoprecipitation (coIP) experiments on tissue lysates from the pallium of E17.5 wild-type (WT) embryos followed by mass spectrometry to identify BAF155/BAF170-interacting proteins. At E17.5, we found that BAF155 and BAF170 bind to the H3K27me2/3 demethylases, UTX/KDM6A and JMJD3/KDM6B, as shown in our previous study at E13.5 (Narayanan et al., 2015). BAF155/BAF170 was also observed to interact with H3K4me1/2 demethylase LSD1/KDM1A in the E17.5 pallium (Figures 1A, 1B, and S1A).

To investigate if BAF complexes regulate epigenetic programs in late cortical development, we crossed *Baf155*-floxed (*Baf155*$^{fl/fl}$) mice and *Baf170*-floxed (*Baf170*$^{fl/fl}$) mice with the *hGFAP*-Cre line to generate *dcKO* mutants. In contrast to the *Emx1*-Cre line used in our previous studies (Narayanan et al., 2015; Tuoc et al., 2009, 2013) with Cre recombination in the developing cortex as early as E10.5, the *hGFAP* promoter is not active in the pallium prior to E12.5. At E13.5, *hGFAP*-Cre activity is restricted to the medial pallium (MP), containing the hippocampal anlage and medial cortex (Figure S1B). From E15.5 onward, *hGFAP*-Cre activity extends to the dorsal pallium (DP; dorsal cortex) and lateral pallium (LP; lateral cortex) during development (Figure S1C). BAF155 and BAF170 proteins were not detected in the MP of dcKO mutants from E14.5 or in the entire VZ of the pallium from E15.5 onward (Figure S1D) (Narayanan et al., 2015), hence validating our *Baf155/Baf170* knockout system in late pallial progenitors.

Given the identified interaction of BAF complexes with the H3K27me2/3 demethylases KDM6A/B and H3K4me1/2 demethylase KDM1A in the E17.5 pallium, we next compared H3K27me3 repressive and H3K4me2 activatory marks in the E17.5 *dcKO* and control pallia. As reported previously, loss of BAF complexes in the E13.5 murine pallium in dcKO_*Emx1*-Cre mutants results in an increase in H3K27me3 levels (Narayanan et al., 2015). Similarly, chromatin immunoprecipitation sequencing (ChIP-seq) analysis performed using chromatin isolated from the E17.5 *dcKO* pallium also revealed an increase in H3K27me3 upon *Baf155/170* knockout (Figure 1C). Specifically, 181 genes showed a significant increase in these marks around their transcription start site (TSS) regions (±2000 bp) compared with 13 genes that showed a decrease (Table S1), a difference that likely reflects secondary effects and/or compensatory mechanisms. H3K27me3 is a broad chromatin mark localized not only at TSS but also spread over gene bodies. We also looked at the number of genes with altered H3K27me3 at their coding regions (including TSS). There were 484 genes with increased and 156 genes with decreased H3K27me3 (Figure 1E and Table S1). Strikingly, loss of BAF complexes in late corticogenesis resulted in a concurrent increase in activatory H3K4me2 marks in the E17.5 pallium (Figure 1D), with 1,265 genes showing a significant increase in this mark around their TSSs (Figure 1E, Table S1). Only 112 genes showed decreased H3K4me2, which again may represent some secondary effects. Importantly, genes affected by increased H3K27me3 and H3K4me2 were largely distinct (Figure 1E).

We also performed gene expression profiling of the *dcKO* pallium at E17.5 (Figure 1F). In contrast to the globally reduced gene expression in the *dcKO_Emx1*-Cre pallium at E12.5 (Narayanan et al., 2015), at E17.5, we found nearly equal number of downregulated and upregulated genes in the *dcKO* pallium (Figure 1F; Table S2).

Collectively, these data indicate that loss of BAF complexes during late corticogenesis induces an increase in activatory H3K4me2 and repressive H3K27me3 marks at

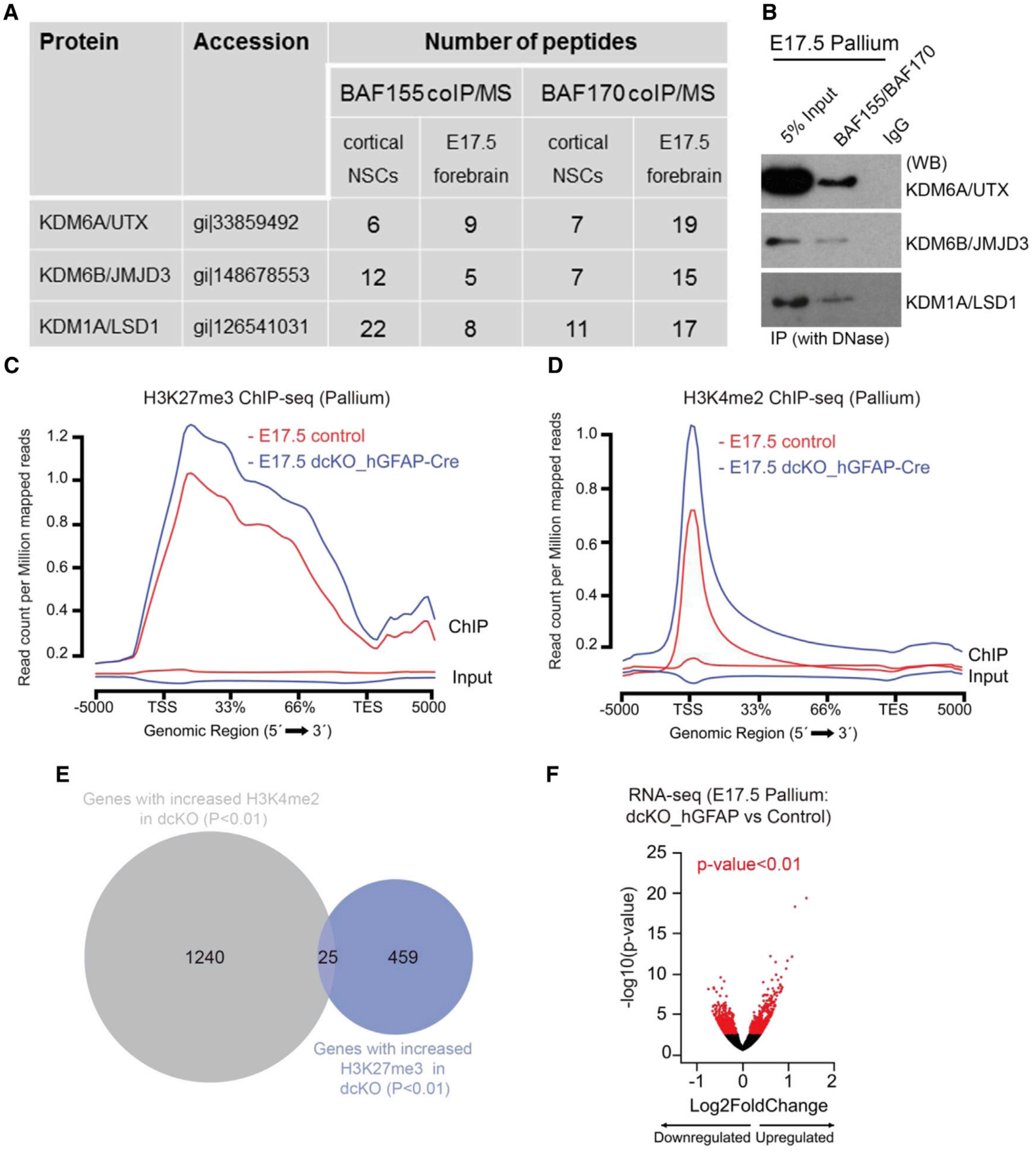

Figure 1. BAF Complexes Globally Control Epigenetic and Gene Expression Programs in Late Development Pallium

(A) Table showing the peptide number for KDM6A, KDM6B, and KDM1A proteins purified from BAF155 and BAF170 immunoprecipitates of protein extracts from NS5 cells, E13.5 or E17.5 forebrain.

(B) Interactions of BAF155 and BAF170 with KDM6A, KDM6B, and KDM1A were confirmed by coIP/western blot (WB) analyses of E17.5 pallium tissue.

(C–E) Distribution of H3K27me3 (C) and H3K4me2 (D) marks along gene bodies in the dcKO and control pallium at E17.5. H3K27me3 levels are increased in dcKOs. dcKO (E) genes with increased H3K4me2 or H3K27me3 marks in the dcKO pallium at E17.5 are largely non-overlapping.

(F) Volcano plot representing differentially regulated genes in the dcKO pallium at E17.5. Experimental replicates (n) = 4 (C, D; ChIP-seq), 4 (control for RNA-seq; F), 3 (dcKO_hGFAP-Cre for RNA-seq; F).

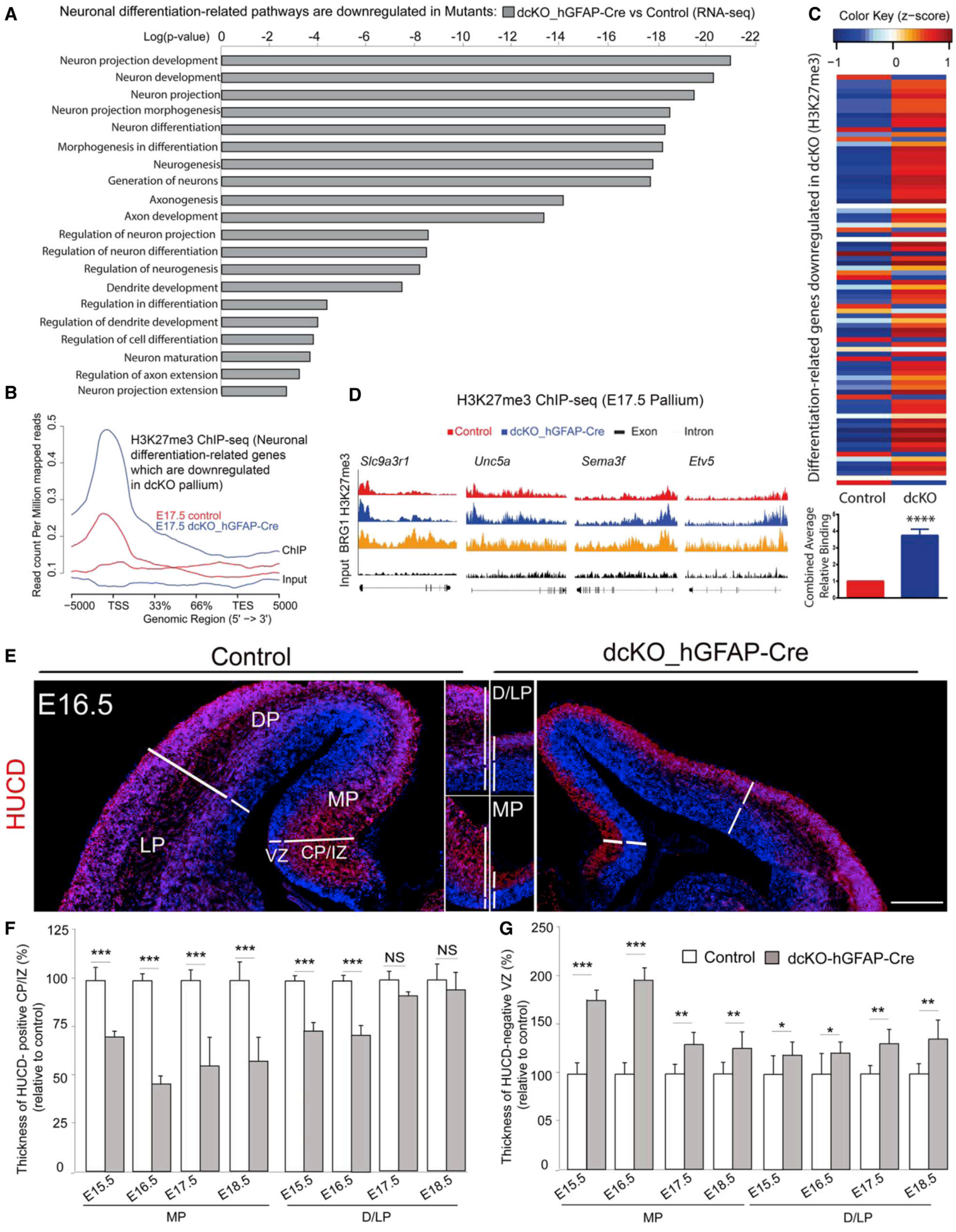

(legend on next page)

distinct sets of genes, thereby pointing to possible dual functions of BAF complexes as both activators and repressors in late cortical neurogenesis.

Conditional Inactivation of BAF Complexes during Late Cortical Development Impairs Neurogenesis of Upper Cortical Layer Neurons and the Hippocampus

We selected the downregulated genes in the E17.5 dcKO pallium in RNA sequencing (RNA-seq) and subjected them to functional category analysis. They are enriched in neuronal differentiation-related categories (Figure 2A, Table S2) and showed an overall increase in H3K27me3 mark (Figure 2B). Most of the differentiation-related genes that were significantly downregulated in *dcKO* mice (Table S3) showed an increase in H3K27me3. For some selected candidates, we also confirmed their downregulation and increased H3K27me3 by qPCR and ChIP-qPCR respectively (Figures S2A and S2B).

Next, we asked if these genes with decreased expression and increased H3K27me3 are directly bound by the BAF complexes. We made use of a previously published ChIP-seq dataset (GEO: GSE37151) for BRG1 in the developing mouse forebrain (Attanasio et al., 2014). Strikingly, the majority of genes that showed increased H3K27me3 in dcKO cortices were also bound by BRG1 (Figure S2C), with sites of increased H3K27me3 co-localizing with BRG1 binding sites (Figure 2D).

We further confirmed these observations in a reverse approach, in which we first selected the genes with increased H3K27me3 in E17.5 dcKO (Figure S2D) and subjected them to functional category analysis. Again, they also mostly fell under neuronal differentiation-related categories (Figure S2E). We then examined their expression in our RNA-seq analysis. As expected, most of them were downregulated in dcKO embryos.

Because the *hGFAP* promoter is active early in the MP (from E13.5) and later in the DP and LP (from E14.5) (Figures S1B and S1C), we compared neuronal differentiation between controls and dcKO mutants, in both the MP and at the area between the DP and LP (D/LP). Neurogenesis in late (E15.5–E17.5) development of the pallium in dcKO mutants was decreased, as shown by a decrease in the thickness of the cortical plate (CP) and intermediate zone (IZ), marked by the expression of the pan-neuronal markers HUCD, TUBB3, and NEUN in both the cortex (D/LP) and hippocampus (MP) (Figures 2E and 2F). Consistent with this, immunofluorescence (IF) analyses of neuronal subtype markers indicated that loss of BAF155 and BAF170 led to a significant decrease in the number of late-born SATB2$^+$ or BRN2$^+$ neurons, but not early-born TBR1$^+$ neurons, in the DP and LP (Figures S3A–S3D).

To study neurogenesis specifically in the MP, we performed IF on sections from E15.5–E17.5 control and dcKO embryonic brains using the antibody ZBTB20 (Figure S3E), which outlines the hippocampal anlage as early as E14.5 and is confined postnatally to hippocampal cornu ammonis (CA1–CA3) regions. ZBTB20 staining revealed remnants of the hippocampus proper (Figure 3C) in mutants compared with controls at all examined stages, E14.5–E17.5 (Figures S3E and S3G). Indeed, three-dimensional (3D) reconstruction of ZBTB20 expression also revealed a substantial reduction in the volume of the developing hippocampus in dcKO embryos at E15.5 (Figure S3H and Video S1). Consistently, immunostaining of the dentate gyrus (DG) with its specific marker PROX1 indicated agenesis of this hippocampal domain (Figures S3F and S3I). In the DP/LP of mutants, whereas the generation of lower layer (TBR1$^+$/L6, and CTIP2$^+$/L5) neurons was only mildly decreased, the number of late-born SATB2$^+$, and BRN2$^+$ L4–L2 neurons was strongly diminished (Figures 3A and 3B). In further support, we found that BAF complexes control expression of sets of gene exerting important roles in generation of cortical layers and hippocampal development (Table S4).

To gain additional evidence about how the defect in neuronal differentiation is caused by increased level of H3K27me3, we used GSK-J4, a potent selective H3K27 demethylase (JMJD3 and UTX) inhibitor (Kruidenier et al., 2012). The elevated level of H3K27me3 by GSK-J4 administration significantly decreased the number of late-born

Figure 2. H3K27me3-Linked Silencing of Neuronal Differentiation-Related Genes in BAF Complex-Deleted Pallium in Late Stages
(A) Neuronal differentiation-related genes are downregulated in the dcKO pallium at E17.5.
(B) General H3K27me3 profile plot of neuronal differentiation-related genes that are downregulated in dcKO pallium.
(C) Upper panel: heatmap depicting the changes in H3K27me3 levels at neural differentiation-related genes that are downregulated in dcKO pallium at E17.5 individually. Lower panel: average relative H3K27me3 binding levels on those genes combined.
(D) Integrated genome browser views of H3K27me3 and BRG1 (GEO: GSE37151; Attanasio et al., 2014) binding along representative neural differentiation-related genes downregulated in dcKO pallium.
(E–G) IF (E) and quantitative (F and G) analyses indicate that the loss of BAF155 and BAF170 leads to a diminished thickness of the HUCD$^+$ cortical plate (CP) and intermediate zone (IZ) (F), and expanded thickness of the HUCD$^-$ VZ (G) in the entire pallium at E15.5–E18.5.
Values are presented as means ± SEMs (*p < 0.05; **p < 0.01; ***p < 0.005; ****p < 0.0001). Experimental replicates (n) = 6 (F and G).
Abbreviations: VZ, ventricular zone; CP, cortical plate; IZ, intermediate zone; MP, medial pallium; DP, dorsal pallium; LP, lateral pallium.
Scale bar represents 100 μm (E).

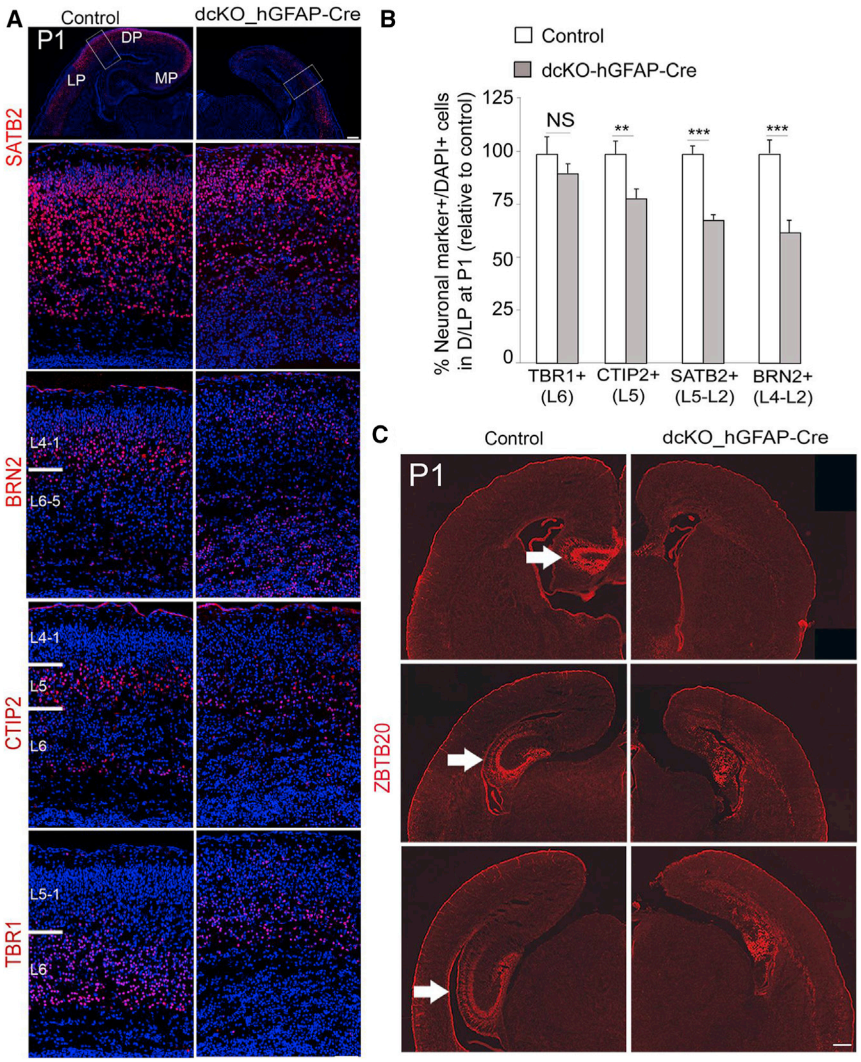

Figure 3. BAF Complexes Are Required for the Formation of Cortical Upper Layers and the Hippocampus

(A and B) IF (A) and statistical (B) analyses of cortical phenotypes at postnatal stage 1 (P1) in a comparable dorsal/lateral area, immunostained for the indicated neuronal layer markers. NS, not significant.

(C) IF analysis of Ztbt20 revealed that the hippocampus is underdeveloped in mutants (denoted by arrow).

Values are presented as means ± SEMs (**$p < 0.01$; ***$p < 0.005$). Experimental replicates (n) = 4 (B). Abbreviations: MP, medial pallium; DP, dorsal pallium; LP, lateral pallium; L, layer. Scale bars represent 100 μm (A) and 100 μm (C).

SATB2^{+} and CUX1^{+} neurons (Figures S4A–S4D), as observed in dcKO pallium with enhanced level of H3K27me3.

Together, these findings suggest that deletion of BAF complexes in late NSCs leads to H3K27me3-linked silencing of neuronal differentiation genes and results in diminished late cortical and hippocampal neurogenesis.

The NSC Pool Is Increased at Late Development Stages in the dcKO Pallium

Our previous data indicated that the loss of BAF complexes leads to large-scale downregulation of gene expression in early cortical development (Narayanan et al., 2015; Nguyen et al., 2016). Intriguingly, the late elimination of BAF complex function also led to upregulation of a substantial number of genes. In order to assess the role of the genes upregulated in dcKO embryos, we applied the aforementioned strategy. Functionally, they mainly converged into cell proliferation-related categories (Figure 4A, Table S2). Moreover, these genes also showed an overall increase in H3K4me2 in the dcKO pallium (Figure 4B, Table S3).

Next, we assessed the changes in H3K4me2 levels at their individual TSS regions. As expected, most of them had an increase in this activatory mark with the overall trend being highly significant (Figure 4C) and they mostly converged into cell cycle-related groups (Figures S2D and S2E). The sites of increased H3K4me2 also substantially overlapped with BAF complex (BRG1) binding (Figures 4D and S2C). Selected candidates were confirmed by qPCR and ChIP-qPCR (Figures S2A and S2B). Because the expression of genes encoding H3 demethylases LSD1/kdm1a, UTX/KDM6A, and JMJD3/KDM6B was unaltered in dcKO cortex in our RNA-seq experiment (Table S2), it is possible that BAF complexes control the methylation of H3K4 and H3K27 through mechanisms other than activating or inhibiting the expression of genes coding for

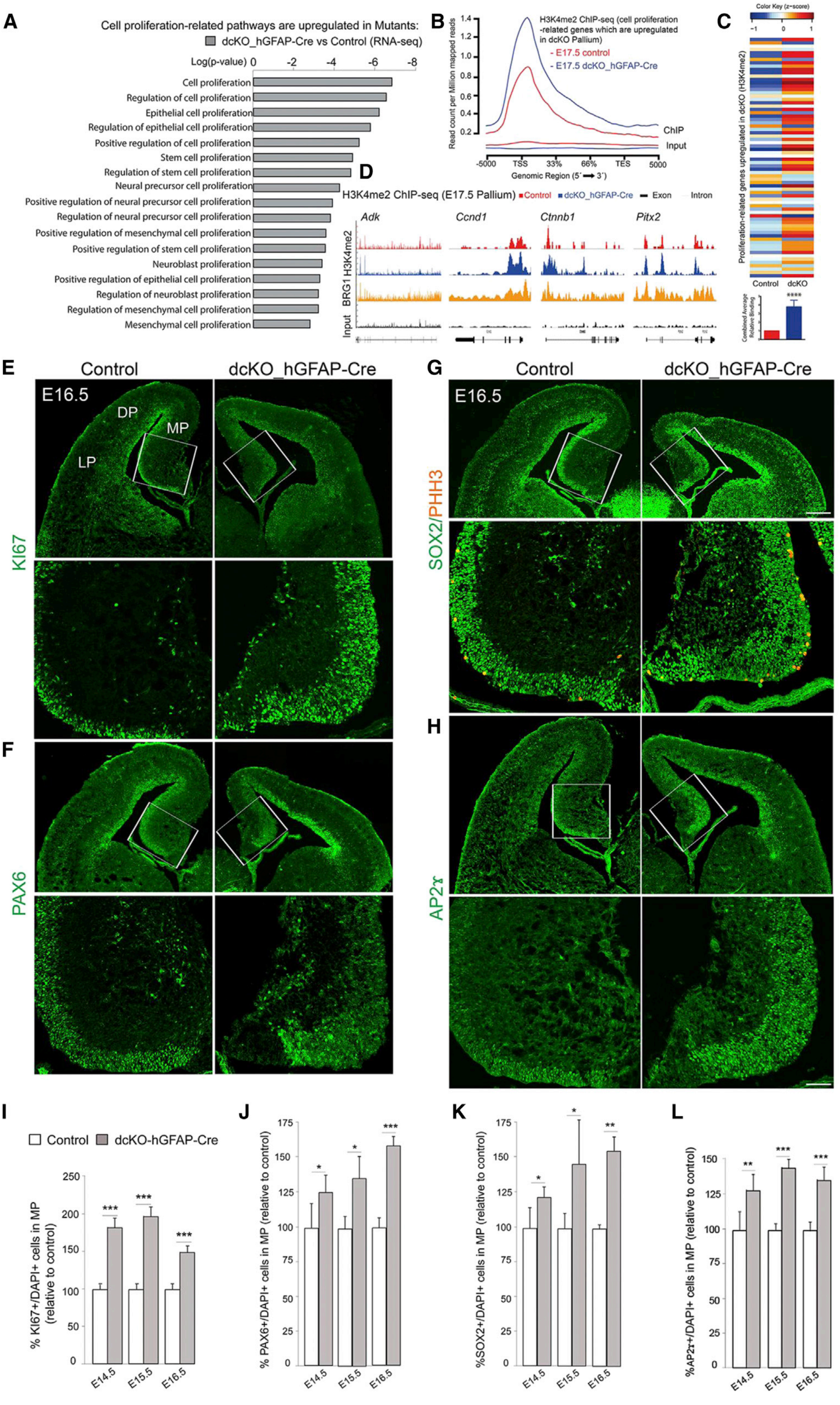

(legend on next page)

these H3 demethylases. Our earlier study indicated that BAF complexes potentiate the H3K27 demethylase activity of UTX/KDM6A and JMJD3/KDM6B (Narayanan et al., 2015), which encouraged us to investigate whether endogenous BAF155 and BAF170 are required for full H3K4 demethylase activity of LSD1/KDM1A. We therefore performed the histone demethylase KDM1/LSD1 activity quantification assay (see Experimental Procedures). The results revealed that significantly less H3K4 is demethylated in BAF155/BAF170-ablated NSCs compared with control counterparts (Figure S2G).

IF analysis of the expression of HUCD, TUBB3, and NEUN indicated an enlargement of the VZ in the dcKO pallium, more strongly in MP than in D/LP (Figures 2E and 2G). Reconstruction analyses showed that the volume of the hippocampal neuroepithelium, as revealed by PAX6 expression, is larger in the mutant MP (Figures S5A and S5B; Video S1). These data suggest increased pools of progenitors in proliferative zones of the dcKO pallium. Indeed, more KI67$^+$ mitotically active cells were found in mutants than in controls (Figures 4E, 4I, and S5C). We then examined pools of RGs and intermediate progenitors (IPs) (Figures 4F–4H, 4J–4L, and S5D–S5G). Similar to the increased number of KI67$^+$ mitotic cells, the number of PAX6$^+$, SOX2$^+$, and AP2γ^+ NSCs in the VZ gradually increased from E14.5 in the mutant MP (Figures 4F–4H). Notably, the effect was more profound in the NSC pool in the MP than in the D/LP (Figures S5C–S5F). This possibly relates to the spatiotemporal *hGFAP*-Cre activity, exerting early activity in the MP (Figures S1B and S1C). In contrast to the increased number of RGs, the number of TBR2$^+$ IPs was decreased (Figures S5D and S5G), indicating disruption of neuronal differentiation in the mutant pallium.

To substantiate the effect of the H3K4me2 level on the cortical NSC pool, we examined an increased H3K4me2 by using (±)-trans-2-phenylcyclopropylamine hydrochloride (2-PCPA), a specific inhibitor of LSD1 histone demethylase. 2-PCPA has been shown to increase H3K4me2 in mouse brain (Sun et al., 2010). The treatment of 2-PCPA also led to an increased pool of PAX6$^+$, SOX2$^+$ NSCs in developing cortex (Figures S4E–S4G).

Together, these findings suggest that, in the absence of BAF155/BAF170, at late corticogenesis (E14.5–E17.5), NSCs in the VZ are kept in the proliferative phase rather than differentiating into IPs and/or neurons.

RGs Acquire an NE-like Identity in the BAF155/BAF170-Deficient Pallium

The appearance of RGs in the pallium is marked by initiation of the expression of the astrocytic differentiation markers GLAST and BLBP at E12.5 (Hartfuss et al., 2001; Sahara and O'Leary, 2009). At E13.5 and E14.5, expression level of GLAST and BLBP is comparable between control and mutants (Figures S5H–S5L). Following IF analysis at later stages (E15.5–E16.5) we observed that, despite the increased number of PAX6$^+$/SOX2$^+$/AP2γ^+ NSCs (Figures 4F and 4G), immunopositive signals for GLAST and BLBP were diminished in the DP/LP and largely undetectable in the MP in dcKO mutants (Figures 5A and 5D).

Another hallmark for NE-RG cell transition is the replacement of tight junctional complexes (NE trait) with adherens junctions (RGs trait) (Aaku-Saraste et al., 1996; Sahara and O'Leary, 2009). Notably, we found that, during late corticogenesis, many genes encoding for tight junction proteins (e.g., *Amot*, *Mpp5*, *Occludin*, *Inadl*, *Pkd2*, *Dlg1*, *Cftr*, *Tgfb3*) were significantly upregulated and also those involved in adherens junction proteins (e.g., *Tns3*, *Plec*, *Ptk2b*, *Zo1*, *α-Catenin*, *Kiaa1462/Jcad*, *filamin*, *Camsap3*, *Apc*, *Myh9*, *Myo1e*, *Sptan1*, *Itga1*, *Rnd1*) were downregulated in the dcKO pallium (Tables S2 and S4). Additionally, we examined VZ expression of OCCLUDIN (a tight junction marker) and ZO1, α-CATENIN (adherens junction markers) localized at the apical surface. OCCLUDIN is normally downregulated in NEs as they differentiate into RGs (Aaku-Saraste et al., 1996; Sahara and O'Leary, 2009). At E13.5–E16.5, expression of OCCLUDIN at the apical

Figure 4. Loss of BAF155 and BAF170 Causes H3K4me2-Linked Upregulation of Genes Involved in the Mitotic Cell Cycle and Proliferation in Late Cortical Development

(A) Proliferation- and cell-cycle-related genes are upregulated in the dcKO pallium at E17.5.

(B) General H3K4me2 profile plot at proliferation-related genes that are upregulated in dcKO pallium.

(C) Upper panel: heatmap depicting the changes in H3K4me2 levels at proliferation-related genes that are upregulated in dcKO pallium at E17.5. Lower panel: average relative H3K4me2 binding levels on those genes combined.

(D) Integrated genome browser views of H3K4me2 and Brg1 binding (GEO: GSE37151) (Attanasio et al., 2014) along representative proliferation-related genes upregulated in dcKO pallium.

(E–H) Representative images showing IF analyses of coronal sections of control and dcKO pallium at E16.5 using antibodies that specifically label the indicated NSC markers. Lower panels: higher-magnification images of areas indicated by white boxes. Note that a similar image of triple channels for PAX6/TBR2/CASP3 is shown in Figure S6H.

(I–L) Quantitative analyses indicated increased numbers of NSCs in the MP of dcKO mutants at the indicated stages.

Values are presented as means ± SEMs (*$p < 0.05$; **$p < 0.01$; ***$p < 0.005$; ****$p < 0.0001$). Experimental replicates (n) = 6 (I and L), 4 (J and K). Abbreviations: TSS, transcription start site; TES, transcription end site; MP, medial pallium; DP, dorsal pallium; LP, lateral pallium. Scale bars represent 100 μm (G) and 50 μm (H).

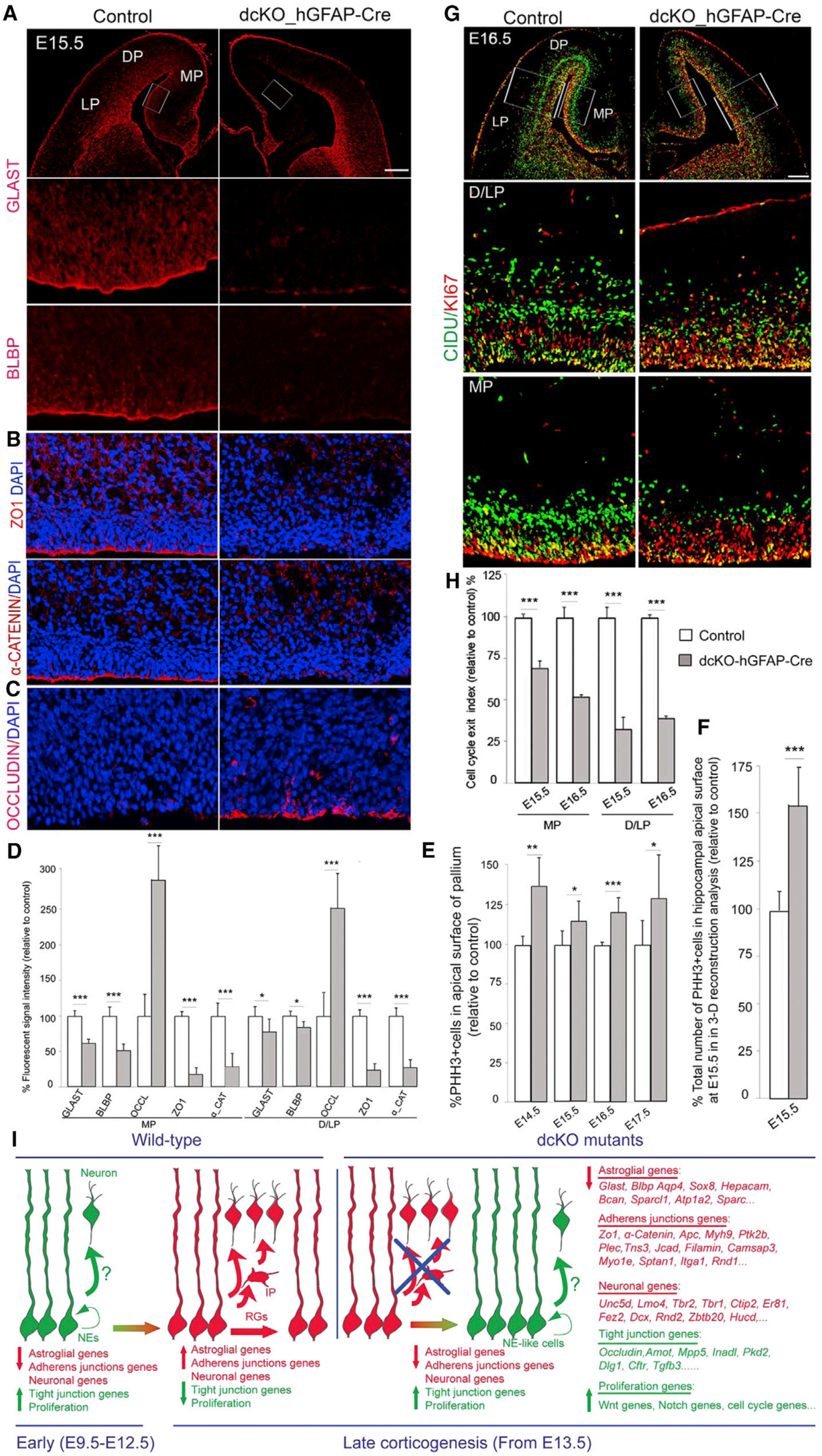

(legend on next page)

surface of the VZ in the control pallium was undetectable, whereas its expression was strongly upregulated in the dcKO pallium at E15.5–E16.5 (Figures 5C, 5D, and S5I). The expression of adherens junction markers ZO1, and α-CATENIN at the apical surface of RGs was not affected at E13.5–E14.5 (Figures S5J–S5L) but was largely absent at E16.5–E17.5 in dcKO cortex (Figures 5B and 5D).

BAF complexes seem not only to block NE fate in late pallium development but also control the differentiation from NEs to RGs, as shown by downregulated expression of the RG markers BLBP and GLAST together with upregulated expression of the NE marker OCCLUDIN in the dcKO_*Emx1*-Cre cortex at E13.5 (Figures S5M–S5O). Thus, our data revealed that the downregulation of the expression of astroglial and adherens junction markers is correlated with upregulation of tight junction markers in late cortical development of dcKO mutants. These complementary datasets indicate that deletion of BAF complexes during late development of the pallium dedifferentiates RGs to NE-like cells.

Change in Spindle Orientation and Increased Proliferative Capacity of NSCs in the BAF155/BAF170-Deficient Pallium

To assess the implications of the retention of an NE-like identity in the mutant cortex, we first found out whether the dedifferentiation from RGs to NE-like cells was consequent to or caused altered spindles orientation. We stained E15.5–E16.5 sections from control and dcKO pallium using antibodies against PVIM and PHH3 to mark mitotic cells and chromatin, respectively (Figure S5A). The division angles of apical RGs were quantified and categorized based on cleavage angle: vertical (60°–90°), oblique (30°–60°), and horizontal (0°–30°). Notably, more progenitors with vertical cleavage were detected in the mutant pallium than in controls (Figures S5A and S5B), suggesting that the loss of BAF complexes in late pallial development induces proliferative symmetric divisions, which mainly generate NEs and RGs.

The increased number of $PAX6^+/SOX2^+$ NSCs suggested that RGs were kept in the cell cycle to promote their proliferation, instead of exiting to become neurons. To ascertain whether loss of functional BAF155 and BAF170 leads to altered cell proliferation, we labeled M-phase cells by immunostaining with an anti-PHH3 antibody. Quantitative comparisons of immunostained medial brain sections of the E14.5–E17.5 pallium (Figures 4A and 5D) and 3D reconstruction analyses of the entire hippocampus at E15.5 (Figure 5E) indicated that the loss of BAF155 and BAF170 resulted in an increased number of $PHH3^+$ cells in the pallium (Figures 5D and 5E).

To better characterize BAF155/BAF170 loss-of-function effects on neuronal differentiation, we next used a thymidine analog (CIdU) injection paradigm (24-hr CIDU pulse labeling) to establish a quantitative *in vivo* cell cycle exit index in the developing MP and D/LP (Figures 5G and 5H). We also performed double-labeling IF using antibodies against CIDU to label both cycling progenitors and those that recently exited the cell cycle, and KI67 for proliferating progenitors in all cell cycle phases. Statistical analyses revealed a significantly lower cell cycle exit index in dcKO mutants compared with controls (Figures 5G and 5H). To examine whether BAF155/BAF170-deficient NSCs undergo several proliferative rounds, we again detected the sequential incorporation of different thymidine analogues (CIdU, IdU) into cortical NSCs (Figure S6C). Given that cell cycle length of cortical progenitors between E14.5 and E16.5 is about 15–18 hr per cycle (Takahashi et al., 1995), pregnant mice were injected with CIdU (at E14.5) and IdU (at E15.5). Tissue was collected at E16.5 and processed for IHC analysis with antibodies against CIDU, IDU, and KI67 (Figure S6D). The cortical cells between 1 and 3 successive rounds of cell

Figure 5. NE-like Cells in the BAF-Complex-Deleted Pallium in Late Development Retain Their Highly Proliferative Competence

(A–C) Immunostaining of the control and dcKO pallium sections at E15.5 for indicated markers revealed an altered cell identity from $GLAST^{high+}/BLBP^{high+}/ZO^{high+}/\alpha\text{-Catenin}^{high+}/OCCLUDIN^{-}$ RGs in controls to $GLAST^{low+}/BLBP^{low+}/ZO^{low+}/\alpha\text{-Catenin}^{low+}/OCCLUDIN^{high+}$ NEs in dcKO MP.

(D) Quantification and statistical analysis of (A)–(C) are shown.

(E and F) Quantitative analyses showing that the loss of BAF155 and BAF170 leads to an increase in mitotic $PHH3^+$ RGs in the pallium at E14.5–E16.5 (E). Note that quantification of $PHH3^+$ cells (F) was done in the entire developing hippocampus ($PAX6^+/ZBTB20^+$) at E15.5 using 3D reconstruction (see also Figures S5A and S5B, Video S1).

(G) Images showing double IF at E16.5 for CIDU, and KI67 in control and dcKO mutants.

(H) Quantitative analyses showing a significantly lower exit index (number of $CIDU^+/KI67^-$ cells per total number of $CIDU^+$ cells) in mutants in D/LP and MP areas than in controls.

(I) Schema illustrating that a higher proportion of RG progenitors in the late-stage (from E13.5) dcKO pallium acquire NE-like identity (i.e., downregulated expression of astroglial, adherens junction, differentiation genes and upregulated expression of tight junction, proliferation genes).

Values are presented as means ± SEMs (*$p < 0.05$; **$p < 0.01$; ***$p < 0.005$). Experimental replicates (n) = 6 (D and E), 4 (F and H). Abbreviations: NE, neuroepithelial cell; RG, ventricular radial glial progenitors, Hi, hippocampus; Cx, cortex; MP, medial pallium; DP, dorsal pallium; LP, lateral pallium. Scale bars represent 100 μm (A and G).

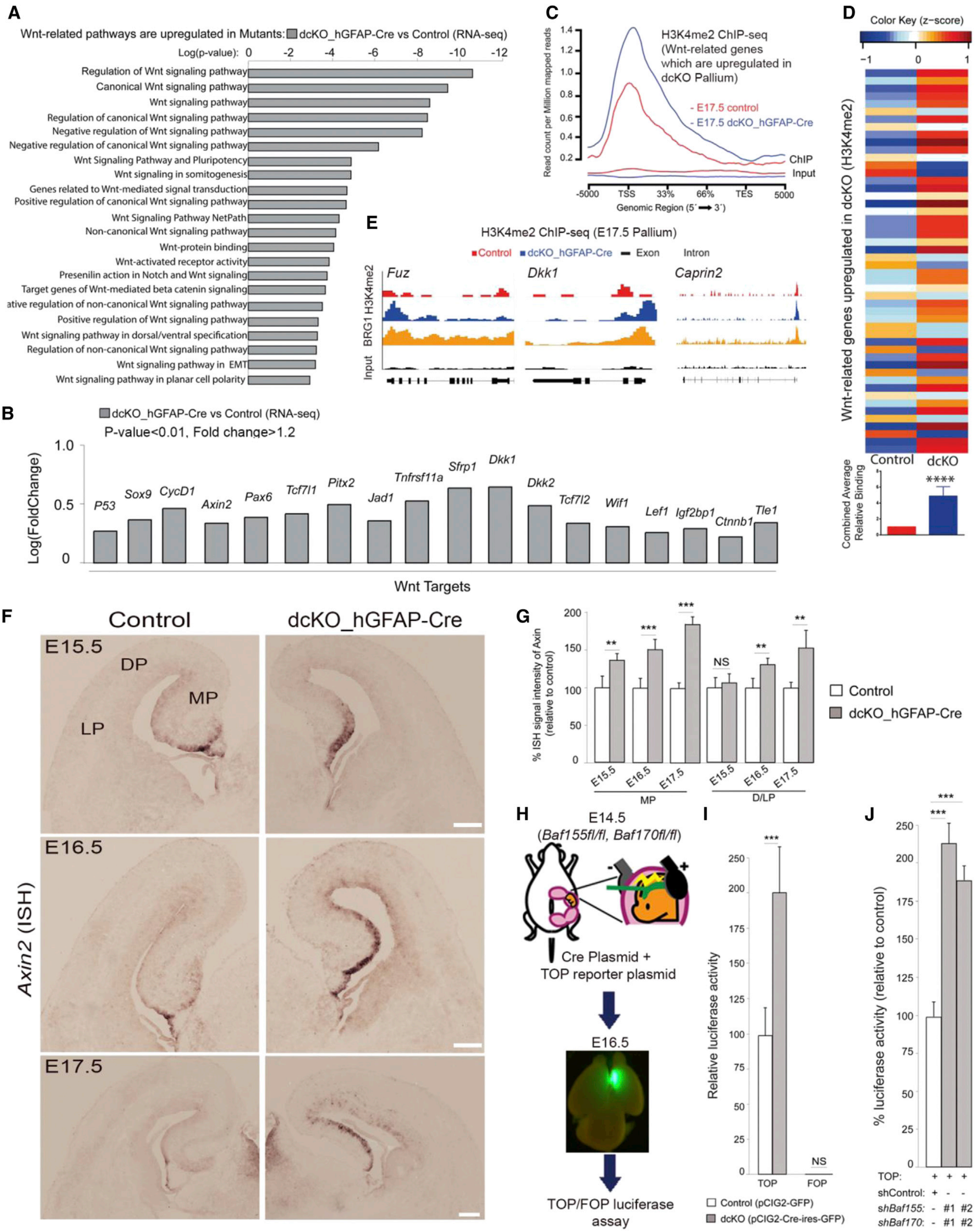

(legend on next page)

division were labeled as follows: (1) cells from the first and the second cell divisions as well as their progenies are marked with CIDU (in green) and with IDU (in violet) respectively; (2) cells in third mitotic cell cycle will be labeled by KI67 (red). Our statistical analysis (Figure S6E) indicated that, between E14.5 and E16.5, many cortical progenitors exit from the first ($CIDU^+/IDU^-/KI67^-$: 6% ± 0.83% in control and 0.7% ± 0.38% in dcKO, green part of chart) and second ($CIDU^+/IDU^+/KI67^-$: 73% ± 2.52% in control and 31.82% ± 3.30% in dcKO, violet part of chart) cell cycles in control cortex, whereas a large fraction of NSCs further enters third ($CIDU^+/IDU^+/KI67^+$: 20.16% ± 1.94% in control and 67.47% ± 9.70% in dcKO, red part of chart) cell cycle in dcKO cortex.

We also examined apoptosis at different stages from E14.5 to E18.5 in the MP by performing IF for CASP3 (Figures S6F–S6K). Compared with controls, significantly higher numbers of dying cells were found in the MP of dcKO pallium (Figures S6F–S6J). Particularly, most apoptotic cells in mutants were $PAX6^+$ RGs, while apoptotic $TBR2^+$ IPs were detected to a lesser extent. Apoptotic $HUCD^+$ neurons were rarely detected (Figures S6F–S6I and S6K), and this paralleled our previously observed apoptotic effect of selective loss of BAF complex in post-mitotic neurons. The latter effect is further supported by the observation that selective elimination of BAF155 and BAF170 in post-mitotic neurons in dcKO_*Nex*-Cre had no effect on the populations of $CTIP2^+/ZBTB20^+$ neurons, $PAX6^+/SOX2^+$ NSCs (Figures S6L–S6N) or $CASP3^+$ apoptotic cells (Narayanan et al., 2015).

Collectively, these findings indicate that the deletion of BAF complexes results in H3K4me2-linked activation of proliferation- and cell-cycle-associated genes. This resulted in three main morphogenetic defects of the dcKO pallium: (1) an expanded pool of NSCs, (2) diminished neurogenesis in late corticogenesis, and (3) malformed late-formed structures such as upper cortical layers and the hippocampus.

Elimination of BAF155 and BAF170 De-represses Wnt Signaling in Late Corticogenesis

Previous work has suggested that enhanced Wnt signaling promotes cortical NSC proliferation (Chenn and Walsh, 2002; Machon et al., 2007). We found that, during late corticogenesis, a considerable number of genes involved in Wnt signaling, including many Wnt target genes, were significantly upregulated in the dcKO pallium (Figures 6A, 6B, and S7A, Table S2). These genes showed an overall (Figures 6C and 6D) increase in H3K4me2 levels. Moreover, their TSS regions, where increased H3K4me2 is observed in dcKO embryos, also coincide with BRG1 binding sites (Figure 6E).

To provide additional support, we also performed *in situ* hybridization (ISH) analysis of the expression of *Axin2*, a direct target of Wnt/β-catenin activity. This analysis showed that, unlike controls, in which *Axin2* mRNA staining was faint and confined mostly to the MP at E15.5–E17.5, the BAF complex-deficient pallium exhibited diffused *Axin2* staining in the MP VZ at E15.5 and throughout the pallium VZ at E16.5–E17.5 (Figures 6F and 6G).

To further address the capacity of BAF complexes to regulate Wnt signaling, we performed *in vivo* reporter assays by electroporating a luciferase promoter construct TOP, containing β-catenin/TCF binding sites and a mutated form, FOP, as negative control into the embryonic brain. To eliminate BAF function, we electroporated TOP-/FOP-FLASH reporter plasmids plus a Cre-expressing plasmid into the E14.5 MP of $Baf155^{fl/fl}$:$Baf170^{fl/fl}$ embryos. We then examined isolated tissue samples from the MP using the TOP/FOP luciferase assay. These analyses indicated that BAF complex knockout in the pallium significantly enhanced TOP-, but not FOP-reporter activity (Figure 6H and 6I). Similarly, dual silencing of *Baf155* and *Baf170* markedly increased Wnt signaling activity in Neuro2A cells *in vitro* (Figure 6J), suggesting that BAF complex deficiency indeed

Figure 6. BAF Complexes Suppress Wnt Signaling Activity
(A) Wnt-related genes are upregulated in the dcKO pallium at E17.5.
(B) Wnt target genes upregulated in the dcKO pallium are shown.
(C) General H3K4me2 profile plot of Wnt-related genes that are upregulated in dcKO pallium.
(D) Upper panel: heatmap depicting the changes in H3K4me2 levels of Wnt-related genes that are upregulated in dcKO pallium at E17.5. Lower panel: average relative H3K4me2 binding levels on those genes combined.
(E) Integrated genome browser views of H3K4me2 and BRG1 binding (GEO: GSE37151) (Attanasio et al., 2014) along representative Wnt-related genes upregulated in dcKO pallium.
(F and G) ISH (F) and quantitative (G) analyses comparing the expression of the Wnt target *Axin2* in the control and dcKO pallium at E15.5–E17.5.
(H–J) *In vivo* (H and I) and *in vitro* (J) luciferase assay indicating higher Wnt signaling activity in BAF155/BAF170-depleted pallial cells (I) and in Neuro2A cells (J) compared with control cells.
Values are presented as means ± SEMs ($^{**}p < 0.01$; $^{***}p < 0.005$; $^{****}p < 0.0001$). Experimental replicates (n) = 6 (G and J), 4 (I). Abbreviations: TSS, transcription start site; TES, transcription end site; MP, medial pallium; DP, dorsal pallium; LP, lateral pallium. Scale bars represent 100 μm (F).

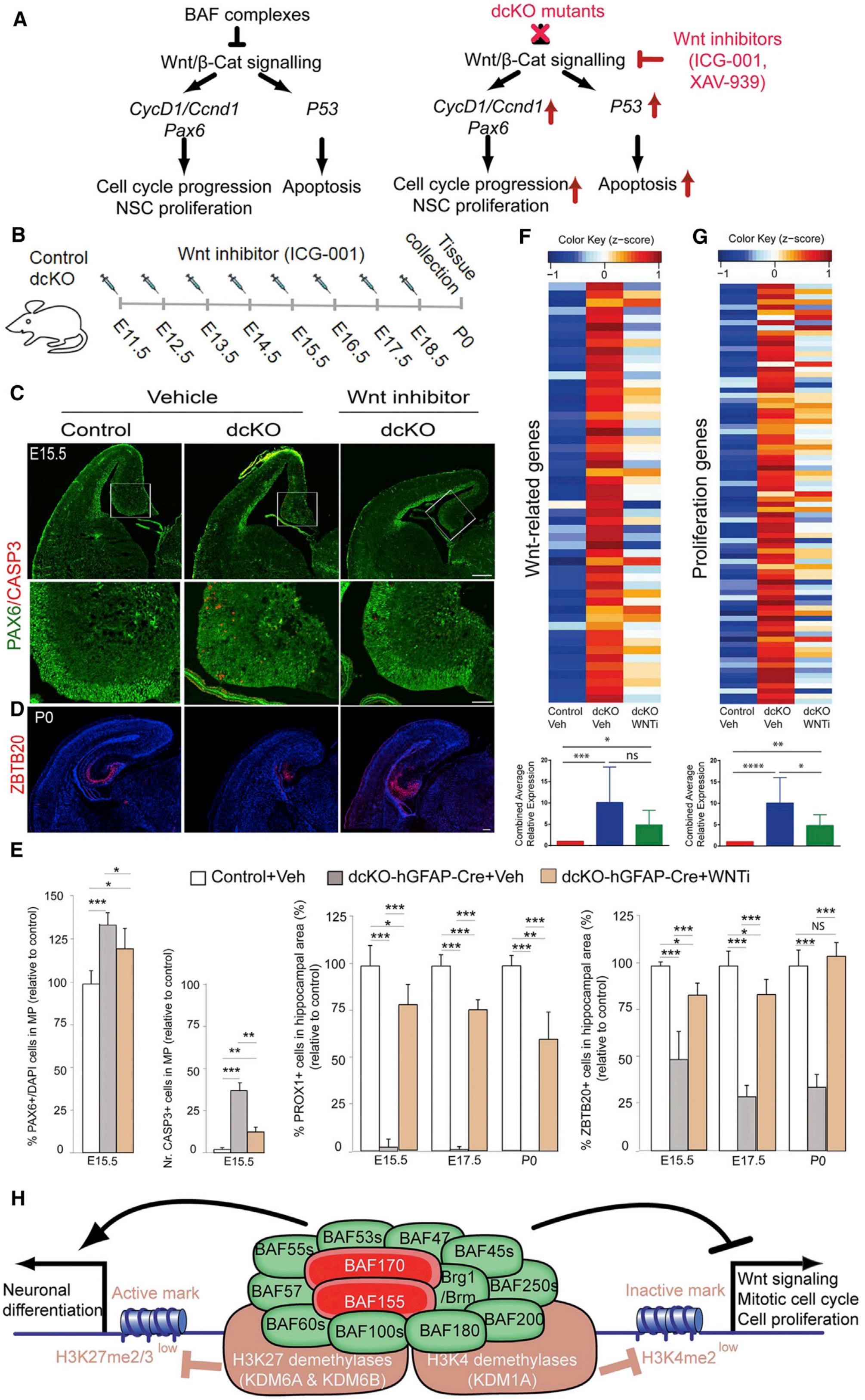

(legend on next page)

increased the transcriptional activity of the Wnt target genes that control NSC proliferation. Such candidate genes (e.g., *Pax6*, *Ap2γ*, and *Cyclin D1*) are critical for the timely progression of the cell cycle (Figure 7A).

Next, we directly determined whether BAF complex in the MP regulates hippocampal development via suppression of Wnt signaling. To this end, we used ICG-001, a Wnt signaling inhibitor, to perform rescue experiments (Figures 7A and 7B). Starting from E11.5, pregnant mice were intraperitoneally injected daily with an ICG-001 solution, and brain samples were collected at E15.5, E17.5, and postnatal stage 0 (P0) (Figure 7B). ICG-001 treatment of dcKO mutants resulted in the reversal of NE-like cell characteristics (BLBP^{low}/GLAST^{low}/OCCLUDIN^{high}) to RG features (BLBP^{high}/GLAST^{high}/OCCLUDIN^{low}) that typify the WT pallium (Figures S7B–S7E). Furthermore, IF analyses at E15.5 with PAX6 and CASP3 antibodies revealed that the Wnt inhibition decreased the number of PAX6^{+} NSCs and CASP3^{+} apoptotic cells (Figures 7C and 7E) in dcKO mutants. Concurrently, ICG-001 administration in dcKO mutants caused a near-WT increase in the number of PROX1^{+} DGs and ZBTB20^{+} hippocampal neurons in MP (Figures 7D and 7E) and also in the number of CTIP2^{+}, SATB2^{+}, and CUX1^{+} cortical neurons in L/DP (Figures S7F–S7H). Strikingly, treatment with the Wnt inhibitor almost completely rescued the aberrant hippocampal morphology in mutants (Figures 7D and 7E). To consolidate this claim, pregnant mice were treated with XAV-939, a substance with similar effect as ICG-001 (Mutch et al., 2010). As expected, XAV-939 treatment reproduced the Wnt inhibition-dependent rescue of cortical anomalies in dcKO mutants (Figures S7I–S7O).

Finally, we compared the transcriptome of cortices from control and dcKO embryos, which were treated with either Veh or Wnt inhibitor (Table S5, Figures 7F and 7G). Treatment with Wnt inhibitor decreases the expression of proliferation- and Wnt-related genes that are upregulated in dcKO embryos (Figures 7F and 7G).

Taken together, these results demonstrate that loss of BAF complexes during late cortical neurogenesis leads to aberrant enhancement of Wnt signaling activity and causes increased NSC proliferation-related defects similar to those observed after Wnt/β-catenin overexpression (Chenn and Walsh, 2002; Machon et al., 2007). These findings demonstrate that BAF complexes are required for proper hippocampal development through appropriate suppression of Wnt signaling in late developmental stages of the pallium.

DISCUSSION

In this study, we present evidence for the involvement of chromatin-remodeling BAF complexes in the regulation of global gene expression and epigenetic programs during late cortical neurogenesis. We showed that specific interactions of BAF155/BAF170 subunits with H3K27 and H3K4 demethylases possibly potentiate their activity during corticogenesis. During late development, loss of H3K27me3 and H3K4me2 marks on regulatory regions of distinct sets of genes potentiates disinhibition of transcription of RG- and neuronal differentiation-related genes, and suppresses NE-, Wnt signaling-, cell cycle-, and proliferation-related genes, respectively (Figure 5I). Thus, BAF complexes act both as activators and as repressors to regulate global epigenetic and gene expression programs during late corticogenesis and hippocampus development.

BAF155/BAF170-Dependent Maintenance of RG Cell Fate during Late Cortical Neurogenesis

Cortical neurogenesis comprises three main phases: (1) an expansion phase, characterized by symmetric division of NEs and expansion of the proliferative cell population; (2) a transition period during which NEs differentiate into RGs via asymmetric divisions to generate neurons as well as basal progenitors; and (3) a terminal phase during which progenitors undergo a terminal symmetric division to generate neurons and become quiescent (Dehay and Kennedy, 2007; Gotz and Huttner, 2005; Kriegstein and Alvarez-Buylla, 2009; Martynoga et al., 2012).

A few molecular factors are known to regulate NE to RG transition and RG differentiation (Dehay and Kennedy, 2007; Gotz and Huttner, 2005; Kriegstein and

Figure 7. BAF Complexes Control Hippocampal Development by Suppressing Wnt Signaling Activity

(A) Schematic model of the molecular cascades underlying late stages of pallium development in WT and dcKO pallium.

(B) Rescue experimental paradigm with the Wnt inhibitor (WNTi, ICG-001).

(C–E) IF (C and D) and quantitative (E) analyses of dcKO mutants at the indicated stages, showing the effects of treatment with ICG-001 on pools of PAX6^{+} NSCs (C and E), CASP3^{+} apoptotic cells (C and E), and ZBTB20^{+} (D and E) and PROX1^{+} neurons (E) in the developing hippocampus.

(F and G) Expression of Wnt (F) and Proliferation (G)-related genes in control, vehicle (Veh), WNTi-treated pallium.

(H) A proposed model showing how loss of BAF155 and BAF170 in dcKO mutants controls epigenetic and neural gene expression programs in proliferation and neuronal differentiation of the pallium in late developmental stages.

Values are presented as means ± SEMs (*$p < 0.05$; **$p < 0.01$, ***$p < 0.005$; ****$p < 0.0001$). Experimental replicates (n) = 4 (E, F, and G). Scale bars represent 100 μm (C, D, and F) and 50 μm (C).

Alvarez-Buylla, 2009; Martynoga et al., 2012). For example, ablation of *Fgf10* delayed RG differentiation during early corticogenesis, whereas NE fate seemed unaffected (Sahara and O'Leary, 2009). It is thus conceivable that, at later stages, other mechanisms may be required in limiting NSC fate to allow neuronal differentiation.

We showed that elimination of the BAF complex, by deleting the two scaffolding subunits BAF155 and BAF170 in cortex from E14.5 onward, results in a loss of RG fate hallmarks (diminished expression of astroglial and adherens junction markers), accompanied by gain of NE features (activation of tight junction and proliferation genes) (Figure 5H). Phenotypically, in the *dcKO* pallium, we found an overactive progenitor proliferation through symmetric divisions (a feature of NEs), instead of the typical predominant late-stage asymmetric division to produce one RG and a neuron or an IP. This leads to overproduction of NSCs at the expense of their derivatives (IPs and upper-layer neurons), and instigating the decreased radial cortical thickness and hypoplasia of the hippocampus in the dcKO mutants (Figure 5H).

Altogether, our results demonstrate that the chromatin-remodeling BAF complex is a crucial factor for ensuring the suppression of NE fate in the late neurogenic phase of corticogenesis.

BAF Complexes Control NSC Proliferation and Differentiation in Early and Late Embryonic Stages via Distinct Epigenetic Mechanisms

Discrete histone marks activate or inhibit gene expression programs that regulate neural development. Modifications such as H3K4me2/3 and H3K27Me2/3, regulated by their corresponding histone lysine methyltransferases (KMTs) and demethylases (KDMs), are associated with transcriptional activation and repression respectively.

H3K4 is commonly targeted by numerous KMTs and KDMs. In pluripotent ESCs, H3K4me2 marks signaling pathway genes that are required for the transition of neural progenitors to mature neurons (Zhang et al., 2012). H3K4me2 marks are established mainly by KMT2C/D methyltransferases and are removed by the KDM1 (LSD1) demethylase (Shi et al., 2004), which we found to be highly expressed in late cortical progenitors (Figure S1). Interestingly, LSD1 is also highly expressed in late progenitors in the developing mouse retina, and its inhibition blocks the differentiation of rod photoreceptors during late developmental stages (Popova et al., 2016).

Our earlier work indicated that BAF complexes interact with the H3K27 demethylases KDM6A/B to promote cell proliferation and neuronal differentiation in early cortical development (Narayanan et al., 2015). Accordingly, loss of BAF complexes in early corticogenesis results in a global increase in repressive H3K27me3 marks and downregulation of genes important for progenitor proliferation and differentiation. These two outcomes following ablation of BAF complexes during late development suggest a dual function of BAF complexes in activating neuronal differentiation genes and suppressing proliferation-related pathways that may reflect independent processes mediated by distinct BAF complex cofactors. As in early stages, BAF complexes possibly induce neuronal differentiation by interacting with KDM6A/B to remove inactivating H3K27me3 marks on loci of neuronal differentiation genes (Figure 7H). In parallel, however, BAF complexes inhibit cell amplification, probably by interacting with KDM1A, and potentiate its demethylase activity in H3K4me2 removal at genomic loci of genes involved in Wnt signaling, mitotic cell cycling, and proliferation (Figure 7H).

Based on these data, we propose that, during late pallium development, endogenous BAF complexes associate with the coactivators KDM6A/B to promote neuronal differentiation, while inhibiting cell proliferation via KDM1A recruitment.

BAF Complexes Suppress Wnt Signaling Activity

During cortical neurogenesis, temporal differentiation of NSCs leads to generation of cohorts of neurons with distinct layer identities, a process that depends on multiple regulatory pathways. Wnt signaling regulates the switch between proliferation and differentiation of cortical progenitors. Accordingly, ablation of *β-Catenin* or *Lrp6* (Wnt co-receptor) causes early cell cycle exit and premature differentiation of RGs into IPs and neurons (Draganova et al., 2015; Machon et al., 2007; Woodhead et al., 2006; Zhou et al., 2006). Conversely, persistent expression of β-catenin suppresses progenitor exit from mitosis, causing hyper-proliferation of NSCs through excessive symmetric division that consequently delays generation of $TBR2^{+}$ IPs and neuronal differentiation in the pallium (Chenn and Walsh, 2002; Machon et al., 2007; Mutch et al., 2010; Wrobel et al., 2007). Interestingly, these phenotypes are reminiscent of the observed abnormalities in the *dcKO* cortex.

Previous studies demonstrated that, in mammalian non-neural cells, the core BAF subunit BRG1 positively regulates Wnt signaling at distinct levels (e.g., exerting a control of genes encoding for Wnt receptors and also modulating β-catenin-dependent transcriptional activity) (Barker et al., 2001; Griffin et al., 2011). Surprisingly, upon loss of BAF155 and BAF170 in late cortical progenitors, multiple components and targets of the canonical Wnt/β-catenin signaling were upregulated, suggesting that the SWI/SNF complex can act to control the Wnt/β-catenin signaling pathway in a tissue- and context-dependent manner. Pharmacological inhibition of Wnt/β-catenin signaling rescued the observed defects in cell proliferation, cell survival, and restored hippocampal morphology in the dcKO mutants,

hence making us posit that BAF (SWI/SNF) complexes negatively regulate Wnt signaling during late cortical neurogenesis.

Altogether, our results indicate that the chromatin remodeler BAF plays a crucial role in late-stage development of mammalian cortex in two distinct ways. On one hand, BAF complexes induce heterochromatin formation at loci of cell cycle-, proliferation-, and Wnt-related genes, thereby suppressing their expression; and on the other, they facilitate the expression of neural differentiation-related genes by establishing euchromatin at related genomic regions. Together, these activities ensure the generation of appropriate numbers of NSCs and neurons in late cortical development.

EXPERIMENTAL PROCEDURES

Transgenic Lines, Plasmids, and Antibodies

Animals were handled in accordance with the German Animal Protection Law. A list of transgenic lines, plasmids, and antibodies with detailed descriptions is provided in Supplemental Information.

Mass Spectrometry, CoIP, ChIP-Seq, and RNA-Seq

Detailed descriptions were provided previously (Narayanan et al., 2015) and can be found in Supplemental Information.

ISH, IF, 3D Reconstruction Spindle Angle Analysis, and Cell-Cycle Index

ISH, IF, 3D reconstruction, and determination of cell-cycle index were performed as previously described (Bachmann et al., 2016; Tuoc et al., 2013). The spindle angle analysis is described in Supplemental Information.

In Vivo Pharmacological Treatment and *In Vivo* β-Catenin Transcriptional Activity Assay

A detailed description of treatment and assay are provided in Supplemental Information.

Imaging, Quantification, Statistical Analysis, and Data Availability

Images were captured by confocal fluorescence microscopy (TCS SP5, Leica) and analyzed using an Axio Imager M2 (Zeiss) with a Neurolucida system. Images were further processed with Adobe Photoshop. IF signal intensities were quantified using ImageJ software, as described previously (Narayanan et al., 2015; Tuoc and Stoykova, 2008). The statistical quantification was carried out as average from at least three biological replicates. Details of statistical analyses and descriptions for histological experiments are presented in Table S6 and in Supplemental Information.

ACCESSION NUMBERS

All RNA-seq and ChIP-seq data have been deposited in GEO under accession number GEO: GSE106711.

SUPPLEMENTAL INFORMATION

Supplemental Information includes Supplemental Experimental Procedures, seven figures, six tables, and one video and can be found with this article online at https://doi.org/10.1016/j.stemcr.2018.04.014.

AUTHOR CONTRIBUTIONS

H.N. performed most characterization of dcKO phenotypes; C.K. and A.F. generated RNA-seq and ChIP-seq data; M.P. performed the protein-protein interaction study; L.P., G.S., and J.R. contributed to histological analyses; K.A.K. characterized hGFAP-Cre_ROSA-dtTOM mouse line; C.K. performed ChIP-qPCR; M.S.S. performed qPCR; J.F.S., R.H.S., U.T., and A.S. provided research tools and transgenic lines and contributed to discussions; T.T. conceived, supervised, and wrote the manuscript; C.K. designed all experiments related to RNA-seq and ChIP-seq and their confirmation, analyzed all related data, and wrote the corresponding parts of the manuscript; J.F.S., A.S., and A.F. offered suggestions for the study. The authors declare no competing financial interests.

ACKNOWLEDGMENTS

We acknowledge T. Huttanus and H. Fett for their expert animal care and support. We also thank A. Messing, A. Nave, A.P. McMahon, A. Jones, and O. Machon for providing reagents; A.B. Tonchev for helpful discussions; M. Kessel for his support; and G. Hafner for his assistance with 3D analysis. This work was supported by the Universitätsmedizin Göttingen (T.T.), TU432/1-1, TU432/1-3 DFG grants (T.T.), T278/29576/2017 Schram-Stiftung grant (T.T.), and DFG-CNMPB (T.T., J.S., A.S., and A.F.).

Received: January 8, 2018
Revised: April 16, 2018
Accepted: April 17, 2018
Published: May 17, 2018

REFERENCES

Aaku-Saraste, E., Hellwig, A., and Huttner, W.B. (1996). Loss of occludin and functional tight junctions, but not ZO-1, during neural tube closure–remodeling of the neuroepithelium prior to neurogenesis. Dev. Biol. *180*, 664–679.

Attanasio, C., Nord, A.S., Zhu, Y., Blow, M.J., Biddie, S.C., Mendenhall, E.M., Dixon, J., Wright, C., Hosseini, R., Akiyama, J.A., et al. (2014). Tissue-specific SMARCA4 binding at active and repressed regulatory elements during embryogenesis. Genome Res. *24*, 920–929.

Bachmann, C., Nguyen, H., Rosenbusch, J., Pham, L., Rabe, T., Patwa, M., Sokpor, G., Seong, R.H., Ashery-Padan, R., Mansouri, A., et al. (2016). mSWI/SNF (BAF) complexes are indispensable for the neurogenesis and development of embryonic olfactory epithelium. PLoS Genet. *12*, e1006274.

Barker, N., Hurlstone, A., Musisi, H., Miles, A., Bienz, M., and Clevers, H. (2001). The chromatin remodelling factor Brg-1 interacts with beta-catenin to promote target gene activation. EMBO J. *20*, 4935–4943.

Chenn, A., and Walsh, C.A. (2002). Regulation of cerebral cortical size by control of cell cycle exit in neural precursors. Science *297*, 365–369.

Dehay, C., and Kennedy, H. (2007). Cell-cycle control and cortical development. Nat. Rev. Neurosci. *8*, 438–450.

Draganova, K., Zemke, M., Zurkirchen, L., Valenta, T., Cantù, C., Okoniewski, M., Schmid, M.T., Hoffmans, R., Götz, M., Basler, K., et al. (2015). Wnt/beta-catenin signaling regulates sequential fate decisions of murine cortical precursor cells. Stem Cells *33*, 170–182.

Gao, P., Postiglione, M.P., Krieger, T.G., Hernandez, L., Wang, C., Han, Z., Streicher, C., Papusheva, E., Insolera, R., Chugh, K., et al. (2014). Deterministic progenitor behavior and unitary production of neurons in the neocortex. Cell *159*, 775–788.

Gotz, M., and Huttner, W.B. (2005). The cell biology of neurogenesis. Nat. Rev. Mol. Cell Biol. *6*, 777–788.

Griffin, C.T., Curtis, C.D., Davis, R.B., Muthukumar, V., and Magnuson, T. (2011). The chromatin-remodeling enzyme BRG1 modulates vascular Wnt signaling at two levels. Proc. Natl. Acad. Sci. USA *108*, 2282–2287.

Hartfuss, E., Galli, R., Heins, N., and Gotz, M. (2001). Characterization of CNS precursor subtypes and radial glia. Dev. Biol. *229*, 15–30.

Hirabayashi, Y., and Gotoh, Y. (2010). Epigenetic control of neural precursor cell fate during development. Nat. Rev. Neurosci. *11*, 377–388.

Kriegstein, A., and Alvarez-Buylla, A. (2009). The glial nature of embryonic and adult neural stem cells. Annu. Rev. Neurosci. *32*, 149–184.

Kruidenier, L., Chung, C.W., Cheng, Z., Liddle, J., Che, K., Joberty, G., Bantscheff, M., Bountra, C., Bridges, A., Diallo, H., et al. (2012). A selective jumonji H3K27 demethylase inhibitor modulates the proinflammatory macrophage response. Nature *488*, 404–408.

Machon, O., Backman, M., Machonova, O., Kozmik, Z., Vacik, T., Andersen, L., and Krauss, S. (2007). A dynamic gradient of Wnt signaling controls initiation of neurogenesis in the mammalian cortex and cellular specification in the hippocampus. Dev. Biol. *311*, 223–237.

Martynoga, B., Drechsel, D., and Guillemot, F. (2012). Molecular control of neurogenesis: a view from the mammalian cerebral cortex. Cold Spring Harb. Perspect. Biol. *4*. https://doi.org/10.1101/cshperspect.a008359.

Mutch, C.A., Schulte, J.D., Olson, E., and Chenn, A. (2010). Beta-catenin signaling negatively regulates intermediate progenitor population numbers in the developing cortex. PLoS One *5*, e12376.

Narayanan, R., Pirouz, M., Kerimoglu, C., Pham, L., Wagener, R.J., Kiszka, K.A., Rosenbusch, J., Seong, R.H., Kessel, M., Fischer, A., et al. (2015). Loss of BAF (mSWI/SNF) complexes causes global transcriptional and chromatin state changes in forebrain development. Cell Rep. *13*, 1842–1854.

Nguyen, H., Sokpor, G., Pham, L., Rosenbusch, J., Stoykova, A., Staiger, J.F., and Tuoc, T. (2016). Epigenetic regulation by BAF (mSWI/SNF) chromatin remodeling complexes is indispensable for embryonic development. Cell Cycle *15*, 1317–1324.

Popova, E.Y., Pinzon-Guzman, C., Salzberg, A.C., Zhang, S.S.M., and Barnstable, C.J. (2016). LSD1-mediated demethylation of H3K4me2 is required for the transition from late progenitor to differentiated mouse rod photoreceptor. Mol. Neurobiol. *53*, 4563–4581.

Sahara, S., and O'Leary, D.D. (2009). Fgf10 regulates transition period of cortical stem cell differentiation to radial glia controlling generation of neurons and basal progenitors. Neuron *63*, 48–62.

Shi, Y., Lan, F., Matson, C., Mulligan, P., Whetstine, J.R., Cole, P.A., and Casero, R.A. (2004). Histone demethylation mediated by the nuclear amine oxidase homolog LSD1. Cell *119*, 941–953.

Sun, G., Alzayady, K., Stewart, R., Ye, P., Yang, S., Li, W., and Shi, Y. (2010). Histone demethylase LSD1 regulates neural stem cell proliferation. Mol. Cell. Biol. *30*, 1997–2005.

Takahashi, T., Nowakowski, R.S., and Caviness, V.S., Jr. (1995). The cell cycle of the pseudostratified ventricular epithelium of the embryonic murine cerebral wall. J. Neurosci. *15*, 6046–6057.

Tuoc, T.C., Boretius, S., Sansom, S.N., Pitulescu, M.E., Frahm, J., Livesey, F.J., and Stoykova, A. (2013). Chromatin regulation by BAF170 controls cerebral cortical size and thickness. Dev. Cell *25*, 256–269.

Tuoc, T.C., Pavlakis, E., Tylkowski, M.A., and Stoykova, A. (2014). Control of cerebral size and thickness. Cell. Mol. Life Sci. *71*, 3199–3218.

Tuoc, T.C., Radyushkin, K., Tonchev, A.B., Pinon, M.C., Ashery-Padan, R., Molnar, Z., Davidoff, M.S., and Stoykova, A. (2009). Selective cortical layering abnormalities and behavioral deficits in cortex-specific Pax6 knock-out mice. J. Neurosci. *29*, 8335–8349.

Tuoc, T.C., and Stoykova, A. (2008). Trim11 modulates the function of neurogenic transcription factor Pax6 through ubiquitin-proteosome system. Genes Dev. *22*, 1972–1986.

Woodhead, G.J., Mutch, C.A., Olson, E.C., and Chenn, A. (2006). Cell-autonomous beta-catenin signaling regulates cortical precursor proliferation. J. Neurosci. *26*, 12620–12630.

Wrobel, C.N., Mutch, C.A., Swaminathan, S., Taketo, M.M., and Chenn, A. (2007). Persistent expression of stabilized beta-catenin delays maturation of radial glial cells into intermediate progenitors. Dev. Biol. *309*, 285–297.

Yao, B., Christian, K.M., He, C., Jin, P., Ming, G.L., and Song, H. (2016). Epigenetic mechanisms in neurogenesis. Nat. Rev. Neurosci. *17*, 537–549.

Zhang, J., Parvin, J., and Huang, K. (2012). Redistribution of H3K4me2 on neural tissue specific genes during mouse brain development. BMC Genomics *13* (*Suppl 8*), S5.

Zhou, C.J., Borello, U., Rubenstein, J.L., and Pleasure, S.J. (2006). Neuronal production and precursor proliferation defects in the neocortex of mice with loss of function in the canonical Wnt signaling pathway. Neuroscience *142*, 1119–1131.

Stem Cell Reports, Volume *10*

Supplemental Information

Epigenetic Regulation by BAF Complexes Limits Neural Stem Cell Proliferation by Suppressing Wnt Signaling in Late Embryonic Development

Huong Nguyen, Cemil Kerimoglu, Mehdi Pirouz, Linh Pham, Kamila A. Kiszka, Godwin Sokpor, M. Sadman Sakib, Joachim Rosenbusch, Ulrike Teichmann, Rho H. Seong, Anastassia Stoykova, Andre Fischer, Jochen F. Staiger, and Tran Tuoc

SUPPLEMENTAL DATA

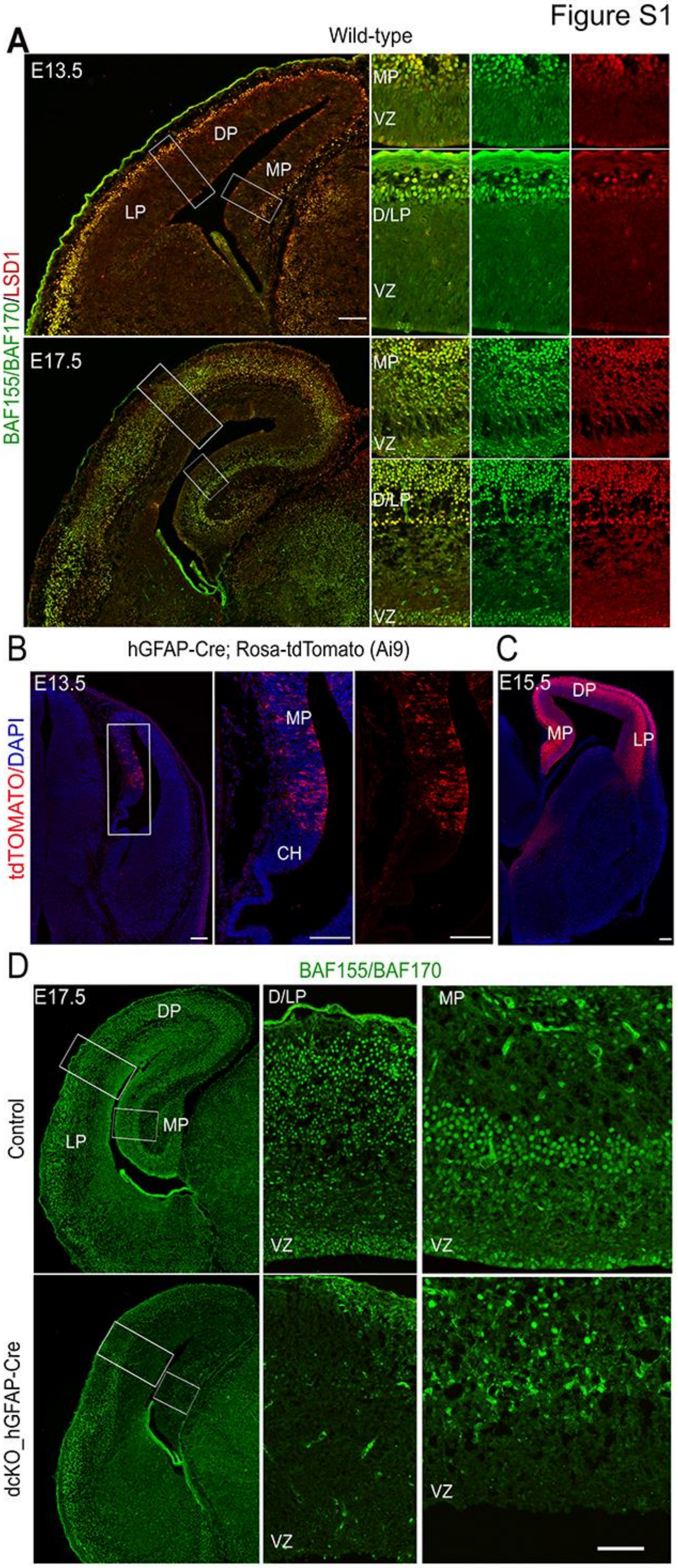

Figure S1 (related to figure 1). Co-expression of BAF155/BAF170 with H3K4 and H3K27 demethylases in the developing pallium; hGFAP-Cre activity in the developing pallium; and expression of BAF155/BAF170 in the dcKO_*hGFAP*-Cre pallium.

(A) Double-label IF analysis with anti-BAF155/BAF170 (green) and anti-LSD1/KDM1A (red) antibodies, showing co-expression of BAF155/BAF170 with LSD1/KDM1A in pallium cells. Co-expression of BAF155/BAF170 with UTX/KDM6A or JMJD3/ KDM6B in the developing forebrain was analyzed previously (Narayanan et al., 2015). (B, C) Immunostaining for dtTomato on cortical sections from hGFAP-Cre; Rosa-tdTomato (Ai9) embryos at E13.5 (B) and E15.5 (C). (B) The right panels are higher-magnification images from the fields in the MP indicated by white frames. By E13.5, recombination is restricted in MP but absent from D/LP and cortical hem (CH) (B). From E15.5 onward, Cre recombination was detected in entire pallium (C). (D) Compared to controls, expression of BAF155 and BAF170 was largely lost in the dcKO pallium, as shown by IF analysis with anti-BAF155/BAF170 antibodies. Abbreviations: VZ, ventricular zone; MP, medial pallium; DP, dorsal pallium; LP, lateral pallium; CH, cortical hem. Scale bars = 100 μm.

Figure S2

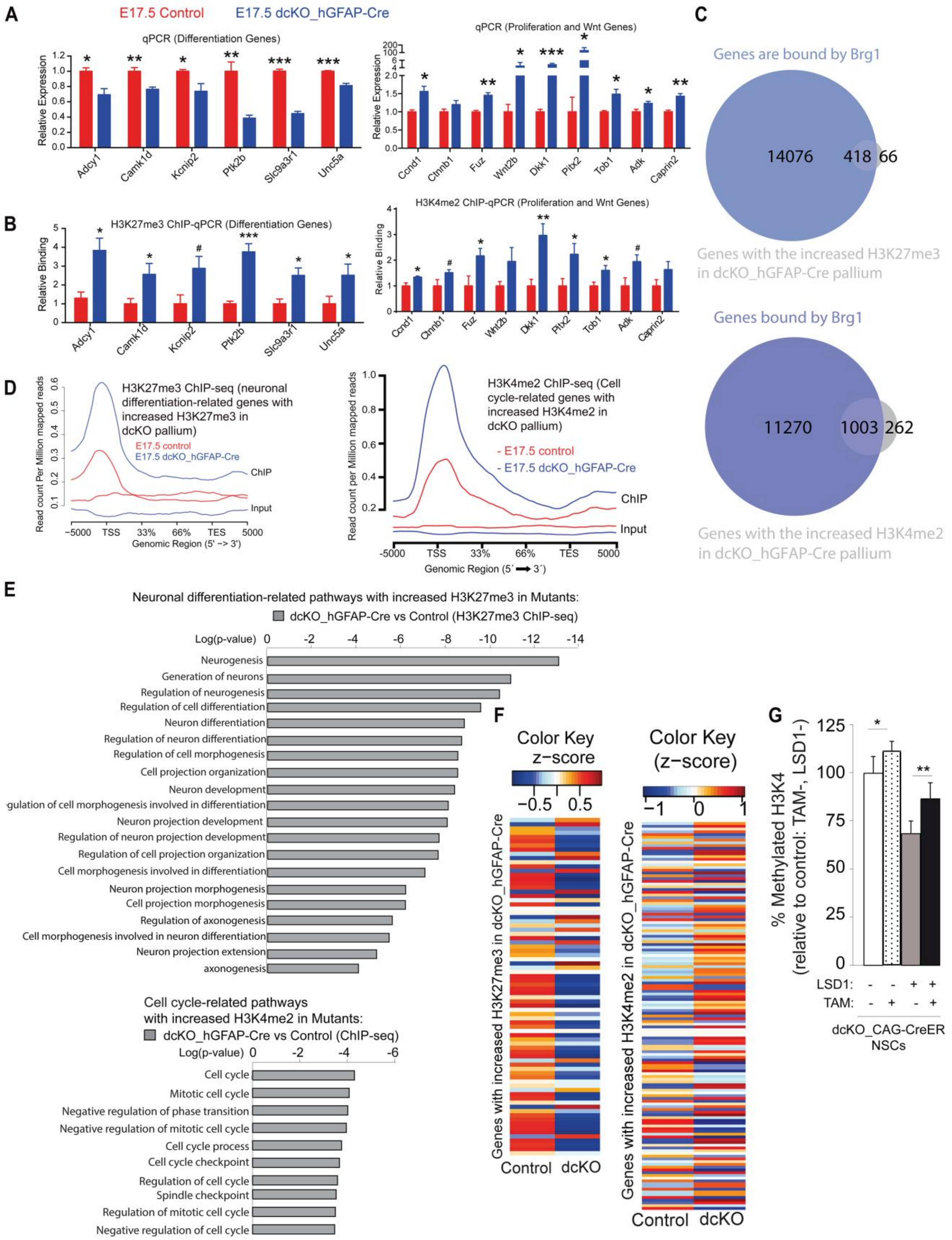
A
E17.5 Control
E17.5 dcKO_hGFAP-Cre
qPCR (Differentiation Genes)
Relative Expression
Adcy1
Camk1d
Kcnip2
Ptk2b
Slc9a3r1
Unc5a
qPCR (Proliferation and Wnt Genes)
Ccnd1
Ctnnb1
Fuz
Wnt2b
Dkk1
Pitx2
Tob1
Adk
Caprin2
B
H3K27me3 ChIP-qPCR (Differentiation Genes)
Relative Binding
H3K4me2 ChIP-qPCR (Proliferation and Wnt Genes)
C
Genes are bound by Brg1
14076
418
66
Genes with the increased H3K27me3 in dcKO_hGFAP-Cre pallium
Genes bound by Brg1
11270
1003
262
Genes with the increased H3K4me2 in dcKO_hGFAP-Cre pallium
D
H3K27me3 ChIP-seq (neuronal differentiation-related genes with increased H3K27me3 in dcKO pallium)
E17.5 control
E17.5 dcKO_hGFAP-Cre
ChIP
Input
Read count Per Million mapped reads
Genomic Region (5' -> 3')
H3K4me2 ChIP-seq (Cell cycle-related genes with increased H3K4me2 in dcKO pallium)
- E17.5 control
- E17.5 dcKO_hGFAP-Cre
E
Neuronal differentiation-related pathways with increased H3K27me3 in Mutants:
dcKO_hGFAP-Cre vs Control (H3K27me3 ChIP-seq)
Log(p-value)
Neurogenesis
Generation of neurons
Regulation of neurogenesis
Regulation of cell differentiation
Neuron differentiation
Regulation of neuron differentiation
Regulation of cell morphogenesis
Cell projection organization
Neuron development
gulation of cell morphogenesis involved in differentiation
Neuron projection development
Regulation of neuron projection development
Regulation of cell projection organization
Cell morphogenesis involved in differentiation
Neuron projection morphogenesis
Cell projection morphogenesis
Regulation of axonogenesis
Cell morphogenesis involved in neuron differentiation
Neuron projection extension
axonogenesis
Cell cycle-related pathways with increased H3K4me2 in Mutants:
dcKO_hGFAP-Cre vs Control (ChIP-seq)
Cell cycle
Mitotic cell cycle
Negative regulation of phase transition
Negative regulation of mitotic cell cycle
Cell cycle process
Cell cycle checkpoint
Regulation of cell cycle
Spindle checkpoint
Regulation of mitotic cell cycle
Negative regulation of cell cycle
F
Color Key z-score
Genes with increased H3K27me3 in dcKO_hGFAP-Cre
Control
dcKO
Color Key (z-score)
Genes with increased H3K4me2 in dcKO_hGFAP-Cre
G
% Methylated H3K4 (relative to control: TAM-, LSD1-)
LSD1:
TAM:
dcKO_CAG-CreER NSCs

Figure S2 (related to figures 1, 2, 4). Characterization of H3K27me3 and H3K4me2 marks in dcKO_hGFAP-Cre cortex.

(A) qPCR confirmation of selected neuronal differentiation-related genes downregulated, proliferation- and Wnt-related genes upregulated in dcKO_*hGFAP*-Cre embryos at E17.5 (B) ChIP-qPCR confirmation of H3K27me3 levels at selected neuronal differentiation-related genes downregulated and H3K4me2 levels at selected proliferation and Wnt-related genes upregulated in dcKO_*hGFAP*-Cre embryos at E17.5. (C) Overlap between genes bound by BRG1 (GSE37151; Attanasio et al., 2014) and those with increased H3K27me3 and those with increased H3K4me2 in dcKO embryos (hypergeometric test: *p*-value < 0.0001). (D) General profile plots of H3K27me3 at neuronal differentiation-related genes with increased H3K27me3 and H3K4me2 at cell cycle-related genes with increased H3K4me2 in dcKO_*hGFAP*-Cre embryos (*p*-value < 0.0001, Student's t-test). (E) Neuronal differentiation-related genes have increased H3K27me3 and Cell cycle-related genes have increased H3K4me2 (*p*-value < 0.01) in dcKO_*hGFAP*-Cre embryos at E17.5. (F) Heatmaps depicting the expression changes in neural differentiation-related genes that have increased H3K27me3 and in cell cycle-related genes that have increased H3K4me2 in dcKO_*hGFAP*-Cre embryos at E17.5. (G) In the LSD1 demethylase activity quantification assay, cultured dcKO_CAG-Cre NSCs were nucleofected with a mammalian expression vector for LSD1/KDM1A. The Cre-mediated deletion of BAF155 and BAF170 alleles was induced by adding TAM. Compared to control (LSD1-, TAM-, white bar), overexpression of LSD1 (LSD1+, TAM-, grey bar) decreased the methylated H3K4. In absence of BAF complexes (LSD1+, TAM, black bar) LSD1 displayed its low H3K4 demethylase activity. Values are presented as means ± SEMs (**p*-value < 0.05, ****p*-value < 0.01, ****p*-value < 0.005). Experimental replicates (n) = 4 (A, B), 6 (G).

Figure S3

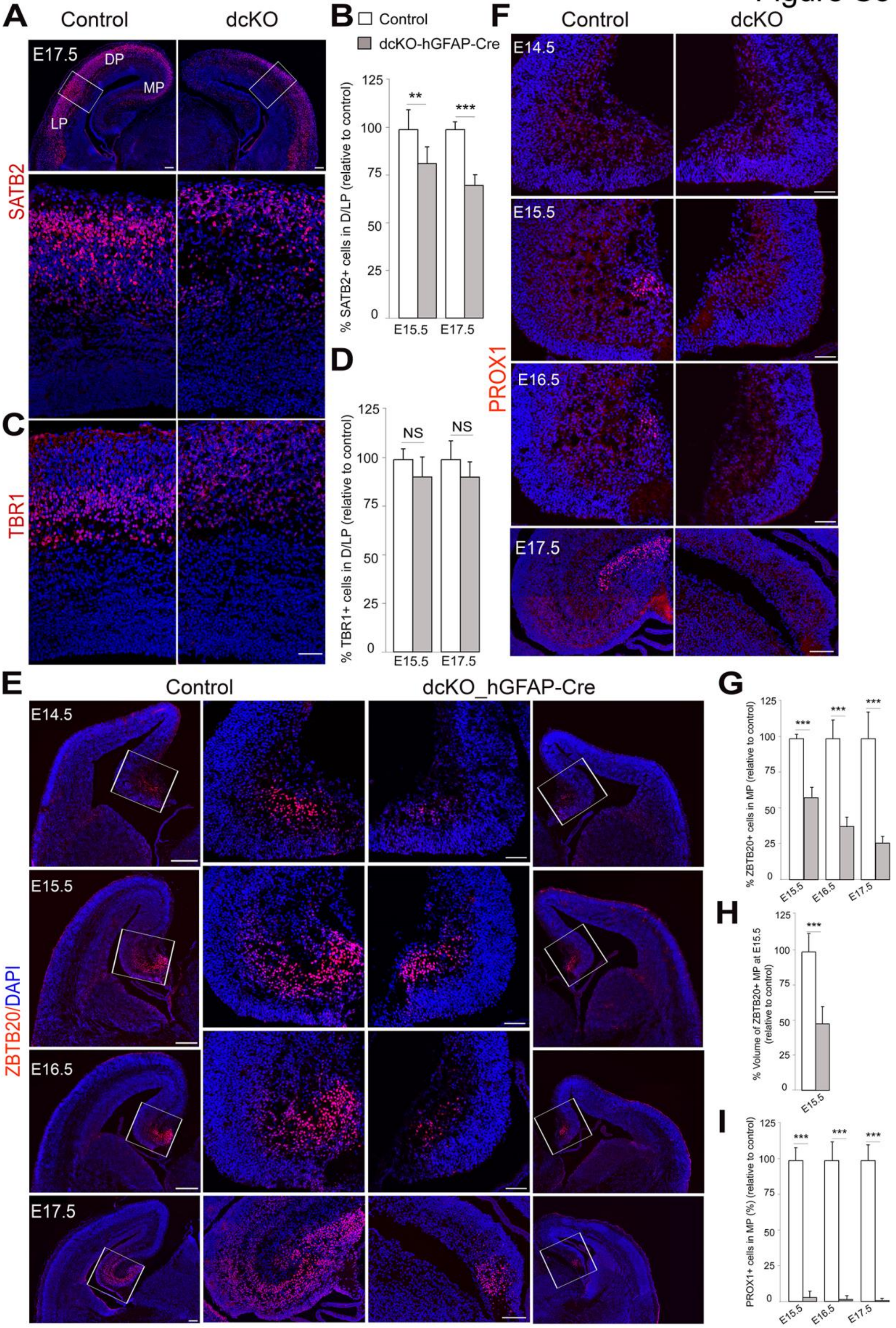
A
Control
dcKO
E17.5
DP
MP
LP
SATB2
B
Control
dcKO-hGFAP-Cre
% SATB2+ cells in D/LP (relative to control)
125
100
75
50
25
0
**
***
E15.5
E17.5
C
TBR1
D
% TBR1+ cells in D/LP (relative to control)
NS
NS
E15.5
E17.5
F
Control
dcKO
PROX1
E14.5
E15.5
E16.5
E17.5
E
Control
dcKO_hGFAP-Cre
ZBTB20/DAPI
E14.5
E15.5
E16.5
E17.5
G
% ZBTB20+ cells in MP (relative to control)
***
***
***
E15.5
E16.5
E17.5
H
% Volume of ZBTB20+ MP at E15.5 (relative to control)
***
E15.5
I
PROX1+ cells in MP (%) (relative to control)
***
***
***
E15.5
E16.5
E17.5

Figure S3 (related to figures 2, 3). BAF155 and BAF170 are essential for neurogenesis in the developing cortex and hippocampus.

(A–B) IF (A, C) and quantitative (B, D) analyses of the neurogenesis phenotype using the late-born neuronal marker SATB2 (A, B) and early-born neuronal marker TBR1 (C, D). (E - I) IF (E, F) and quantitative (G-I) analyses for the neuronal markers ZBTB20 in the hippocampus (E, G, H) and PROX1 in the dentate gyrus (F, I) revealed that hippocampal sections of the MP of mutants have a reduced number of ZBTB20$^+$ neurons and PROX1$^+$ neurons at E15.5–E17.5 compared to controls. (H) Quantification of the ZBTB20$^+$ hippocampal volume performed across the entire hippocampus using 3D reconstruction (see also Figure S5A and Movie S1). Values are expressed as means ± SEMs (*P < 0.05; **P < 0.01; ***P < 0.005). Experimental replicates (n) = 6 (B, D, G, I), 4 (H). Abbreviations: MP, medial pallium; DP, dorsal pallium; LP, lateral pallium. Scale bars = 100 μm (10x; A,E), 50 μm (25x; A,C), and 50 μm (40x; E,F).

Figure S4

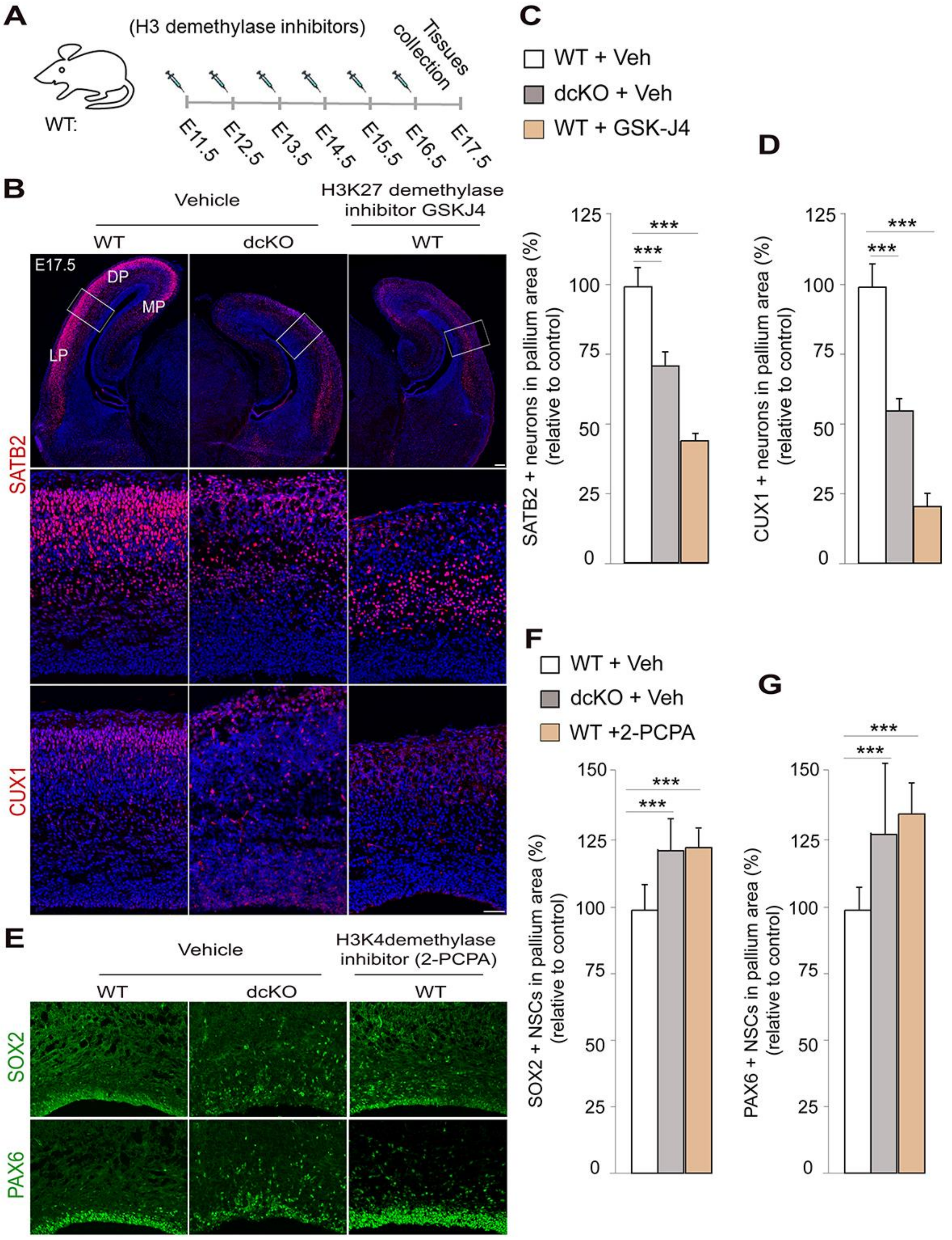
A
(H3 demethylase inhibitors)
Tissues collection
WT:
E11.5
E12.5
E13.5
E14.5
E15.5
E16.5
E17.5
B
Vehicle
H3K27 demethylase inhibitor GSKJ4
WT
dcKO
WT
E17.5
DP
MP
LP
SATB2
CUX1
C
WT + Veh
dcKO + Veh
WT + GSK-J4
D
SATB2 + neurons in pallium area (%) (relative to control)
CUX1 + neurons in pallium area (%) (relative to control)
E
Vehicle
H3K4demethylase inhibitor (2-PCPA)
WT
dcKO
WT
SOX2
PAX6
F
WT + Veh
dcKO + Veh
WT +2-PCPA
G
SOX2 + NSCs in pallium area (%) (relative to control)
PAX6 + NSCs in pallium area (%) (relative to control)
***

Figure S4 (related to figures 2, 4). Elevated level of H3K27me3 and H3K4me2 by inhibition of H3K27 and H3K4me2 demethylases caused the defect in neuronal differentiation in developing pallium.

(A) Experimental paradigms in which WT embryos were treated with H3K27 demethylase inhibitor GSK-J4 and H3K4 demethylase inhibitor 2-PCPA. (B–G) IF (B, E) and quantitative (C, D, F, G) analyses are to compare cortical phenotype of Veh-treated WT, Veh-dcKO and WT which is treated with GSK-J4 (a-d) or with 2-PCPA (e-g). (C, D) Statistical comparisons indicate that the increased level of H3K27me3 in cortices of dcKO embryos or GSK-J4 treated embryos decreased the number of late-born SATB2$^+$ (C) and CUX1$^+$ neurons (D) compared to Veh-treated WT. Likewise, the increased level of H3K4me2 in cortices of dcKO embryos or 2-PCPA treated embryos increased the number of SOX2$^+$ (F) and PAX6$^+$ NSCs (G) compared to Veh-treated WT. Values are expressed as means ± SEMs (*$P < 0.05$; **$P < 0.01$; ***$P < 0.005$). Experimental replicates (n) = 4 (C, D, F, G). Abbreviations: MP, medial pallium; DP, dorsal pallium; LP, lateral pallium. Scale bars = 100 μm (10x, B) and 50 μm (40x, B).

Figure S5

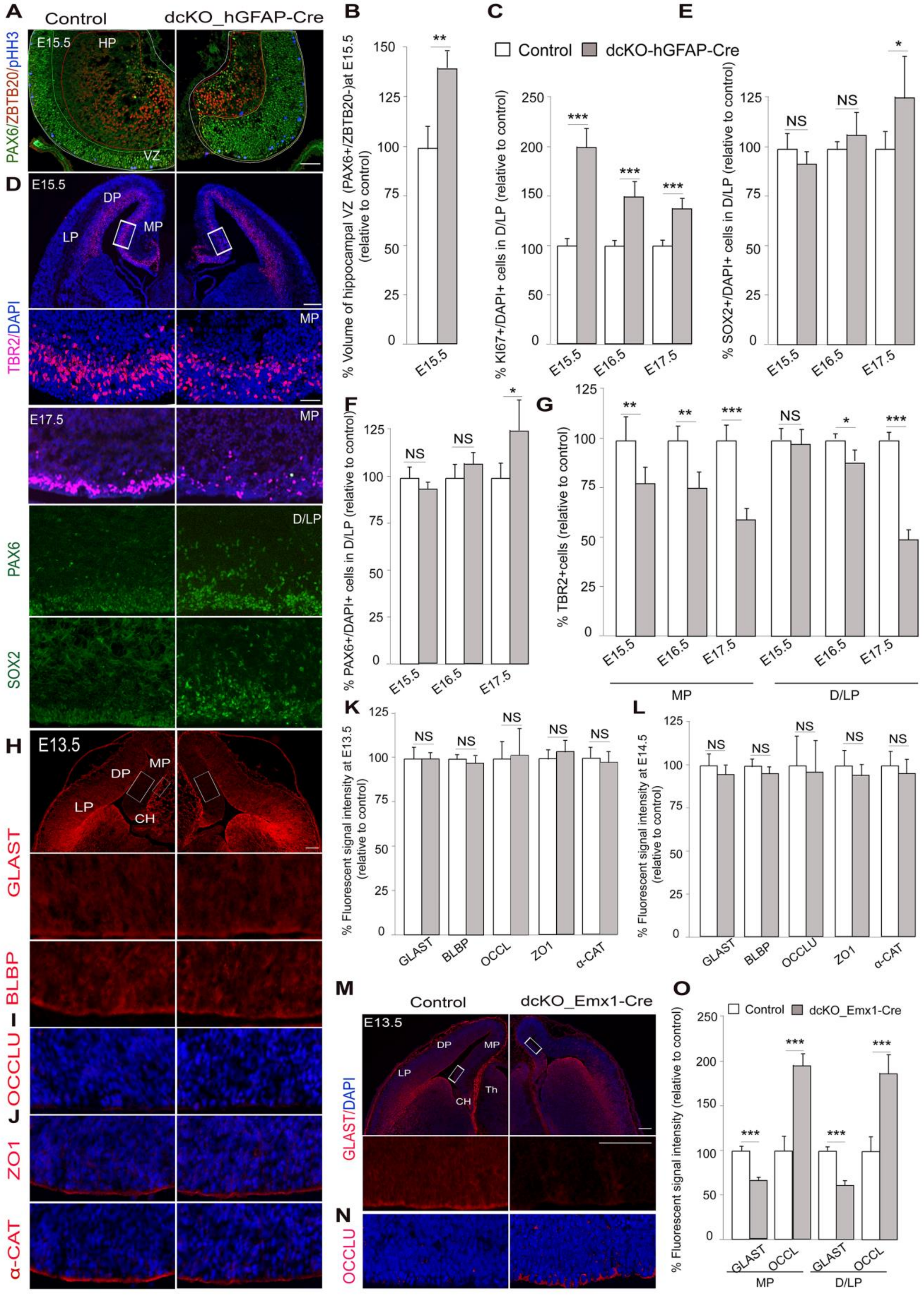
A
Control
dcKO_hGFAP-Cre
PAX6/ZBTB20/pHH3
E15.5
HP
VZ
B
% Volume of hippocampal VZ (PAX6+/ZBTB20-)at E15.5 (relative to control)
C
Control
dcKO-hGFAP-Cre
% KI67+/DAPI+ cells in D/LP (relative to control)
E
% SOX2+/DAPI+ cells in D/LP (relative to control)
D
E15.5
DP
MP
LP
TBR2/DAPI
E17.5
PAX6
D/LP
SOX2
F
% PAX6+/DAPI+ cells in D/LP (relative to control)
G
% TBR2+cells (relative to control)
MP
D/LP
H
E13.5
CH
GLAST
BLBP
I
OCCLU
J
ZO1
α-CAT
K
% Fluorescent signal intensity at E13.5 (relative to control)
L
% Fluorescent signal intensity at E14.5 (relative to control)
OCCL
OCCLU
M
Control
dcKO_Emx1-Cre
GLAST/DAPI
Th
N
O
dcKO_Emx1-Cre
% Fluorescent signal intensity (relative to control)
E15.5
E16.5
E17.5
NS

Figure S5 (related to figures 4, 5). Loss of BAF155 and BAF170 in dcKO mutants has a profound effect on the pool of NSCs, expression of adherens junction molecules.

(A) Representative images of triple IF analysis of PAX6 to visualize hippocampal VZ, ZBTB20 to mark hippocampal plate (HP) and PHH3 to label mitotic M-phase cells in our 3D reconstruction analysis. Total hippocampal volume was surrounded by white line. (B) Quantitative analyses of the volume of the hippocampal VZ ($PAX6^{+}/ZBTB20^{-}$) in the dcKO_*hGFAP*-Cre mutant and control at E15.5 (see also Movie S1 for 3D reconstruction analysis). (C–G) IF (D) and quantitative (C, E, F and G) analyses of the mitotic marker KI67 (C), RG markers PAX6 and SOX2 (D, E and F), and IP marker TBR2 in the MP and D/LP of dcKO_*hGFAP*-Cre mutants and control (G). Generally, the loss of BAF155 and BAF170 in dcKO_*hGFAP*-Cre mutants had a more profound effect on the pool of NSCs in the MP (see also Fig. 4E–L) than in the D/LP. It should be noted that the loss of BAF155 and BAF170 in the E15.5–E16.5 D/LP did not affect the pool of $PAX6^{+}/SOX2^{+}$ RGs; however, the number of $KI67^{+}$ active mitotic progenitors was already increased. Counting was done in selected frames, denoted by white boxes. (H-L) IF (H-J) and quantitative (K, L) analyses revealed that in contrast to later stages (E15.5. E16.5), there is no obvious difference in expression level of GLAST, BLBP, ZO1, α-CATENIN and OCCLUDIN between the control and dcKO_hGFAP-Cre pallium at E13.5 and E14.5. (M–O) Immunostaining of coronal sections from the control and dcKO_*Emx1*-Cre pallium at E13.5 for GLAST (M) and OCCLUDIN (N) revealed an altered cell identity from $GLAST^{high+}/OCCLUDIN^{-}$ RGs in controls to $GLAST^{low+}/OCCLUDIN^{high+}$ NEs in the dcKO_*Emx1*-Cre mutant. (O) Quantitative analyses of panels M–N are shown. Values are expressed as means ± SEMs (*$P < 0.05$; **$P < 0.01$; ***$P < 0.005$). Experimental replicates (n) = 4 (B), 6 (C, E, F, G, K, L, O). Abbreviations: MP, medial pallium; DP, dorsal pallium; LP, lateral pallium; HP, hippocampal plate; VZ, ventricular zone; CH, cortical hem; Th, thalamus. Scale bar = 100 μm.

Figure.S6

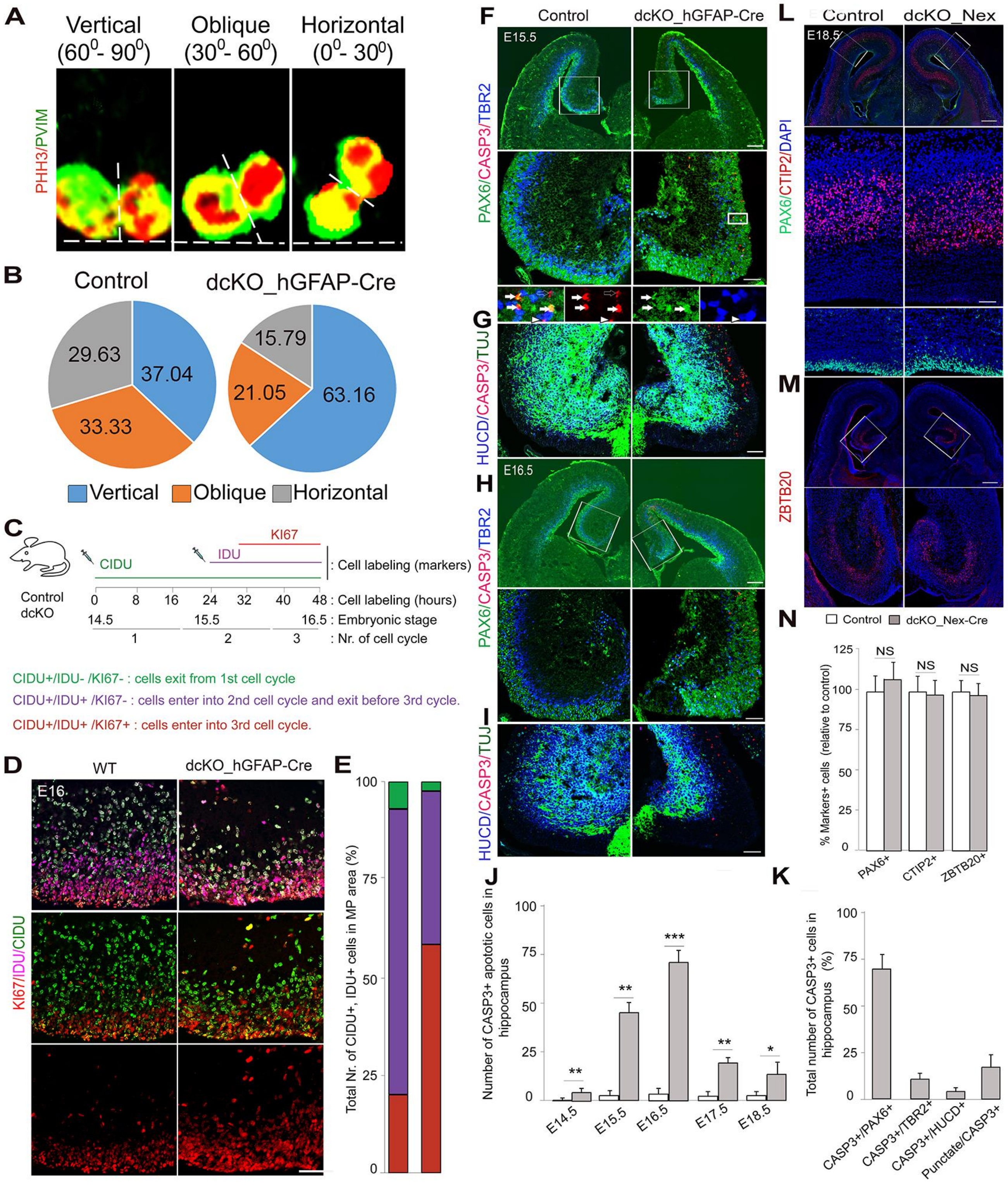
A
Vertical (60°- 90°)
Oblique (30°- 60°)
Horizontal (0°- 30°)
PHH3/PVIM
B
Control
dcKO_hGFAP-Cre
29.63
37.04
33.33
15.79
21.05
63.16
Vertical
Oblique
Horizontal
C
KI67
IDU
CIDU
: Cell labeling (markers)
Control
dcKO
0 8 16 24 32 40 48 : Cell labeling (hours)
14.5 15.5 16.5 : Embryonic stage
1 2 3 : Nr. of cell cycle
CIDU+/IDU- /KI67- : cells exit from 1st cell cycle
CIDU+/IDU+ /KI67- : cells enter into 2nd cell cycle and exit before 3rd cycle.
CIDU+/IDU+ /KI67+ : cells enter into 3rd cell cycle.
D
WT
dcKO_hGFAP-Cre
E16
KI67/IDU/CIDU
E
Total Nr. of CIDU+, IDU+ cells in MP area (%)
F
Control
dcKO_hGFAP-Cre
E15.5
PAX6/CASP3/TBR2
G
HUCD/CASP3/TUJ
H
E16.5
PAX6/CASP3/TBR2
I
HUCD/CASP3/TUJ
J
Number of CASP3+ apotic cells in hippocampus
E14.5
E15.5
E16.5
E17.5
E18.5
K
Total number of CASP3+ cells in hippocampus (%)
CASP3+/PAX6+
CASP3+/TBR2+
CASP3+/HUCD+
Punctate/CASP3+
L
Control
dcKO_Nex
E18.5
PAX6/CTIP2/DAPI
M
ZBTB20
N
Control
dcKO_Nex-Cre
% Markers+ cells (relative to control)
NS
PAX6+
CTIP2+
ZBTB20+

Figure S6 (related to figures 2-5). The spindle orientation, selective apoptosis of RGs in dcKO_hGFAP-Cre mutants and phenotypes of cortical neuron-specific dcKO_Nex-Cre mutants.

(A, B) IF analyses in control and dcKO mutants using antibodies against PHH3 and PVIM , to visualize the orientation of spindles (vertical, oblique, horizontal) (A), and quantitative analyses (B) indicate that loss of BAF155 and BAF170 induces proliferative, symmetric division. (C) Experimental paradigm for determining cell-cycle parameters and color scheme for immunolabeling of micrographs (in D). (D) Images show triple-label IHC of cortical sections with antibodies for CIDU (48-h labeling), IDU (24-h labeling) to mark both exited and cycling progenitors, KI67 to label proliferating progenitors at E16.5. (E) Statistical comparisons indicated a decreased number of cells, which exit from 1st and 2nd cell cycles and an increased number of progenitors, which enter the 3rd cycle in the medial pallium of dcKO embryos compared with control. (F-K) Triple IF for PAX6/TBR2/CASP3 and HuCD/TUBB3/CASP3 (F–I) and quantitative analyses (J, K) show that the developing hippocampus of the dcKO-*hGFAP*-Cre embryo has a high number of $CASP3^+$ apoptotic cells at E14.5–E18.5 (F–J). Notably, most $CASP3^+$ cells are immunoreactive for PAX6 (white filled arrows) or exhibit punctate forms (late phase of apoptosis, empty arrows), whereas less extended cells are $TBR2^+$ IPs and $HUCD^+/TUBB3^+$ neurons (F, H, K; arrowhead). Lower panels are higher-magnification images from the fields indicated by white rectangles. Noted that a similar image of single channel for Pax6 is shown in Figure 4F. (L–N) Phenotype analysis of the pallium from neuron-specific dcKO_*Nex*-Cre mutants. Immunostaining of coronal sections of E18.5 brains with antibodies specific for RGs (PAX6; L), layer 5 neurons (CTIP2; L) and hippocampal neurons (ZBTB20; M), and quantification of results in (L) showed no discernible differences in the number of immunopositive cells between the dcKO_*Nex*-Cre and control pallium. Values are presented as means ± SEMs (**P* < 0.05, ****P* < 0.01, ****P* < 0.005). Experimental replicates (n) = 6 (E, J, K), 4 (N). Scale bars = 100 μm (10x; F, H, L, M), 50 μm (40x; F, G, H and I), 100 μm (L) and 50 μm (D).

Figure S7

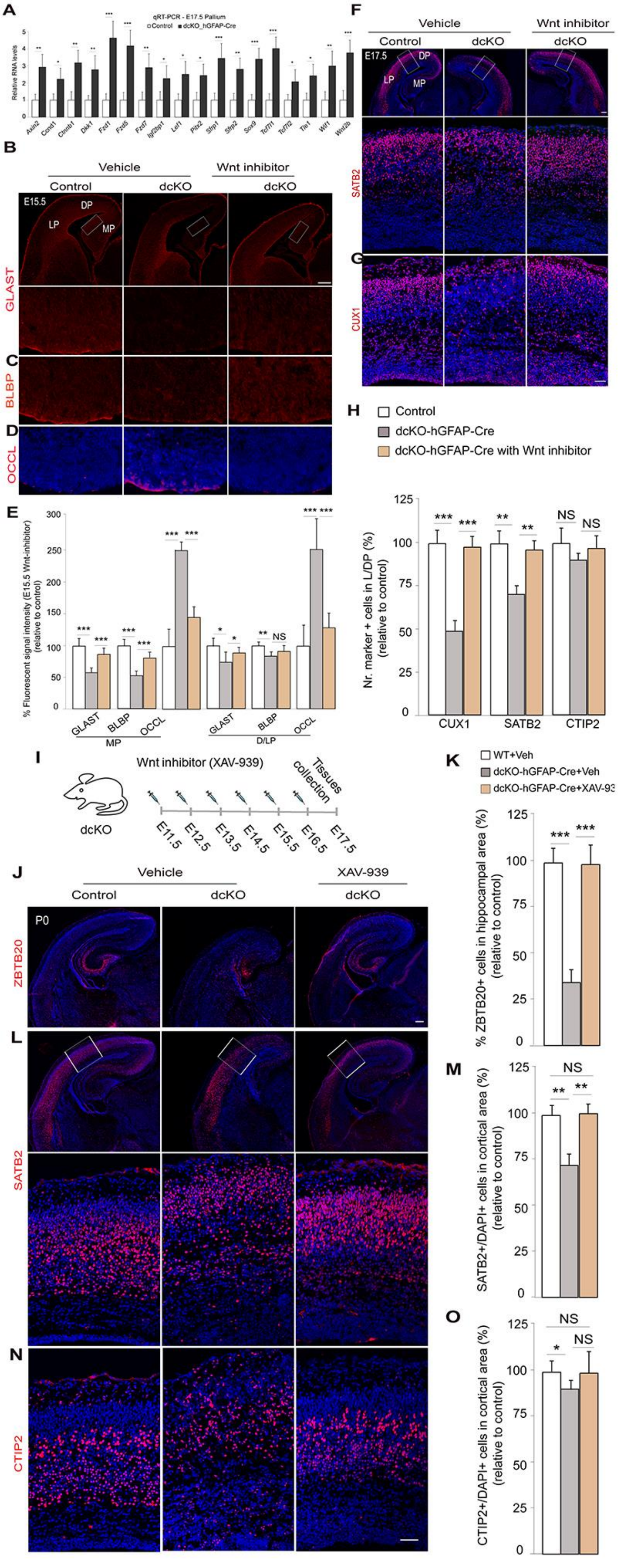

A
qRT-PCR - E17.5 Pallium
Control ■ dcKO_hGFAP-Cre
Relative RNA levels
Axin2 Ccnd1 Ctnnb1 Dkk1 Fzd1 Fzd5 Fzd7 Igf2bp1 Lef1 Pitx2 Sfrp1 Sfrp2 Sox9 Tcf7l1 Tcf7l2 Tle1 Wif1 Wnt2b
B
Vehicle
Wnt inhibitor
Control
dcKO
dcKO
E15.5
DP
LP
MP
GLAST
C
BLBP
D
OCCL
E
% Fluorescent signal intensity (E15.5 Wnt-inhibitor) (relative to control)
GLAST BLBP OCCL
MP
GLAST BLBP OCCL
D/LP
NS
F
Vehicle
Wnt inhibitor
Control
dcKO
dcKO
E17.5
DP
LP
MP
SATB2
G
CUX1
H
Control
dcKO-hGFAP-Cre
dcKO-hGFAP-Cre with Wnt inhibitor
Nr. marker + cells in L/DP (%) (relative to control)
CUX1
SATB2
CTIP2
NS
NS
I
Wnt inhibitor (XAV-939)
Tissues collection
dcKO
E11.5 E12.5 E13.5 E14.5 E15.5 E16.5 E17.5
K
WT+Veh
dcKO-hGFAP-Cre+Veh
dcKO-hGFAP-Cre+XAV-93
% ZBTB20+ cells in hippocampal area (%) (relative to control)
J
Vehicle
XAV-939
Control
dcKO
dcKO
P0
ZBTB20
L
SATB2
M
NS
SATB2+/DAPI+ cells in cortical area (%) (relative to control)
N
CTIP2
O
NS
NS
CTIP2+/DAPI+ cells in cortical area (%) (relative to control)

Figure S7 (related to figures 6, 7). Suppression of Wnt signaling in the developing pallium by BAF complexes in the developing forebrain.

(A) Expression of upregulated Wnt genes identified by RNA-Seq analysis (see Fig. 6A, B) was further verified by qRT-PCR. (B–E) IF (B–D) and quantitative (E) analyses of dcKO_*hGFAP*-Cre mutants at E15.5 showing the effects of treatment with the Wnt inhibitor ICG-001 on expression of the RG markers GLAST (B) and BLBP (C), and the NE marker OCCLUDIN (D). IF (F-G) and quantitative (H) analyses of dcKO_*hGFAP*-Cre mutants at E17.5, showing the effects of treatment with WNTi on pools of SATB2$^+$ (F), CUX1$^+$ (G) cortical neurons in L/DP. Quantitative analyses indicated that inhibition of Wnt signaling largely rescues defects in L/DP in dcKO_*hGFAP*-Cre mutants (H). (I–O) Rescued cortical defects in dcKO_hGFAP-Cre mutant by Wnt inhibitor XAV-939. (I) Experimental paradigm in which WT (control), dcKO embryos were treated with Wnt inhibitor XAV-939 or Vehicle (Veh). (J–O) IF (J, L, N) and quantitative (K, M, O) analyses are to compare population of cortical neuron: ZBTB20$^+$ hippocampal neurons (J, K), SATB2$^+$ late-born neurons (L, M), CTIP2$^+$ early-born neurons (N, O) in Veh-treated WT, Veh-treated dcKO and WNTi-treated WT. Quantitative analyses indicated that inhibition of Wnt signaling by XAV-939 largely rescues the neurogenesis defects in dcKO_*hGFAP*-Cre mutants. Values are expressed as means ± SEMs (*$P < 0.05$; **$P < 0.01$; ***$P < 0.005$; n = 4). Experimental replicates (n) = 4 (A, H, K, M, O), 6 (E). Abbreviations: MP, medial pallium; DP, dorsal pallium; LP, lateral pallium. Scale bars = 100 μm (10x; B, F), 50μm (40x; G), 100 μm (10x; J), and 50 μm (40x; N).

SUPPLEMENTAL EXPERIMENTAL PROCEDURES

Transgenic mice

BAF155$^{f/f}$ (Choi et al., 2012), *BAF170*$^{f/f}$ (Tuoc et al., 2013), *Emx1*-Cre (Gorski et al., 2002), *hGFAP*-Cre (Zhuo et al., 2001), *Nex*-Cre (Goebbels et al., 2006) and *Rosa-tdTomato (Ai9)(Madisen et al., 2010)* mice were maintained in a C57BL6/J background. Animals were handled in accordance with the German Animal Protection Law.

Plasmids

Plasmids used in this study: pCIG2-ires-eGFP, pCIG2-Cre-ires-eGFP (gift from Dr Francois Guillemot, NIMR London); 8XTOPFLASH (TOP) (Veeman et al., 2003) and Super8XFOPFLASH (FOP) (Veeman et al., 2003) were gifts from Randall Moon (Addgene plasmid # 12456, 12457)

Antibodies

The following polyclonal (pAb) and monoclonal (mAb) primary antibodies used in this study were obtained from the indicated commercial sources: AP2γ mouse mAb (1:100; Cat. ab87475, Abcam), BAF170 rabbit pAb (Cat. IHC-00213; Bethyl), BAF170 rabbit pAb (Cat. HPA021213; Sigma), BAF155 rabbit pAb (1:20; Cat. sc-10756; Santa Cruz), BAF155 mouse mAb (1:100; Cat. sc-48350X; Santa Cruz), Brn2 goat pAb (1:100; Cat. sc-6029, Santa Cruz), BLBP rabbit pAb (1:200; Cat. AB9558/ABN14; Chemicon), CASP3 rabbit pAb (1:100; Cat. #9661S; Cell Signaling), CTIP2 rat pAb (1:200; Cat. ab18465; Abcam), GLAST pig pAb (1:500; Cat. Af1000-1; Frontier), CIDU rat pAb (1:100; Cat. OBT-0030; Accurate), H3K27me3 rabbit pAb (Cat. 07-449; Upstate),

KDM6A/UTX rabbit pAb (Cat. sc-292326; Santa Cruz), KDM6B/jmjd3 rabbit pAb (De Santa et al., 2007), KI67 rabbit pAb (1:50; Cat. VP-RM04; Vector), HuCD mouse mAb (1:20; Cat. A21271; Invitrogen), LSD1 rabbit pAb (1:100; Cat. #2139s; Cell Signaling), SATB2 mouse mAb (1:200; Cat. ab51502; Abcam), SOX2 mouse mAb (1:100; Cat. MAB2018; R&D Systems), PROX1 rabbit pAb (1:1000; Cat. PRB-238C; Covance), PAX6 mouse mAb (1:100; Developmental Studies Hybridoma Bank), PAX6 rabbit pAb (1:200; Cat. PRB-278P; Covance), Flag mouse mAb (1:1000; Cat. F1804 Sigma), phospho-H3 rabbit mAb (1:200; Cat. Millipore), phospho-H3 rat pAb (1:300; Cat. Abcam), PVIM mouse mAb (1:500; Cat. D076-3 MBL), OCCLUDIN rabbit pAb (1:50; Cat. 40-4700 Thermo Fisher), TUBB3 mouse mAb (Tuj1, 1:500; Cat. Chemicon), TBR2 rabbit pAb (1:200; Cat. ab23345; Abcam), TBR1 rabbit pAb (1:300; Cat. AB9616; Chemicon), ZBTB20 rabbit pAb (1:50; Cat. HPA016815; Sigma), and RFP rabbit pAb (1:10000; Cat. 600-401-379; Biomol/Rockland).

Secondary antibodies used were horseradish peroxidase (HRP)-conjugated goat anti-rabbit IgG (1:10000; Cat. 111-035-003; Covance), HRP-conjugated goat anti-mouse IgG (1:5000; Cat. 115-035-003; Covance), HRP-conjugated goat anti-rat IgG (1:10000; Cat. 112-035-143; Covance), and Alexa 488-, Alexa 568-, Alexa 594- and Alexa 647-conjugated IgG (various species, 1:400; Molecular Probes).

Generation of dcKO mutants

To eliminate *BAF155* and *BAF170* in early cortical progenitors, late cortical progenitors, or projection neurons, we used the early progenitor-active *Emx1*-Cre (Gorski et al., 2002), late progenitor-active *hGFAP*-Cre (Zhuo et al., 2001) and neuron-specific *Nex*-Cre (Goebbels et al., 2006) mouse lines, respectively. Heterozygous

animals (*BAF155*$^{fl/+}$, *BAF170*$^{fl/+}$, *Cre*) were used as controls. Mutants crossed with *Emx1*-Cre or *hGFAP*-Cre died soon after birth.

Chromatin immunoprecipitation (ChIP)

ChIP assays performed on the pallium from control (n = 4) and dcKO_*hGFAP*-Cre E17.5 (n = 4) littermate embryos were performed as described previously (Narayanan et al., 2015). Briefly, tissues were homogenized in sucrose solution (0.32 M sucrose, 5 mM $Cacl_2$, 5 mM $Mg(Ac)_2$, 0.1 mM EDTA, 50 mM HEPES pH 8, 1 mM DTT, 0.1% Triton X-100), and then fixed in 37% formaldehyde. After stopping fixation by adding 1.25 M glycine, samples were washed with Nelson buffer (140 mM NaCl, 20 mM EDTA pH 8, 50 mM Tris pH 8, 0.5% NP-40, 1% Triton X-100) and sonicated in RIPA buffer (140 mM NaCl, 1 mM EDTA, 1% Triton X-100, 0.1% sodium deoxycholate, 10 mM Tris pH 8, 1% SDS).

For ChIP against histone marks 500 ng of chromatin with either 4 µg of anti-H3K4me2 antibody (Millipore) or 2 µg of anti-H3K27me3 antibody (Millipore) were used for each experiment, with input DNA of 50 ng. For ChIP against histone demethylases 10 µg of chromatin with either 10 µg of anti-LSD1 antibody (Abcam) or 10 µg of anti-JMJD3 antibody (Abcam) were used for each experiment with input DNA of 100 ng. After incubating overnight, samples were incubated with 15 µl of Protein A-coated beads (Diagenode) that had been blocked by incubating with 0.5% bovine serum albumen (BSA). Beads were washed with IP buffer (140 mM NaCl, 1% NP-40, 0.5% sodium deoxycholate, 50 mM Tris pH 8, 20 mM EDTA, 0.1% SDS) and wash buffer (100 mM Tris pH 8, 500 mM LiCl, 1% NP-40, 1% sodium deoxycholate, 20 mM EDTA), and chromatin was eluted with 0.1 µg/µl of RNase A diluted in 10 mM Tris (pH 8). De-

crosslinking was carried out in Wiemann buffer (100 mM Tris pH 8, 20 mM EDTA, 2% SDS) with Proteinase K (1 µg/µl). DNA was eluted in 10 mM Tris (pH 8).

ChIP-Sequencing

Libraries were prepared with a NEBNext Ultra DNA Library Prep Kit for Illumina (NEB) and analyzed using QuBit and an Agilent 2100 Bioanalyzer. Input DNA (50 ng) was isolated from each sample and then pooled separately for each group.

Base calling and FASTQ conversions were performed using standard Illumina scripts, as described previously (Halder et al., 2016; Narayanan et al., 2015). Quality control was also performed for each sample using FastQC (www.bioinformatics.babraham.ac.uk/projects/fastqc). Reads were mapped to the mm10 mouse reference genome using STAR aligner v2.3.0 (Djebali et al., 2012). BAM files were filtered leaving only high-quality reads [MAPQ !=(0,2,3,4)] as described previously (Halder et al., 2016).

BAM files of replicates from the same group were combined using the *merge* function of SAMTools (Li et al., 2009). Genomic profile plots were created from combined BAM files using NGSPlot (Shen et al., 2014). Wiggle (WIG) files were created from these BAM files using the script available in the MEDIPS package of Bioconductor (Lienhard et al., 2014). Visualization of individual gene loci was performed with Integrated Genome Browser (Nicol et al., 2009) using these WIG files.

Peaks were called using MACS2, with $q < 0.1$ (Feng et al., 2012). Differential binding analyses were performed using the DiffBind package of Bioconductor (Ross-Innes et al., 2012) with the DESEQ2 option for differential analysis. Peak annotation was performed using HOMER (Heinz et al., 2010) and homemade scripts.

RNA-Sequencing

RNA was extracted (RNeasy kit; Qiagen) from the pallium of control (n = 4) and dcKO_*hGFAP*-Cre (n = 3) E17.5 littermate embryos. cDNA libraries were prepared using the TruSeq RNA Sample Preparation v2 Kit. DNA was quantified using a Nanodrop spectrophotometer, and its quality was assessed using an Agilent 2100 Bioanalyzer.

Base calling, fastq conversion, quality control, and read alignments were all performed as outlined for ChIP-Seq. Reads were aligned to mouse genome mm10 and counted using FeaturesCount (http://bioinf.wehi.edu.au/featureCounts/). Differential expression was assessed using DESeq2 from Bioconductor (Love et al., 2014). Functional GO enrichment analyses were performed using ToppGene (Chen et al., 2009).

The high-throughput RNA-seq and ChIP-Seq data will be deposited in the NCBI Gene Expression Omnibus and made accessible through GEO Series accession numbers upon acceptance of the manuscript.

qPCR, ChIP-qPCR and Western blot analyses

qPCR and Western blot analyses were performed as described previously (Tuoc and Stoykova, 2008). Briefly, qPCR for confirmation of gene expression changes cDNA was synthesized using Transcriptor High Fidelity cDNA Synthesis Kit (Roche) and the results were normalized to housekeeping gene hypoxanthine phosphoribosyltransferase (*Hprt1*). For ChIP-qPCR the results were normalized against input DNA. All qPCR reactions were performed in Roche 480 Light Cycler using

SYBR Green.To validate the upregulated expression of Wnt-related genes in our RNA-Seq dataset, we performed qPCR using the mouse WNT Signaling Pathway RT2 Profiler PCR Array (PAMM-043Z; Qiagen) and mouse WNT Signaling Targets RT2 Profiler PCR Array (PAMM-243Z; Qiagen). The following primers were used for gene expression qPCR (5’ – 3’):

Primer	Sequence (5' - 3')
Adk_forward	CGCAGAATTCAATGAAAGTGG
Adk_reverse	GCATCCAAAGAATGTTGCTG
Adcy1_forward	AGATGGGACTTGACATGATCG
Adcy1_reverse	CGCATGTTCAGGTCTACTTCAG
Camk1d_forward	GAGTTTGATTCCCCCTACTGG
Camk1d_reverse	TCCATCAGATTCCGAATGAAG
Caprin2_forward	AGTTAAATGTAGAACCCAAAGATGTG
Caprin2_reverse	CCTCAATACTGGATCCTTTGGA
Ccnd1_forward	TTTCTTTCCAGAGTCATCAAGTGT
Ccnd1_reverse	TGACTCCAGAAGGGCTTCAA
Ctnnb1_forward	TGCAGATCTTGGACTGGACA
Ctnnb1_reverse	AAGAACGGTAGCTGGGATCA
Dkk1_forward	CCGGGAACTACTGCAAAAAT
Dkk1_reverse	CCAAGGTTTTCAATGATGCTT
Fuz_forward	CCTTAGAAACTTCTACACGCTGGT

Fuz_reverse	GGCTGGGTAGACTGTGTCCT
Kcnip2_forward	CTGCCCTCAGTCAGTGAAAA
Kcnip2_reverse	GTGGCACACCGTGGATAGTT
Pitx2_forward	CCTTACGGAAGCCCGAGT
Pitx2_reverse	AAAGCCATTCTTGCACAGC
Ptk2b_forward	CTCCTCCACAGACCAACCTG
Ptk2b_reverse	AGGAGAGCTGGCACACAGAC
Slc9a3r1_forward	CCCTTCAGCAATGGAGAGATAC
Slc9a3r1_reverse	TGGGGCTCTCTGAAGCTG
Tob1_forward	ACTTTTGCTGCCACCAAGTT
Tob1_reverse	GAGCTACCTTGCTGCTACGG
Unc5a_forward	CGTCATTGAACGCAGCAC
Unc5a_reverse	GATACGTTGATCCGGACCTC
Wnt2b_forward	GGGCCCTCATGAACTTACAC
Wnt2b_reverse	CCACTCACACCGTGACACTT
Hprt1_forward	GACAGGGACTCACTGCATAGTTT
Hprt1_reverse	GAGGCCAAGACAAGAAGACG
Others	Ordered from GeneGlobe, Qiagen (https://www.qiagen.com/us/geneglobe/)

And the following primers were used for ChIP-qPCR (5’ – 3’):

Primer	Sequence (5' - 3')
Adcy1_forward	GTGGCCCTTGCTGCATAC
Adcy1_reverse	CCAGCAGGCTCCTAACACC
Adk_forward	GCGCTCAGTGTCTGATGACTT
Adk_reverse	TGATGCGTCTATTTTTAGGTAGCA
Camk1d_forward	GGAAATGTTAAGTTTTCTTTGTAGCA
Camk1d_reverse	CAATTCAGCCGAACAGCTC
Caprin2_forward	TGCCCCACAGATGATTTTTC
Caprin2_reverse	TGGCGCATACCCTCCTACTA
Ccnd1_forward	GGGGCTTCTTTCCCTAAGAG
Ccnd1_reverse	CCTCCCTCCTAGCTGTCCTC
Ctnnb1_forward	TCCCAGGCTGAAGTCCTTAAT
Ctnnb1_reverse	ACTCTGTCCCAGGCAAGCTA
Dkk1_forward	GCTCCTCAGGGAAGACAACA
Dkk1_reverse	CGAGGGGAGAGTGTCAAAGT
Fuz_forward	CGTGGTTTTCATGATTTTGGA
Fuz_reverse	CCACCAAATGAGATGGTTGTT
Kcnip2_forward	GTGTCACACACGCCTACACC
Kcnip2_reverse	GGGCTGAGGAAGAACAGGT
Pitx2_forward	ACTCCGACCCGAAGAAGG

Pitx2_reverse	TGGTGGCAAGACGTCACTC
Ptk2b_forward	CAGTCCTGCAGAGACAACAGA
Ptk2b_reverse	GATGTGGCATGTGGCTTG
Slc9a3r1_forward	AGACATCCGTCCGGTTCTTA
Slc9a3r1_reverse	TGCTCCCCCACACTCACT
Tob1_forward	CACTCCGGACGATAACTCG
Tob1_reverse	ACTCTAGGGGCGCGGATA
Unc5a_forward	CATCCAGTTTGAATGGAGAGC
Unc5a_reverse	ATGGCTGACATCCACATTCTC
Wnt2b_forward	GGTACCACCGTAGGCAACC
Wnt2b_reverse	GGAGGGAACGTTGAGTCCTT

Co-immunoprecipitation and mass spectrometry (CoIP/MS)

BAF155 and BAF170 interaction analyses were performed using the neural stem cell line, NS5 (Conti et al., 2005), and E13.5 and E17.5 embryonic telencephalic tissue. Tissues were dissected and minced in cold phosphate-buffered saline (PBS) and then washed twice with PBS. Equivalent amounts of cells from one embryo were lysed for 30 min in 1 ml RIPA buffer containing a proteinase inhibitor cocktail (Roche) and DNase. All steps were performed at 4°C. Lysates were centrifuged for 10 min at 13,000 rpm to sediment out non-lysed tissues. The supernatant was pre-cleared by incubating with normal mouse IgG together with protein A/G-agarose beads, as described by the manufacturer (sc-2003; Santa Cruz). Interacting proteins were immunoprecipitated by

incubating pre-cleared supernatant with rabbit anti-BAF155 and anti-BAF170 antibodies and A/G-agarose beads. The beads were then washed first with 500 µl cold RIPA buffer (three times for 5 min each) and then with 40 µl of elution buffer (2.5 µl 20% SDS, 5 µl 1 M $NaHCO_3$, 42.5 µl double-distilled H_2O) for 15 min at room temperature.

For MS analyses (performed in the department of Prof. Dr. Henning Urlaub), samples were suspended in NuPage loading buffer and resolved on commercial SDS polyacrylamide gels (Novex NuPage Bis-Tris gel, 4–12% gradient; Invitrogen). Individual lanes were then cut into six squares for MS analysis. The parameters for the identification of proteins were set to the following values: limit, 95% probability of detection; limit of unique peptides detected, 1; and threshold detection probability of peptides, 80%.

The list of BAF155- and BAF170-interacting proteins revealed by MS analysis was obtained by subtracting nonspecific interactions with IgG in IPs and in telencephalic tissues from BAF155-null (*BAF155cKO_FoxG1*-Cre) and BAF170-null (*BAF170cKO_FoxG1*-Cre) mutants. The first set of controls excludes nonspecific binding to the antibody, and the second excludes nonspecific interactions that possibly could be precipitated by either the anti-BAF155 or anti-BAF170 antibody.

Injection of Wnt inhibitor (WNTi, ICG 001, XAV-939) and Wnt activator (WNTa, SB-216763), H3K27 demethylase inhibitor (GSK-J4), LSD1 histone demethylase (2-PCPA).

ICG001 (Tocris Bioscience, Cat. No. 4505), XAV-939 (Tocris Bioscience, Cat. No. 3748), SB-216763 (Tocris Bioscience, Cat. No. 1616), GSK-J4 (SIGMA, Cat. No. SML0701) and 2-PCPA (Tocris Bioscience, Cat. No. 3852) were dissolved in vehicle

(DMSO). 11.5 d.p.c. pregnant mice received daily injections of vehicle (150 µl), or ICG001 (150 µl of a 1-mg/ml solution), XAV-939, (150 µl of a 0.2 mg/ml solution), SB-216763 (100 µl of a 1-mg/ml solution plus 100 µl of saline), GSK-J4 (150µl of 2.5mg/ml solution)) and 2-PCPA (150µl of 5 mg/ml solution). Treated mice were sacrificed at different developmental stages as indicated in the text.

In vivo β-catenin transcriptional activity assay

In vivo and in vitro β-catenin transcriptional activity assay were performed as previously described (Durak et al., 2016; Mao et al., 2009). Briefly, Wnt/β-catenin transcriptional activity in vivo was monitored by electroporating brains of E13.5 *BAF155*$^{fl/fl}$;*BAF170*$^{fl/fl}$ embryos with a Cre plasmid (or empty plasmid as a control) and the reporter constructs Super8XTOPFLASH (TOP) or Super8XFOPFLASH (FOP) together with pRL-TK constructs at a 5:1:0.3 ratio. Wnt/β-catenin transcriptional activity was measured at E15.5. For in vitro assay, Neuro2A cells at 1×10^5 per well density were plated into 24-well plates. Cells were transfected with 0.8 µg of shRNA plasmids (shBAF155, shBAF170) along with 50 ng of Super8XTOPFLASH and 10 ng of pRL-TK. 2 days post-transfection, cells were collected for Wnt/β-catenin transcriptional activity measurement. In all cases, firefly luciferase activity was normalized to that of Renilla luciferase.

Demethylase assays

Cell culture-based demethylase activity assay for Utx/KDM6A and Jmjd3/KDM6B was performed previously (Narayanan et al., 2015) .

For Histone demethylase KDM1/LSD1 activity quantification, cultured dcKO_CAG-Cre NSCs were nucleofected with a mammalian expression vector for LSD1/KDM1A. The Cre-mediated deletion of BAF155 and BAF170 alleles was induced by adding TAM to the growth medium at a final concentration of 1 µM. After 2 DIV, The LSD1 demethylation assay was performed by using nuclear extracts from above NSCs and Epigenase LSD1 Demethylase activity Kit according to manufacturer (Epigentek)'s recommendations (Katz et al., 2014).

IF and cell cycle parameters

IF experiments and determination of cell cycle indexes were carried out as previously described (Narayanan et al., 2015; Tuoc and Stoykova, 2008).

Spindle angle analysis

Brain sections were stained with PVIM to outline the cell shape and PHH3 to identify anaphase and early telophase dividing cells. Images of z-stack sections were obtained by SP5 confocal microscopy, and 3D reconstruction of the confocal stacks was done as described previously (Postiglione et al., 2011; Tuoc et al., 2013).

3D reconstruction and cell counting

3D images of the developing hippocampus were constructed using Neurolucida software version 11.03. Consecutive sections (25 µm each) of the E15.5 WT and dcKO hippocampus were imaged in rostro-caudal order. Triple IF with antibodies against ZBTB20, PAX6 and PHH3 was performed to quantify the volume of the hippocampal plate ($ZBTB20^+$/$PAX6^-$), hippocampal neuroepithelium ($ZBTB20^-$/$PAX6^+$), and total

number of $pHH3^+$ M-phase cells in the apical surface of the developing hippocampus. Contours were drawn in each section based on the expression of the hippocampus-specific marker Ztbt20. The 3D reconstruction was produced from whole-stack contours. The volume analysis was done using Neurolucida Explorer v. 11.03.

Cell counts and quantitative analysis of IF signal intensity

Immunostaining in IF images was quantified using anatomically matched forebrain sections. Nucleus-marker–positive cells within the pallium of confocal images were counted for comparison. In most cases, cell counts of six matched sections were averaged from three biological replicates (control/dcKO pallium). In many cases, the number of lineage marker cells was quantified using total marker-positive cells alone, or by normalizing to the total number of $DAPI^+$ (nucleus-stained) cells using the following equation: Normalized number = marker-positive cell number/$DAPI^+$ cell number. Statistical analyses of histological data were performed using Student's *t*-test. All bar graphs are plotted as means ± SEM. All statistical tests are two-tailed, and *P*-values are considered to be significant for $\alpha = 0.05$. All details of statistical analyses of histological experiments are presented in Table S6.

Statistical Analysis

Statistical analyses were designed using the assumption of normal distribution and similar variance among groups, as previously tested. The sample size was determined based on preliminary results or similar experiments carried-out in the past. Power analysis was performed using G-power in order to estimate the number of animals required, for a signal-to-noise ratio of 1.4 and 80% to 90% power assuming a 5%

significance level. For histological analysis, qPCR, ChIP-qPCR and comparison of genome-wide histone marks, statistics were assessed with Student's *t*-test. The significance of overlaps was assessed using hypergeometric test. RNA-Seq and ChIP-Seq analyses were carried out using DESeq2 and DiffBind packages of Bioconductor respectively, with their own in-built statistical calculation tools. The results are presented as means ± SEM. *P* values of < 0.05 were considered to be statistically significant unless otherwise indicated. All the relevant information pertaining to statistical analysis is also specified in each figure legend separately. A detailed description of quantitative analysis methods is presented in Supplemental Information. The statistic quantification was carried out as average from at least three biological replicate. All details of statistical analyses for histological experiments are presented in Table S6.

SUPPLEMENTAL REFERENCES

Chen, J., Bardes, E.E., Aronow, B.J., and Jegga, A.G. (2009). ToppGene Suite for gene list enrichment analysis and candidate gene prioritization. Nucleic acids research *37*, W305-311.

Choi, J., Ko, M., Jeon, S., Jeon, Y., Park, K., Lee, C., Lee, H., and Seong, R.H. (2012). The SWI/SNF-like BAF complex is essential for early B cell development. J Immunol *188*, 3791-3803.

Conti, L., Pollard, S.M., Gorba, T., Reitano, E., Toselli, M., Biella, G., Sun, Y., Sanzone, S., Ying, Q.L., Cattaneo, E*., et al.* (2005). Niche-independent symmetrical self-renewal of a mammalian tissue stem cell. PLoS Biol *3*, e283.

De Santa, F., Totaro, M.G., Prosperini, E., Notarbartolo, S., Testa, G., and Natoli, G. (2007). The histone H3 lysine-27 demethylase Jmjd3 links inflammation to inhibition of polycomb-mediated gene silencing. Cell *130*, 1083-1094.

Djebali, S., Davis, C.A., Merkel, A., Dobin, A., Lassmann, T., Mortazavi, A., Tanzer, A., Lagarde, J., Lin, W., and Schlesinger, F. (2012). Landscape of transcription in human cells. Nature *489*, 101-108.

Durak, O., Gao, F., Kaeser-Woo, Y.J., Rueda, R., Martorell, A.J., Nott, A., Liu, C.Y., Watson, L.A., and Tsai, L.H. (2016). Chd8 mediates cortical neurogenesis via transcriptional regulation of cell cycle and Wnt signaling. Nat Neurosci.

Feng, J., Liu, T., Qin, B., Zhang, Y., and Liu, X.S. (2012). Identifying ChIP-seq enrichment using MACS. Nature protocols *7*, 1728-1740.

Goebbels, S., Bormuth, I., Bode, U., Hermanson, O., Schwab, M.H., and Nave, K.A. (2006). Genetic targeting of principal neurons in neocortex and hippocampus of NEX-Cre mice. Genesis *44*, 611-621.

Gorski, J.A., Talley, T., Qiu, M., Puelles, L., Rubenstein, J.L., and Jones, K.R. (2002). Cortical excitatory neurons and glia, but not GABAergic neurons, are produced in the Emx1-expressing lineage. J Neurosci *22*, 6309-6314.

Halder, R., Hennion, M., Vidal, R.O., Shomroni, O., Rahman, R.-U., Rajput, A., Centeno, T.P., van Bebber, F., Capece, V., and Vizcaino, J.C.G. (2016). DNA methylation changes in plasticity genes accompany the formation and maintenance of memory. Nature neuroscience *19*, 102-110.

Heinz, S., Benner, C., Spann, N., Bertolino, E., Lin, Y.C., Laslo, P., Cheng, J.X., Murre, C., Singh, H., and Glass, C.K. (2010). Simple combinations of lineage-determining transcription factors prime cis-regulatory elements required for macrophage and B cell identities. Molecular cell *38*, 576-589.

Katz, T.A., Vasilatos, S.N., Harrington, E., Oesterreich, S., Davidson, N.E., and Huang, Y. (2014). Inhibition of histone demethylase, LSD2 (KDM1B), attenuates DNA methylation and increases sensitivity to DNMT inhibitor-induced apoptosis in breast cancer cells. Breast Cancer Res Treat *146*, 99-108.

Li, H., Handsaker, B., Wysoker, A., Fennell, T., Ruan, J., Homer, N., Marth, G., Abecasis, G., and Durbin, R. (2009). The sequence alignment/map format and SAMtools. Bioinformatics *25*, 2078-2079.

Lienhard, M., Grimm, C., Morkel, M., Herwig, R., and Chavez, L. (2014). MEDIPS: genome-wide differential coverage analysis of sequencing data derived from DNA enrichment experiments. Bioinformatics *30*, 284-286.

Love, M.I., Huber, W., and Anders, S. (2014). Moderated estimation of fold change and dispersion for RNA-seq data with DESeq2. Genome biology *15*, 1.

Madisen, L., Zwingman, T.A., Sunkin, S.M., Oh, S.W., Zariwala, H.A., Gu, H., Ng, L.L., Palmiter, R.D., Hawrylycz, M.J., Jones, A.R.*, et al.* (2010). A robust and high-throughput Cre reporting and characterization system for the whole mouse brain. Nat Neurosci *13*, 133-140.

Mao, Y., Ge, X., Frank, C.L., Madison, J.M., Koehler, A.N., Doud, M.K., Tassa, C., Berry, E.M., Soda, T., Singh, K.K.*, et al.* (2009). Disrupted in schizophrenia 1 regulates neuronal progenitor proliferation via modulation of GSK3beta/beta-catenin signaling. Cell *136*, 1017-1031.

Narayanan, R., Pirouz, M., Kerimoglu, C., Pham, L., Wagener, R.J., Kiszka, K.A., Rosenbusch, J., Seong, R.H., Kessel, M., Fischer, A.*, et al.* (2015). Loss of BAF (mSWI/SNF) Complexes Causes Global Transcriptional and Chromatin State Changes in Forebrain Development. Cell Rep *13*, 1842-1854.

Nicol, J.W., Helt, G.A., Blanchard, S.G., Raja, A., and Loraine, A.E. (2009). The Integrated Genome Browser: free software for distribution and exploration of genome-scale datasets. Bioinformatics *25*, 2730-2731.

Postiglione, M.P., Juschke, C., Xie, Y., Haas, G.A., Charalambous, C., and Knoblich, J.A. (2011). Mouse inscuteable induces apical-Basal spindle orientation to facilitate intermediate progenitor generation in the developing neocortex. Neuron *72*, 269-284.

Ross-Innes, C.S., Stark, R., Teschendorff, A.E., Holmes, K.A., Ali, H.R., Dunning, M.J., Brown, G.D., Gojis, O., Ellis, I.O., and Green, A.R. (2012). Differential oestrogen receptor binding is associated with clinical outcome in breast cancer. Nature *481*, 389-393.

Shen, L., Shao, N., Liu, X., and Nestler, E. (2014). ngs. plot: Quick mining and visualization of next-generation sequencing data by integrating genomic databases. BMC genomics *15*, 1.

Tuoc, T.C., Boretius, S., Sansom, S.N., Pitulescu, M.E., Frahm, J., Livesey, F.J., and Stoykova, A. (2013). Chromatin Regulation by BAF170 Controls Cerebral Cortical Size and Thickness. Developmental Cell *25*, 256-269.

Tuoc, T.C., and Stoykova, A. (2008). Trim11 modulates the function of neurogenic transcription factor Pax6 through ubiquitin-proteosome system. Genes & development *22*, 1972-1986.

Veeman, M.T., Slusarski, D.C., Kaykas, A., Louie, S.H., and Moon, R.T. (2003). Zebrafish prickle, a modulator of noncanonical Wnt/Fz signaling, regulates gastrulation movements. Current biology : CB *13*, 680-685.

Zhuo, L., Theis, M., Alvarez-Maya, I., Brenner, M., Willecke, K., and Messing, A. (2001). hGFAP-cre transgenic mice for manipulation of glial and neuronal function in vivo. Genesis *31*, 85-94.
